# Origins of the hydrogen spillover effect in *d*-block metals

**DOI:** 10.1038/s41467-026-72608-0

**Published:** 2026-05-11

**Authors:** Yang Li, Yuanming Zhang, Zhaojian Zeng, Yong Chen, Xiaoming Xu, Zhigang Zou, Zhaosheng Li

**Affiliations:** 1 Collaborative Innovation Center of Advanced Microstructures, National Laboratory of Solid State Microstructures, College of Engineering and Applied Sciences, Nanjing University, Nanjing, China; 2 Jiangsu Key Laboratory of Nano Technology, Nanjing University, Nanjing, China

**Keywords:** Heterogeneous catalysis, Catalytic mechanisms, Reaction kinetics and dynamics

## Abstract

The hydrogen spillover effect embodies a sophisticated and multifaceted phenomenon for H atom equivalents. Despite substantial efforts to identify potential candidates, including *d*-block metals like Ru and Pd, fundamental aspects of dynamic interplay of activation and spillover processes are still inadequately understood, hindering efficient hydrogenation reactions and high-capacity chemical hydrogen storage. Here, we quantify a comprehensive platform of thermodynamic‒kinetic descriptors to systematically elucidate the full scope of the hydrogen spillover pathway and transcend the limitations of conventional approaches that focus solely on the surface concentration of spilled-over hydrogen, shielding the signal heterogeneity inherent in zones of differential hydrogen availability. Taking a well-defined *d*-block M/TiO_2−*x*_ (M = Ru, Mn, Fe, Co, or Ni) as a representative case by combining spectroscopic quantification and computational investigation, the nonlinear spillover capability and rate of *d*-block metal originates from the synergistic overlap of its unoccupied *d*-*s* orbitals, and the σ and σ* orbitals of the H atom equivalents interact to form a weak antibonding state, reducing the dissociation of the H–H bond and M–H bond. This methodology provides a refined lens for dissecting spillover mechanisms, facilitating a profound mechanistic and spatial understanding of rational metal dilution.

## Abstract (web summary)

Efficient hydrogen storage and catalysis rely on understanding how hydrogen atoms move across catalyst surfaces. This study identifies electronic descriptors that govern hydrogen spillover, enabling the rational design of high-performance catalysts.

## Introduction

Hydrogen spillover refers to the phenomenon in which hydrogen atoms, generated through the dissociative adsorption of H_2_ on active surfaces, such as metals, spontaneously migrate to adjacent surfaces where H_2_ is less prone to dissociate^1–4^. Since its initial demonstration in experiments conducted by Khoobiar in 1964, the potential of hydrogen spillover has been recognized in elucidating numerous experimental phenomena observed in multiphase catalytic hydrogenation reactions. This recognition has sparked a substantial number of associated investigations^5–8^. Currently, hydrogen spillover is closely associated with multiphase catalysis, semiconductor surface chemistry, and light-driven hydrogen evolution. It has played a crucial role in various fields, such as Fischer‒Tropsch synthesis^9^, CO_2_ reduction^10,11^, catalyst deactivation^12^, and hydrogen production and storage^13–15^.

Precise interpretation and effective control of hydrogen activation and spillover are crucial for catalytic reactions involving hydrogen^16–18^. In the current research landscape, significant advancements in deducing aspects of the hydrogen spillover process have been made through various techniques. By performing nanoscale resolved X-ray absorption spectroscopy via X-ray photoemission electron microscopy, Karim and coworkers reported that, compared with reducible TiO_2_, hydrogen spillover on nonreducible Al_2_O_3_ is significantly slower and confined to shorter distances^19^. Chandler et al. highlighted that the spillover phenomenon is an entropy-driven adsorption process closely linked to the carrier’s ability to stabilize surface protons and subsurface electrons^20^. By employing high-pressure scanning tunneling microscopy and X-ray photoelectron spectroscopy, Liu et al. reported that the direction of spillover is effectively governed by the surface structure^21^. Despite extensive research on hydrogen spillover providing valuable insights into chemical reactions, the comprehension of the hydrogen spillover process remains inadequate and insensitive for the spillover hydrogenation pathway, primarily due to the lack of effective approaches to directly visualize the entire hydrogen spillover process and systematic quantitative analysis with comprehensive criteria. Therefore, the ongoing advancements in promising metals for hydrogenation and high-capacity chemical hydrogen storage remain limited. And in the hydrogen spillover process, the activation zone is where H_2_ molecules are dissociated into atomic hydrogen, while the spillover zone corresponds to the migration and utilization of these species on the support surface. However, these two zones are often coupled and difficult to distinguish experimentally.

In this work, we decouple the activation and spillover zones through quantitative analysis of photoexcitation reduction test on *d*-block metal-modified TiO_2−*x*_ (TiO_2_ is a classic semiconductor with high stability, inertness, and resistance to photo corrosion, offering reliable performance and broad scalable applications) under light-driven conditions. These two zones are assessed using previously undescribed thermodynamic-kinetic descriptors, including the spillover capacity (Γ), specific spillover capacity (Γ^*^), activation degree (Ф) for the activation zone, and average spillover rate (κ) for the spillover zone. A statistical comparison and theoretical calculation analysis of *d*-block Ru, Mn, Fe, Co and Ni revealed that the extraordinary hydrogen spillover capability of Ru can be attributed to its unparalleled hydrogen activation efficiency. Integrated with theoretical calculations and molecular dynamics, the nonlinear hydrogen spillover capability and rate observed for *d*-block metals originate from the intrinsic properties of the metal *d–s* orbitals. The partially vacant *d–s* orbitals can synergistically overlap with the σ and σ* orbitals of adsorbed hydrogen, forming weak antibonding states that partially weaken both the H–H and M–H bonds. This orbital-level interaction lowers the energy barriers for hydrogen dissociation and migration, providing a mechanistic explanation for the enhanced spillover behavior of *d*-block metals.

## Results

### Quantification platform for hydrogen spillover

**Fig. 1:**
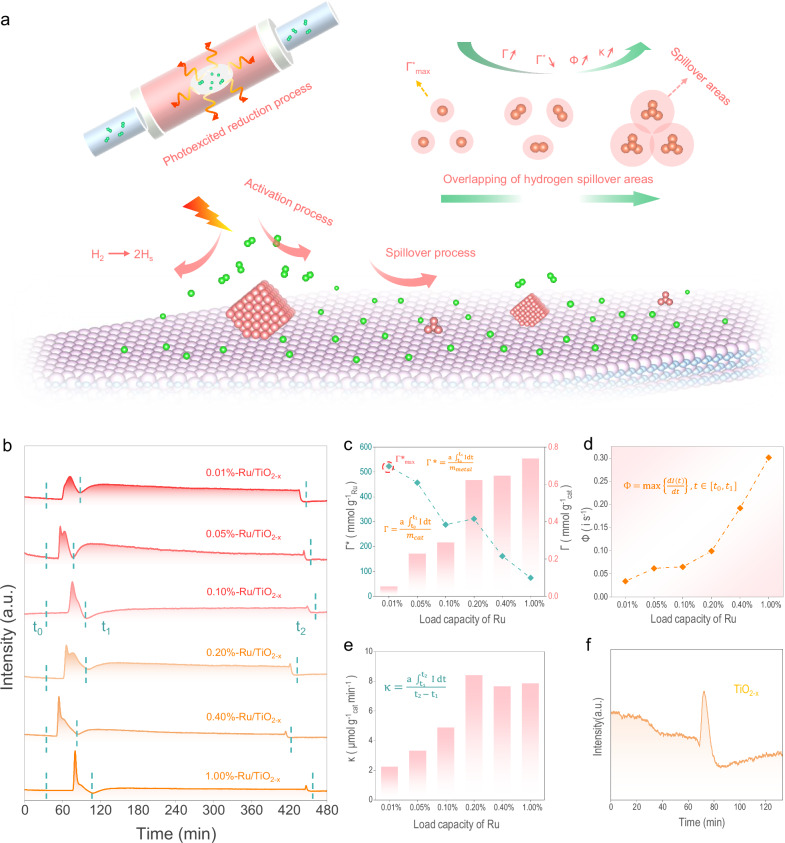
Defining the quantification platform for hydrogen spillover. **a** Schematics representing the hydrogen spillover process via instructive thermodynamic‒kinetic descriptors, where Γ represents the spillover capacity, Γ^*^ represents the intrinsic spillover capacity, Ф represents the activation degree and κ represents the spillover rate. The green balls represent hydrogen atoms. Bright pink balls represent metal atoms. **b** Photoexcited reduction tests of Ru/TiO_2−*x*_ samples with varying Ru loadings. **c** Statistics of Γ and Γ^*^ for all the samples. **d** Statistics of Ф for all the samples. **e** Statistics of κ for all the samples. **f** Photoexcited reduction test of the TiO_2−*x*_ sample.

Hydrogen spillover predominantly transpires within supported catalyst systems. Typically, in contrast to non-metallic supports, H_2_ is readily activated and dissociated on metal surfaces. Following this, the dissociated hydrogen atoms (H_s_) spill over from the metal surface to the support surface, bestowing an equilibrium of spillover. To aptly depict the hydrogen spillover process, we delineate it into two distinct processes: the activation process (rapid process) and the spillover process (slow process), which are arranged in chronological sequence (Fig. 1a). The activation process entails the conversion of H_2_ on the metal surface into individual H_s_. Given the low activation energy of H_2_ on metal surfaces, this transformation occurs swiftly within a brief timeframe. The spillover process involves the migration of H_s_ from the metal surface to the support surface. The pace of this phenomenon is governed by the diffusion concentration gradient, which renders the diffusion process comparatively slower than the activation process (Fig. 1a).

As the protagonist of the spillover process, the free H_s_ are supplied primarily by the activation process. The quantity of H_2_ activated during the activation process was defined as the epitome of a sample’s spillover capacity (Γ), as described in formula 1:

1 $$\Gamma=\frac{a{\int }_{{t}_{0}}^{{t}_{1}}Idt}{{m}_{{{{\rm{cat}}}}}}$$

where Γ represents the spillover capacity, *I* represents the intensity of the photoexcited reduction test, *a* represents the normalization factor, whose physical significance lies in converting the measured signal intensity into the corresponding quantity of gas. To establish the quantitative standard, we repeatedly introduced a fixed amount of gas and collected the associated signal responses to derive a calibration equation. The coefficient a in this equation depends solely on the properties of the detector and is independent of the metal. The *t*_0_ and *t*_1_ represent the time points. As the activation process is facilitated primarily by the metal, we standardize the spillover capacity relative to the percentage of metal loading, and this normalization is defined as the specific spillover capacity (Γ^*^) of the active metal, as described in formula 2:

2 $${\Gamma }^{\ast }=\frac{a{\int }_{{t}_{0}}^{{t}_{1}}Idt}{{m}_{{{{\rm{metal}}}}}}$$

where *m*_metal_ represents the loading amount of the metal. With the same support, the Γ^*^ values of identical metal atoms remain uniform. When metal atoms are widely dispersed without interacting with each other, their Γ^*^ reaches its peak (Γ^*^_max_). However, as metal atoms approach each other closely or aggregate into clusters, the activation zones overlap, leading to a reduction in Γ^*^. As the distance between metal atoms decreases further and the size of the clusters increases, Γ^*^ progressively decreases. Different atoms exhibit varying activation strengths for H_2_, and the activation strengths of the same atoms differ at various loading contents. Consequently, we introduce the concept of activation degree (Ф) as a metric for assessing the strength of H_2_ activation, as shown in formula 3:

3 $$\Phi=\max \left\{\frac{{dI}(t)}{{dt}}\right\},t\in \left[{t}_{0}\right.,\left.{t}_{1}\right]$$

Regarding the spillover process, we also define the average spillover rate (κ) to evaluate the spillover capability of the sample, as described in the following formula 4.

4 $$\kappa=\frac{a{\int }_{{t}_{1}}^{{t}_{2}}Idt}{{t}_{2}-{t}_{1}}$$

where *I* is the intensity of the photoexcited reduction test, *a* is the normalization factor, and *t*_1_ and *t*_2_ represent the time scales. During the photoexcited reduction (PER) experiment, once the test solid reaches adsorption saturation in a flowing hydrogen atmosphere, real-time monitoring of changes in detection signals before and after light exposure not only enables a comprehensive display of the activation and spillover processes of hydrogen but also facilitates quantitative analysis and comparison (Fig. 1a).

The test results of samples with different Ru loadings exhibit two distinct processes, which is consistent with the definitions mentioned above. As shown in Fig. 1b, we observe a sharp, rapid hydrogen-fragmentation peak (activation zone, *t*_0_ to *t*_1_) and a broader, slower diffusion peak (spillover zone, *t*_1_ to *t*_2_). The number of H_2_ molecules available in the gas phase exceeds the instantaneous activation capacity of the metal sites on the catalyst surface. At any moment, only a fraction of H_2_ molecules can be adsorbed and dissociated on the metal, generating H_s_. These H_s_ subsequently migrate from the metal surface to the catalyst support via hydrogen spillover. Meanwhile, newly exposed metal sites continuously activate additional H_2_ molecules. H_2_ dissociation on the metal and hydrogen spillover to the support proceed concurrently and establish a dynamic steady state, enabling the continuous activation of the hydrogen. So, the “activation” and “spillover” zones represent kinetically distinguishable and decouplable components, consistent with the framework of transient kinetic and pulse-response analyses. As the Ru loading increases from 0.01% to 1%, the Γ of the catalyst rapidly increases, followed by a slower increase in the later stages (Fig. 1c). However, their Γ^*^ values continue to decrease due to the overlap and interference of activation regions as Ru atoms transition from fully dispersed isolated entities to clustered forms. Additionally, we can infer the Γ^*^_max_ of the catalyst to be 523.3 mmol g^−1^_Ru_. Fig. 1d shows a continual improvement in the activation degree (Ф) as the Ru loading of the catalysts increases. During the spillover process, the κ of all the samples gradually increases at first, followed by reaching a state of equilibrium. This indicates that the spillover rate is affected by the spillover capacity, but it becomes saturated once the loaded metal reaches a certain coverage level (Fig. 1e). In comparison, during the PER testing process, TiO_2−*x*_ did not display a complete hydrogen spillover process. Instead, only a very faint signal peak was observed, suggesting its limited ability to activate H_2_ (Fig. 1f). The computational details are explained by taking 1.00%-Ru/TiO_2−*x*_ as a representative example in Supplementary Note. The corresponding values for the other samples are listed in Supplementary Table 1. As shown in Supplementary Fig. 1, to further elucidate the influence of support types, we conducted a comparative study using Ru/TiO_2_ as a reference. The results show that, although Ru/TiO_2_ can also activate hydrogen, its hydrogen adsorption capacity and spillover kinetics are lower than those of Ru/TiO_2−*x*_, owing to the enhanced electronic conductivity and light absorption of TiO_2−*x*_. Additionally, we also carried out modulation experiments under different light intensities. With decreasing light intensity, the signal resolution of both activation and spillover showed a slight decline, yet the two regions remained clearly distinguishable (Supplementary Fig. 2). These results further validate the robustness of the photoexcited reduction test.

### Visualization of the hydrogen spillover process

**Fig. 2:**
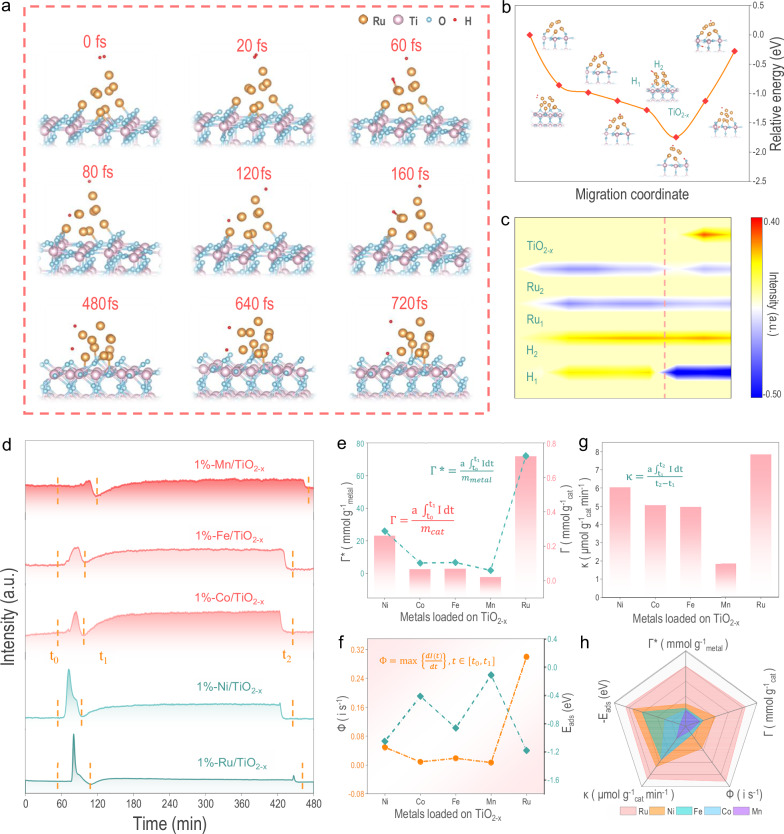
Simulation-assisted hydrogen spillover visualization and universal analysis of *d*-block metals. **a** Molecular dynamics simulation visualizing the process of hydrogen spillover. **b** Change in the Gibbs free energy of the hydrogen spillover process on the Ru/TiO_2−*x*_ sample. **c** Bard charge transfer during the hydrogen spillover process on the Ru/TiO_2−*x*_ sample. H_1_ and H_2_ denote the hydrogen atoms in a hydrogen molecule, while Ru_1_ and Ru_2_ are simply labels within the Ru cluster without specific physical significance. **d** Photoexcited reduction tests for the Mn/TiO_2−*x*_, Fe/TiO_2−*x*_, Co/TiO_2−*x*_, Ni/TiO_2−*x*_ and Ru/TiO_2−*x*_ samples. **e** Statistics of Γ and Γ^*^ for all the samples. **f** Statistics of Ф and hydrogen dissociation energy for all the samples. E_ads_ is the hydrogen adsorption energy calculated by DFT. **g** Statistics of κ for all the samples. **h** Comparison of descriptors (Γ, Γ^*^, Ф, κ and E_ads_) for all the samples.

Molecular dynamics simulations provide a visual representation of the hydrogen spillover process on Ru/TiO_2−*x*_. The simulation involved placing a single hydrogen molecule at the Ru site and running the system at 750 K for approximately 720 femtosecond (fs) (Fig. 2a). Initially, H_2_ molecules in the environment adsorb onto the surface of Ru clusters and rapidly dissociate, forming Ru–H bonds that correspond to the activation zone of the hydrogen spillover effect. Subsequently, the hydrogen atoms rapidly diffuse across the interface to the support, forming Ti–O–H species, which correspond to the spillover zone (Fig. 2a and Supplementary Fig. 3). Conversely, the migration of hydrogen from the support into the interior is energetically unfavorable (Fig. 2b), indicating that the hydrogen spillover primarily occurs on the interface. Combined with density functional theory (DFT) calculations, we analyzed the energy and charge transfer processes: during H_2_ activation on the Ru surface, charge transfers from Ru to hydrogen. One hydrogen atom (H_2_) remains on the Ru cluster, while the other (H_1_) spills over to the support, transferring charge to the support surface (Fig. 2c). This behavior is consistent with the activation and spillover processes observed in our PER experiments.

To verify the universality of the spillover process and validate the quantitative criteria, we conducted PER measurements on diverse solid catalysts under identical conditions (Supplementary Figs. 4 and 5). Mn/TiO_2−*x*_, Fe/TiO_2−*x*_, Co/TiO_2−*x*_, Ni/TiO_2−*x*_ and Ru/TiO_2−*x*_ were confined in quartz tubes and illuminated with continuous light. The catalyst average temperatures were continuously monitored in real time and maintained within a narrow range of 466 °C–468 °C, once thermal uniformity was achieved, a flowing Ar/H_2_ mixture was introduced. They all clearly demonstrate the complete activation and spillover processes (Fig. 2d). During activation, the Γ and Γ* values (Fig. 2e) as well as the Ф values (Fig. 2f) of these samples follow a consistent trend that correlates with E_ads_, the hydrogen adsorption energy calculated by DFT, indicating a positive correlation between these parameters and the absolute value of the dissociation energy of H_2_. In addition, during the spillover process, we observed that the κ value gradually increases with the metal atomic number (Fig. 2g). This trend arises because the degree of *d–s* hybridization progressively increases from Mn (3*d*^5^4*s*^2^) to Ru (4 *d*^7^5*s*^1^) among the *d*-block metals, whereas for Pd the *d–s* hybridization decreases owing to its full-shell 4*d*^10^5*s*^0^ configuration and the deep energetic stabilization of the 4*d* band (Supplementary Fig. 6). Fig. 2h provides a clearer comparison of the relationship between the hydrogen spillover quantification parameters (Γ, Γ*, Φ, and κ) and the metal’s H_2_ dissociation ability. These quantitative descriptors reveal the pronounced nonlinearity of both spillover capacity (Γ) and spillover rate (κ) within the hydrogen spillover dual zones. Moreover, metals with stronger H_2_ activation capability exhibit correspondingly stronger spillover behavior, which increases with the degree of *d–s* orbital hybridization. This finding further supports the validity and applicability of our previously proposed theoretical framework. Such quantitative insights can help minimize precious-metal usage and guide the rational design of cost-effective catalysts.

### Structural and chemical heterogeneity

**Fig. 3:**
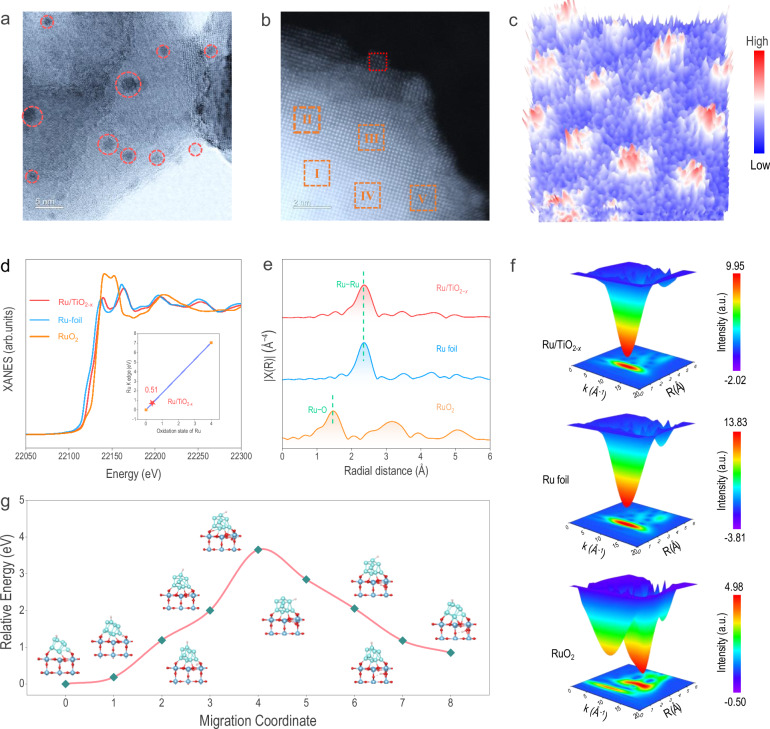
Structural and chemical heterogeneity of Ru/TiO_2−*x*_. **a**,**b** HAADF-STEM images of Ru/TiO_2−*x*_. **c** Three-dimensional intensity surface plot of Ru nanoclusters shown in the red dashed regions in (**b)**. **d** XANES spectra at the Ru K-edge of Ru/TiO_2−*x*_, Ru foil and RuO_2_. The inset reveals the Ru oxidation state via XANES spectroscopy. **e** FT-EXAFS spectra at the Ru K-edge of Ru/TiO_2−*x*_, Ru foil and RuO_2_. **f** Wavelet transformation of Ru/TiO_2−*x*_ based on the k^2^-weighted EXAFS signal. **g** Change in the Gibbs free energy of the hydrogen atom migration into the support on the Ru/TiO_2−*x*_ sample. To distinguish the Gibbs free energy pathways of hydrogen spillover activation and surface H migration discussed earlier, an adjusted color scheme is used: light pink spheres for H, light cyan for Ru, red for O, and light blue for Ti atoms.

X-ray diffraction (XRD) patterns (Supplementary Fig. 7) demonstrated that the crystallinity of TiO_2−*x*_ remains essentially unchanged before and after metal modification. Transmission electron microscopy (TEM) images (Supplementary Fig. 8) revealed the presence of metal nanoclusters (Mn, Fe, Co, Ni) uniformly distributed on the TiO_2−*x*_ surface. The atomic-scale structure of the Ru/TiO_2−*x*_ can be observed via the aberration-corrected high-angle annular dark field scanning TEM (HAADF-STEM) technique, which show that distinct interfaces were formed between the Ru nanoclusters and the TiO_2−*x*_ support (Fig. 3a, b). Different regions of the TiO_2−*x*_ support display well-defined atomic-scale lattice fringes (Supplementary Fig. 9), also indicating a high degree of crystallinity. Moreover, the Energy dispersive X-ray (EDX) mapping (Supplementary Fig. 10) confirmed that the distributions of the elements Ru, Ti and O were uniform. We also calculated the atomic variation coefficients of the Ru nanocluster in the *x* and *y* directions (Supplementary Figs. 11 and 12), both of which are very small, indicating a highly ordered lattice and excellent structural stability (Fig. 3c).

To gain a deeper understanding of the unique electronic structure and the cluster–support interactions in the Ru/TiO_2−*x*_ catalyst, X-ray absorption spectroscopy (XAS) was carried out. For Ru K-edge X-ray absorption near-edge structure (XANES), the analysis revealed that the average valence state of Ru in Ru/TiO_2−*x*_ was approximately 0.51 (Fig. 3d), indicating that most of the Ru species in the catalyst were present as metallic Ru^0^. Furthermore, Fourier-transform extended X-ray absorption fine structure (FT-EXAFS) spectra (Fig. 3e, Supplementary Figs. 13 and 14) were acquired to elucidate the local coordination environment of the Ru nanoclusters in the Ru/TiO_2−*x*_ sample. The Ru–Ru vibration peak at approximately 2.45 Å was obvious for Ru/TiO_2−*x*_, demonstrating that most of the Ru in the catalysts was Ru–Ru coordinated. The wavelet-transform (WT) EXAFS analysis (Fig. 3f) also indicated that Ru/TiO_2−*x*_ exhibited a single intensity maximum corresponding to Ru–Ru coordination. The migration of H_s_ from the catalyst surface into the interior faces a high energy barrier, making inward diffusion unlikely. Consequently, hydrogen spillover in the Ru/TiO_2−*x*_ catalyst is expected to occur primarily at interfacial regions, where Ru nanoclusters contact the TiO_2−*x*_ support and provide energetically favorable migration pathways (Fig. 3g).

### Potential applications of quantitative hydrogen spillover

**Fig. 4:**
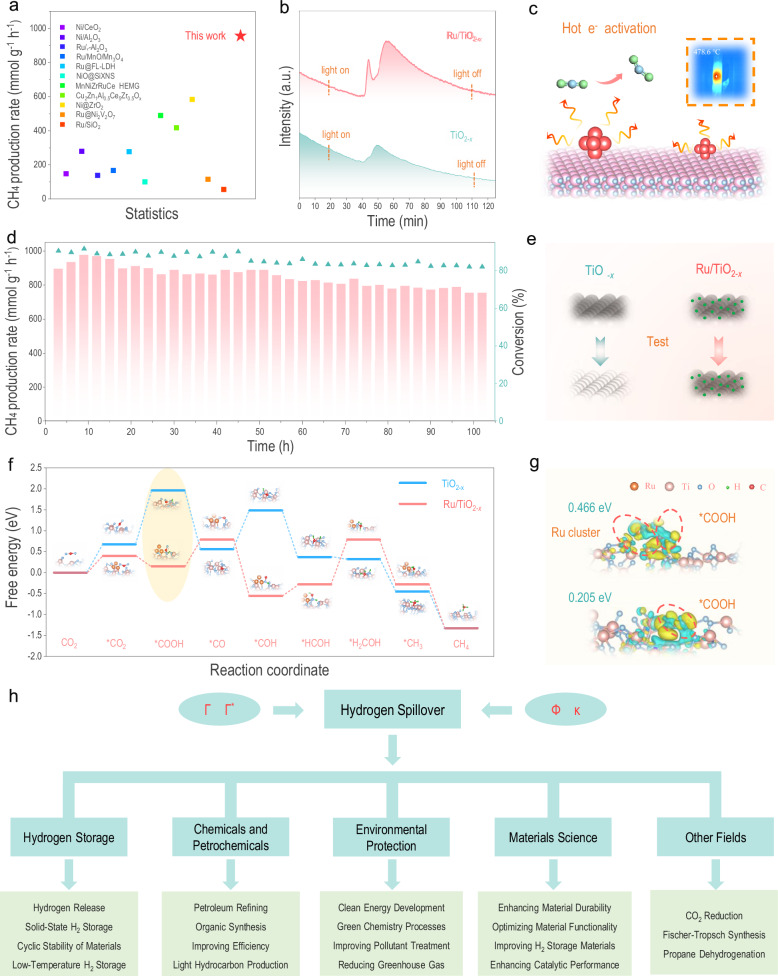
Quantitative promotion of the hydrogen spillover effect. **a** A summary comparing the performance of representative catalysts for light-driven and externally heated Sabatier reactions. **b** Photoexcited desorption test of CO_2_ over Ru/TiO_2−*x*_ and TiO_2−*x*_ samples**. c** Schematics representing the hot electron activation. **d** Quantitative hydrogen spillover to promote the catalyst stability of Ru/TiO_2−*x*_. **e** Color evolution associated with defect-induced electronic structural changes in TiO_2−*x*_ and Ru/TiO_2−*x*_ before and after the test. **f** Free-energy diagrams of the C_1_ pathway for the Ru/TiO_2−*x*_ and TiO_2−*x*_ samples. **g** Charge density of *COOH in the Ru/TiO_2−*x*_ and TiO_2−*x*_ models. **h** The application prospects of quantitative hydrogen spillover effect.

The intricate dynamics of hydrogen spillover are poised to transform key fields, such as hydrogenation reactions and high-capacity chemical hydrogen storage by enabling unprecedented efficiency and scalability. However, the lack of a clear quantitative descriptor has hindered the systematic identification and optimization of effective hybrid solids, creating a key gap in designing next-generation materials. We used the developed quantitative platform to systematically screen well-defined Ru nanocluster modified TiO_2−*x*_, thereby optimizing its catalytic efficiency in hydrogenation reactions and broadening its applicability to high-capacity chemical hydrogen storage. It achieves a record CH_4_ production rate of 948 mmol g^−1^ h^−1^ at a flow rate of 30 mL min^−1^ under ambient pressure, demonstrating a remarkable improvement in light-driven Sabatier reactions (Fig. 4a and Supplementary Table 2).

Using spectroscopy combined with theoretical calculations, we further elucidated the hydrogen spillover mechanism in the CO_2_ hydrogenation reaction over Ru/TiO_2−*x*_. CO_2_-TPD measurements showed that the Ru/TiO_2−*x*_ surface possesses a higher density of CO_2_ adsorption sites (Supplementary Fig. 15). Based on Kissinger plot analysis, the CO_2_ adsorption energy for Ru/TiO_2−*x*_ was calculated to be 3.123 kJ/mol (Supplementary Fig. 16), which is consistent with the higher charge density transferred from Ru/TiO_2−*x*_ to CO_2_ (Supplementary Fig. 17).

In addition, the photoexcited CO_2_ desorption test directly revealed the catalyst’s CO_2_ activation ability, with Ru/TiO_2−*x*_ exhibiting significantly more photoactivated sites than TiO_2−*x*_, confirming its superior activity (Fig. 4b)^22^. Using molecular orbital theory, we elucidated that once adsorption equilibrium is reached on the catalyst surface, bonding (occupied) and antibonding (unoccupied) orbitals are formed. Under light excitation, hot electrons are generated and populate the antibonding orbitals of the adsorbed CO_2_ molecules in the metal–semiconductor interface (Fig. 4c). This electronic injection weakens the intramolecular C = O bonds while strengthening the interaction between CO_2_ and the catalyst, thereby facilitating molecular dissociation and activation. Photoexcited reduction and photoexcited desorption techniques were applied to quantify H_2_ spillover and to count CO_2_ photoactive sites, respectively, thereby providing deeper insight into the in-situ mechanism of CO_2_ hydrogenation.

Ru/TiO_2−*x*_ exhibited exceptional stability over 100 h, highlighting its potential as a robust platform for scalable hydrogenation applications (Fig. 4d, Supplementary Figs. 18–20). In Supplementary Fig. 21, ^13^C isotope labeling experiment has shown that a dominant peak at m/z = 17 corresponding to ^13^CH_4_ and a weaker peak at m/z = 29 corresponding to ^13^CO were detected, confirming that the generated CO and CH_4_ result from the light-driven CO_2_ reduction. The persistent black coloration of Ru/TiO_2−*x*_ after hydrogenation testing is mainly due to the sustained stabilization of oxygen vacancies and Ti^3+^ species that create mid-gap defect states responsible for visible-light absorption. Ru facilitates hydrogen spillover, continuously supplying electrons to counteract reoxidation and vacancy healing, thereby preserving the reduced electronic structure and the associated black appearance (Fig. 4e). Moreover, UV‒vis‒NIR absorption tests (Supplementary Fig. 22), projected density of states (PDOS) calculations (Supplementary Fig. 23), along with electron paramagnetic resonance (EPR, Supplementary Fig. 24) and X-ray photoelectron spectroscopy (XPS, Supplementary Figs. 25 and 26) results confirmed that the integrity of TiO_2_ crystals, upon the loss of lattice oxygen, is markedly augmented through the strategic reinforcement of Ru clusters, thereby increasing their durability and mitigating deactivation processes. On the other hand, this indicates that the solid material has the potential for high-capacity hydrogen storage once the conditions are optimized. In other words, the hydrogenation and storage mechanisms may operate synergistically within the same material (Supplementary Fig. 27).

The adsorption of CO_2_ on the Ru/TiO_2−*x*_ surface requires less energy than adsorption on the TiO_2−*x*_ surface does, suggesting that the presence of Ru clusters enhances the adsorption and activation of CO_2_^23–26^. The *COOH intermediate generation from *CO_2_ on the TiO_2−*x*_ surface requires 3.18 eV, whereas it releases 1.23 eV on Ru/TiO_2−*x*_ (Fig. 4f). Moreover, compared with the TiO_2−*x*_ model, greater charge transfer is observed from the Ru/TiO_2−*x*_ model to the *COOH intermediate when it interacts with the surface (Fig. 4g). These results demonstrate that the activation of *COOH intermediates is more favorable on the surface of the Ru/TiO_2−*x*_ model^27,28^. The results of in situ diffuse reflectance infrared Fourier transform spectroscopy further indicate that Ru/TiO_2−*x*_ exhibits a stronger ability to activate CO_2_ during hydrogenation (detailed discussion is given in Supplementary Figs. 28 and 29). Moreover, we would like to highlight the potential of the hydrogen spillover quantification descriptors (Fig. 4h). Beyond the discussion above, these quantitative techniques could be applied to broader research areas, such as aiding the design of light-driven catalysts for industrially important propane dehydrogenation and improving understanding of hydrogen utilization in environmental protection and human therapy.

## Discussion

We decoupled the activation and spillover zones through quantitative thermodynamic‒kinetic descriptors with great repeatability. This quantitative relationship is demonstrated on a variety of catalysts, facilitating a robust description of the hydrogen spillover process. Theoretical calculations and molecular dynamics reveal that the nonlinear hydrogen spillover of *d*-block metals arises from their intrinsic *d–s* orbitals. Partially vacant *d–s* orbitals overlap with the σ and σ* orbitals of adsorbed hydrogen, forming weak antibonding states that partially weaken H–H and M–H bonds. This achievement lays a theoretical foundation and provides a research methodology for in-depth studies of industrial applications centered on hydrogen spillover, showing significant potential in the fields of hydrogenation, storage and petrochemicals.

## Methods

### Preparation of catalyst

In the experiments, TiO_2_ (P25), RuCl_3_·6H_2_O (99.999%), Ni(NO_3_)_2_·9H_2_O (99.999%), Fe(NO_3_)_3_·9H_2_O (99.999%), Co(NO_3_)_2_·6H_2_O (99.999%), Mn(NO_3_)_2_·4H_2_O (99.999%) and NaBH_4_ were purchased from Aladdin Co. Ltd., China. A mixture of 1 g of commercial TiO_2_ and 0.5 g of NaBH_4_ was calcined at 350 °C in an argon atmosphere for 2 h. Next, the sample was washed with deionized water three times and then dried to obtain TiO_2−*x*_ catalysts. Thermal treatment of TiO_2_ with NaBH_4_ generates active hydrogen species that extract lattice oxygen and partially reduce Ti^4+^ to Ti^3+^, creating oxygen vacancies and a defect-rich structure that tailors the electronic band and enhances visible-light absorption. To prepare Ru/TiO_2−*x*_, we used an impregnation method with aqueous RuCl_3_·6H_2_O solution to load Ru onto the surface of TiO_2_. After drying at 80 °C for 10 h, the Ru/TiO_2_ precursor was obtained. Subsequently, a mixture of 1 g of the Ru/TiO_2_ precursor and 0.5 g of NaBH_4_ was calcined at 350 °C for 2 h under an argon atmosphere. The resulting product was then washed three times with deionized water and dried to yield the Ru/TiO_2−*x*_ catalyst. In addition, the Ni/TiO_2−*x*,_ Fe/TiO_2−*x*,_ Co/TiO_2−*x*_ and Mn/TiO_2−*x*_ catalysts were prepared via a similar method as the Ru/TiO_2−*x*_ catalyst.

### Characterization

High-angle annular dark-field scanning transmission electron microscopy (HAADF-STEM) images were acquired on a Titan Cubed Themis G2 300. X-ray diffraction (XRD) patterns of the samples were recorded using a Shimadzu LabX-XRD-6000 diffractometer with Cu Kα radiation (40 kV, 40 mA). X-ray photoelectron spectroscopy (XPS, UIVAC-PHI, Japan) was employed to measure the binding energy and valence band spectra. UV–visible (UV–vis) absorption spectra were obtained using a Shimadzu UV-2550 spectrophotometer (Japan). X-ray absorption spectra (XAS), including XANES and EXAFS at the Ru K-edge, were collected at beamline 14 W of the Shanghai Synchrotron Radiation Facility (China) for analysis. Electron paramagnetic resonance (EPR) spectroscopy (Bruker EMX PLUS) was used to analyze the defect content of the samples. The temperature‒programmed CO_2_ desorption (CO_2_-TPD) of the samples was tested with a TPD apparatus. The samples were first purged in a stream of argon at 150 °C for 2 h and then cooled to 25 °C. The saturated adsorption of CO_2_ on the catalyst was subsequently completed at 50 °C for 50 min, after which the physiosorbed molecules were swept away with argon. Finally, the catalysts were uniformly heated at 10 °C min^−1^ in an argon gas stream.

### In situ diffuse reflectance infrared Fourier transform spectroscopy (DRIFTs)

The DRIFTs (Nicolet iS50) were used to analyze the intermediates present during the catalytic reaction. First, the catalysts were fully purged in an argon stream. Afterward, the samples were fully exposed to a mixed gas (H_2_ / CO_2_ = 4: 1) flow of 15 mL min^−1^ for 1 h in the dark and then maintained under illumination with a 300 W Xenon lamp (CEL-PF300-T8, CEAuLight Co., Ltd., China) for 1 h.

### Photoexcited reduction (PER) test

To study the hydrogen spillover process under in situ photoexcitation, we designed a photoexcited analyzer. Prior to formal testing, the catalyst was pretreated at 200 °C in an Ar atmosphere for 30 min to ensure complete removal of CO_2_ and H_2_O. Then, H_2_/Ar was used to blow over the surface of the sample to be tested at a certain flow rate for one hour. After that, the experiments employed a 300 W Xenon lamp (CEL-PF300-T8, CEAuLight Co., Ltd., China) as the light source, with an irradiation intensity of approximately 1 W cm^−2^. The illumination intensity was controlled by adjusting the driving current of the light source, and the resulting light intensity were measured using a Thorlabs PM100D optical power meter with an S425C sensor. A TCD detector was used for quantitative detection and analysis. In the PER test, a high-purity H_2_/Ar mixed gas was introduced, the observed peak changes corresponded exclusively to variations in H_2_, and no peaks were detected under a pure Ar atmosphere.

### Photoexcited desorption test

A photoexcitation analyzer previously developed by our group was employed to examine the CO_2_ desorption behavior of the sample^22^. The sample was first saturated with CO_2_ and then purged with a carrier gas to remove weakly adsorbed species. Subsequently, the light source was switched on to cleave the bonds of chemisorbed species, inducing desorption of adsorbates with different binding strengths. The desorbed gases were then analyzed by the detector.

### Catalytic performance tests

The catalytic performance was evaluated under ambient pressure. In a typical experiment, 50 mg of catalyst was placed at the center of a tubular reactor, and a thermocouple was inserted for precise temperature control. A 300 W Xenon lamp (CET-PF300-T8, CEAuLight Co., Ltd., China) with a full spectrum irradiation intensity of 1 W cm^−2^ served as the light source. A mixture of H_2_ and CO_2_ (30 mL min^−1^, 1.0 bar) was first introduced to purge air from the system, after which a H_2_/ CO_2_ feed with a molar ratio of 4: 1 was supplied. The individual H_2_ and CO_2_ flow rates were regulated by mass flow controllers. Reaction products were analyzed online using gas chromatography (Agilent 8890).

### Computational details

We have employed the Vienna Ab initio Simulation Package (VASP 5.2) to perform all density functional theory (DFT) calculations. The elemental core and valence electrons were represented by the projector augmented wave method and plane-wave basis functions with a cutoff energy of 400 eV. Generalized gradient approximation with the Perdew–Burke–Ernzerh of (GGA-PBE) exchange-correlation functional was employed in all the calculations. Geometry optimizations were performed with the force convergency smaller than 0.05 eV/Å. The spin-polarization effect was also considered. A climbing image nudged elastic band method was used to locate the transition states with the same convergence standard. The spin-polarization effect was also considered. The DFT-D3 empirical correction method was employed to describe van der Waals interactions.

### Ab initio molecular dynamics (AIMD) simulation

The work uses AIMD to simulate a system for 720 femtoseconds (fs) at 750 Kelvin (K) using a canonical ensemble, integrating equations of motion with a 1.0 fs time step. Periodic boundary conditions are applied in the *x* and *y* planes with a 15 Angstrom (Å) vacuum layer along the *z*-axis to isolate the system. The bottom layer of the simulated slab is fixed to mimic bulk constraints, allowing the rest of the atoms to move and relax.

## Supplementary information

Supplementary Information

Transparent Peer Review file

## Data Availability

All data supporting the findings of this study are available within the article and its Supplementary Information. And all data are available from the corresponding author upon request.
