# Supplementary material for: Origins of the hydrogen spillover effect in d-block metals
Source: Nat Commun. 2026 May 11;17:6317. doi: 10.1038/s41467-026-72608-0 (PMC13376162; doi:10.1038/s41467-026-72608-0)
Supplement: Supplementary file 1 — Supplementary Information [file 41467_2026_72608_MOESM1_ESM.pdf]

Supplementary Information for

Origins of the hydrogen spillover effect in *d*-block metals

Yang Li[1, 2, †], Yuanming Zhang[1, 2, †], Zhaojian Zeng[1, 2 †], Yong Chen[1, 2], Xiaoming Xu[1, 2], Zhigang Zou[1, 2], Zhaosheng Li[1, 2, *]

[1]Collaborative Innovation Center of Advanced Microstructures, National Laboratory of Solid State Microstructures, College of Engineering and Applied Sciences, Nanjing University, 22 Hankou Road, Nanjing 210093, China

[2]Jiangsu Key Laboratory of Nano Technology, Nanjing University, 22 Hankou Road, Nanjing 210093, China

*Correspondence to: zsli@nju.edu.cn

[†]These authors contributed equally to this work.

Supplementary Note | The computational details are explained by taking 1.00%-Ru/TiO_{2-x} as a representative example.

The spillover degree (Γ) was calculated according to Equation (1). Here, a represents the normalization factor, and I denotes the intensity obtained from the photoexcited reduction test. By integrating the curve over the time interval from t_0 to t_1, the value of $a\int_{t_0}^{t_1} Idt$ was determined to be 0.0369 mmol. The mass of the catalyst (m_{cat}) was calculated to be 50 mg. Accordingly, the spillover degree Γ was calculated to be 0.738 mmol g^{-1}_{cat}.

$$\Gamma = \frac{a\int_{t_0}^{t_1} Idt}{m_{cat}} \qquad (1)$$

The characteristic spillover degree (Γ*) was calculated using Equation (2). Analogous to the calculation of Γ, m_{cat} was replaced by the mass of metal (m_{metal})). The value of m_{metal} was calculated to be 0.5 mg, yielding a Γ* value of 73.8 mmol g^{-1}_{metal}.

$$\Gamma^* = \frac{a\int_{t_0}^{t_1} Idt}{m_{metal}} \qquad (2)$$

The activity parameter (Φ) was evaluated based on Equation (3). This value was obtained by calculating the absolute time derivative of the intensity over t_0–t_1 and taking its maximum. As a result, Φ was determined to be 0.301 i s^{-1}.

$$\Phi = max\left\{\frac{dI(t)}{dt}\right\}, t \in [t_0, t_1] \qquad (3)$$

The average spillover rate (κ) was calculated according to Equation (4). By integrating the curve over the time interval from t_1 to t_2, the value of $a\int_{t_1}^{t_2} Idt$ was determined to be 2721.20 mol g^{-1}_{cat}. The corresponding duration ($t_2 - t_1$) was calculated to be 346.65 min, leading to an average spillover rate κ of 7.85 mol g^{-1}_{cat} min^{-1}.

$$\kappa = \frac{a\int_{t_1}^{t_2} Idt}{t_2 - t_1} \qquad (4)$$

As shown in **Supplementary Table 1**, the numerical values of the descriptors for different samples, derived using this calculation method and illustrated in **Fig. 1**, are summarized below.

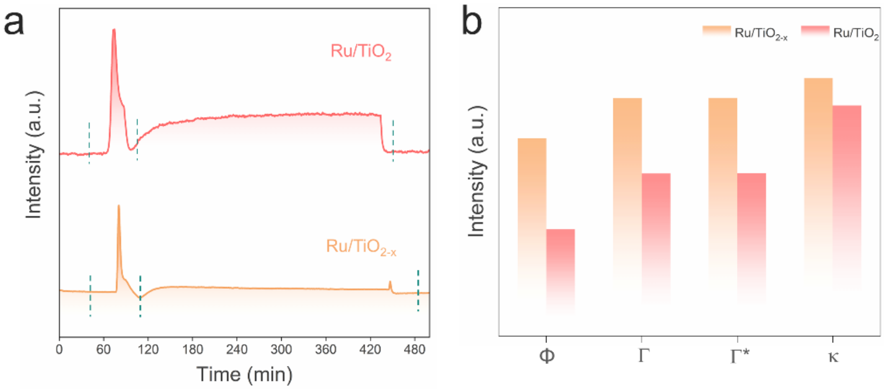

Supplementary Fig. 1 | Effect of support type. **a**, Photoexcited reduction tests for Ru/TiO_2 and Ru/TiO_{2-x}. **b,** Comparison of descriptors (Γ, Γ*, Φ, and κ) for Ru/TiO_2 and Ru/TiO_{2-x}.

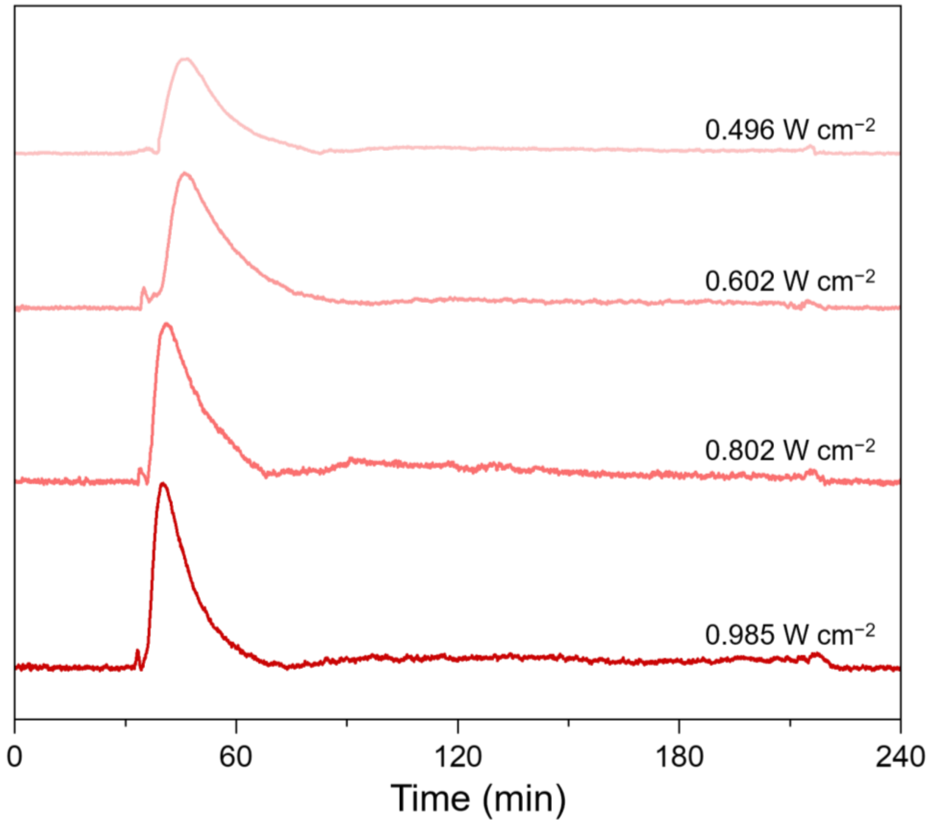

Supplementary Fig. 2 | Effect of light intensity. Photoexcited reduction test curves obtained for Ru/TiO_{2-x} under varying light intensities.

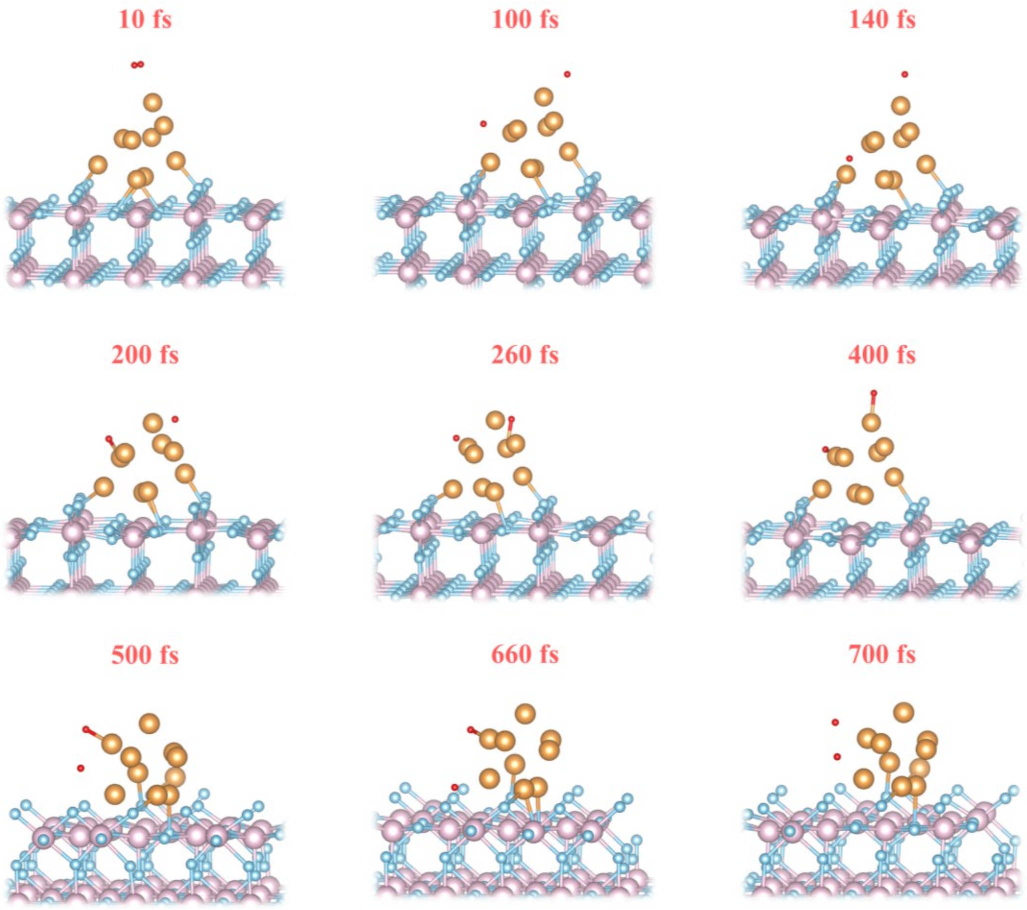

Supplementary Fig. 3 | Molecular dynamics simulation. Molecular dynamics simulation visualizing the process of hydrogen spillover.

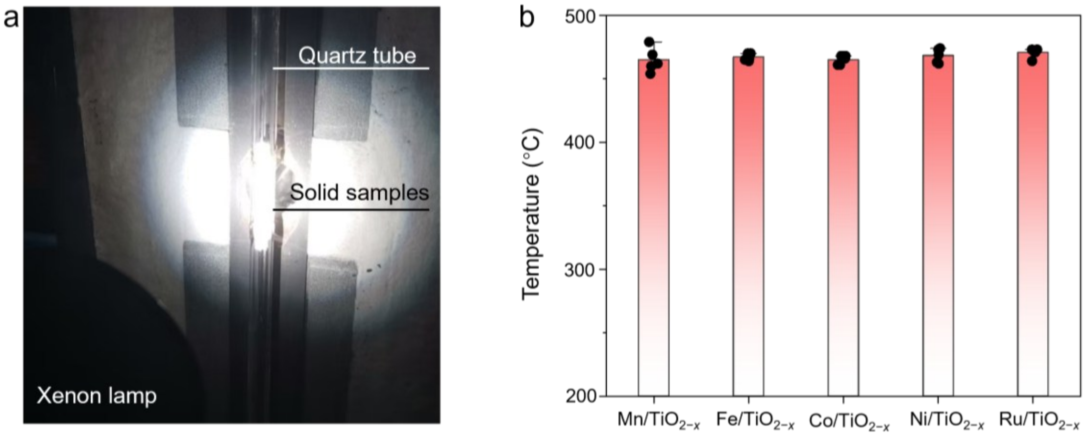

Supplementary Fig. 4 | Real-time temperature monitoring for PER testing. a, Optical image of the local structure in the PER test. **b**, Average temperature distribution from an infrared (IR) camera under 300 W Xe lamp irradiation in the PER test. The IR images of the sample surface in the reactor were captured by an IR camera (FLUKE, TiX580) with the corresponding emissivity and were analysed by software (SmartView).

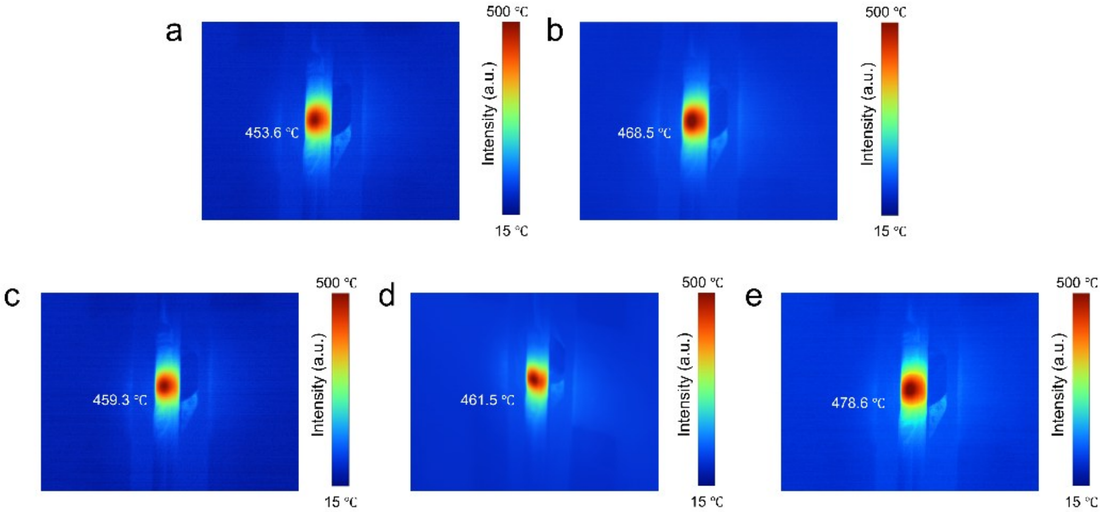

Supplementary Fig. 5 | IR images of the solid catalyst recorded after the same-duration 300 W Xe-lamp irradiation. **a**, Mn/TiO_{2-x}; **b**, Fe/TiO_{2-x}; **c**, Co/TiO_{2-x}; **d**, Ni/TiO_{2-x}; **e**, Ru/TiO_{2-x}.

As shown in Supplementary Fig. 4a, in our PER experiments using a 300 W Xenon lamp (CEL-PF300-T8, CEAuLight Co., Ltd., China) with an irradiation intensity of approximately 1 W/cm^2, the solid catalyst was confined within a thin quartz tube, and the metal loading was controlled below 1%. Owing to the excellent photothermal conversion capability of the black TiO_{2-x} support, a significant surface temperature rise was observed under illumination. Although a small amount of metal loading can further influence the localized photothermal effect, the overall surface temperatures of different metal/TiO_{2-x} systems remained in a narrow range of 466–468 °C (Supplementary Fig. 4b and Supplementary Fig. 5). This relatively small temperature variation is insufficient to account for the observed hydrogen activation, underscoring that the hot electron mechanism originated from different metals, rather than simple thermal heating, plays the dominant role in this system.

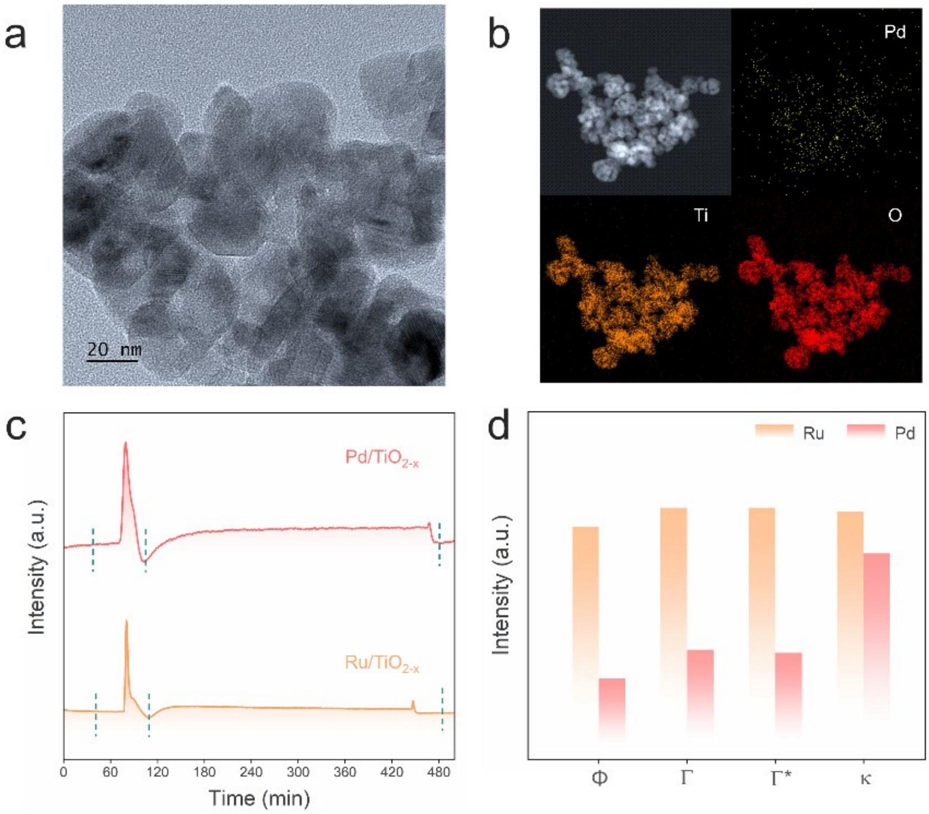

Supplementary Fig. 6 | Structural characterization and evaluation of the photoexcited reduction test of Pd/TiO_{2-x}. **a**, **b**, Transmission electron microscopy (TEM) and Energy dispersive X-ray (EDX) mapping images of Pd/TiO_{2-x}. **c**, Photoexcited reduction tests for Pd/TiO_{2-x}. **d**, Relative numerical evaluation of descriptors (Γ, Γ*, Φ, and κ) for Pd/TiO_{2-x} and Ru/TiO_{2-x}.

As shown in Supplementary Fig. 6, Pd metal are uniformly dispersed on the TiO_{2-x} support. During the PER test, the processes of hydrogen activation and spillover can be clearly observed. Quantitative analysis reveals that all performance metrics for Pd are lower than those for Ru, indicating that Pd exhibits a weaker capability for hydrogen activation.

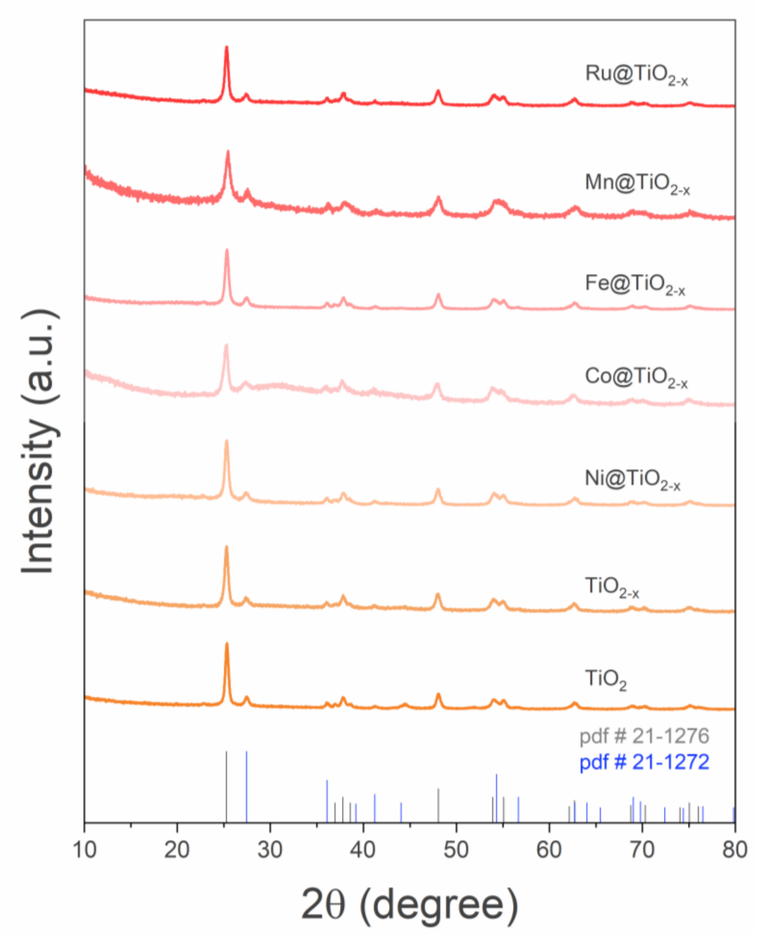

Supplementary Fig. 7 | XRD patterns. XRD patterns of the TiO_2, TiO_{2-x}, Ru/TiO_{2-x}, Mn/TiO_{2-x}, Fe/TiO_{2-x}, Co/TiO_{2-x} and Ni/TiO_{2-x} samples (pdf # 21–1276 represent rutile TiO_2, pdf # 21–1272 represent anatase TiO_2).

As shown in Supplementary Fig. 7, the crystal structure remained unaltered after metal deposition. Based on the XRD results, where the (101) peak is the most intense, the TiO_2 (101) surface was used as the model in our DFT calculations, representing the predominant exposed facet.

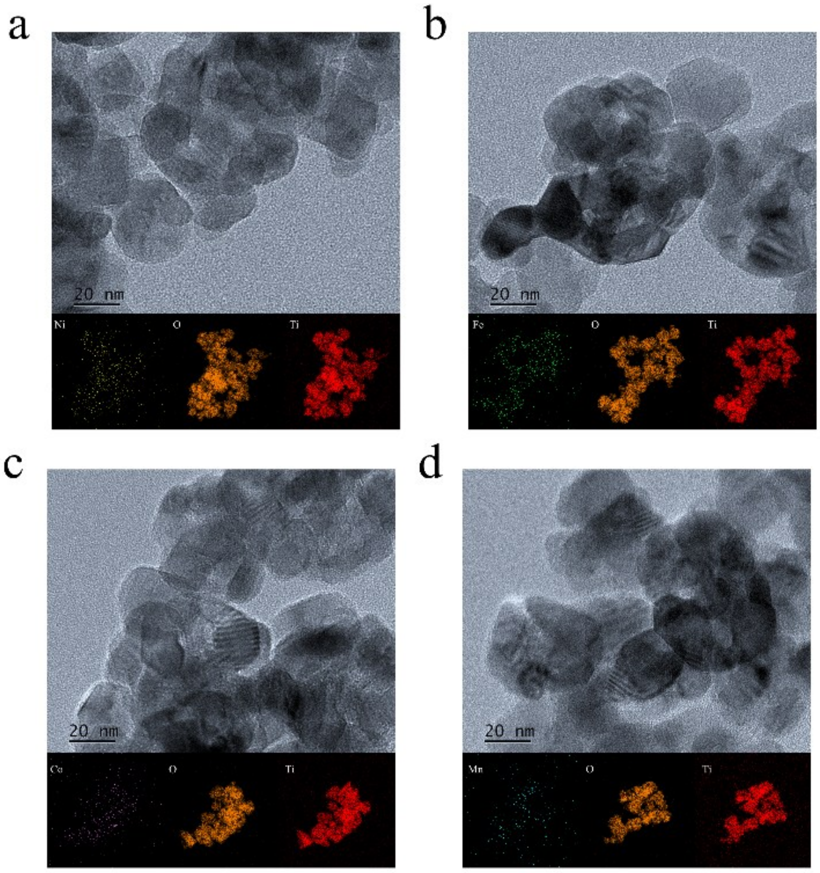

Supplementary Fig. 8 | TEM images and elemental mappings of all samples. **a**, Ni/TiO_{2-x}. **b**, Fe/TiO_{2-x}. **c**, Co/TiO_{2-x}. **d**, Mn/TiO_{2-x}.

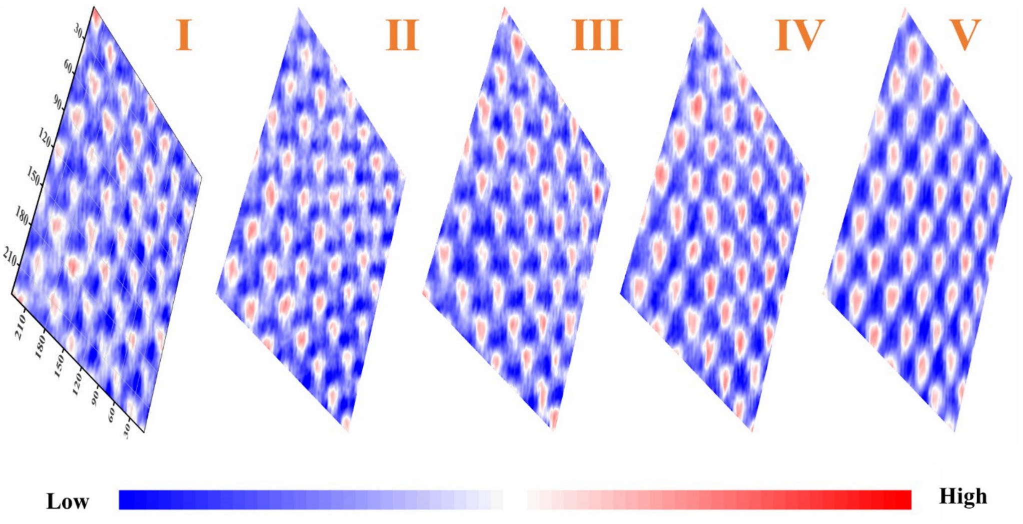

Supplementary Fig. 9 | Two-dimensional intensity surface plots are shown. The plot for the yellow dashed region of the image in **Fig. 3b** is presented.

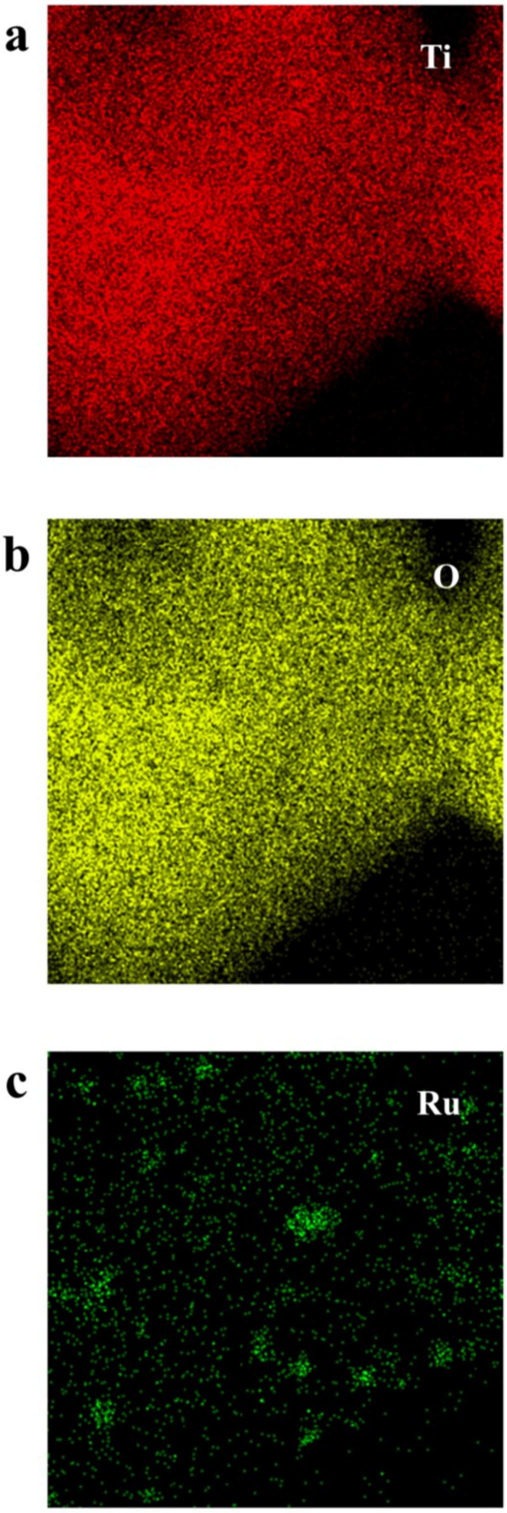

Supplementary Fig. 10 | EDX mapping images of the Ru/TiO_{2-x} sample. **a**, Ti. **b**, O. **c**, Ru.

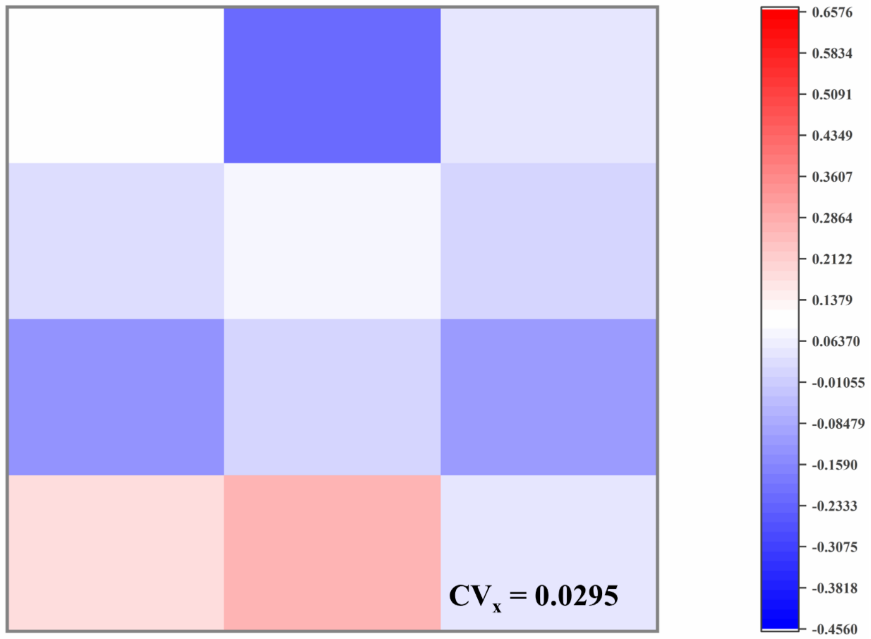

Supplementary Fig. 11 | Discrete atomic distribution maps. Corresponding discrete atomic distribution maps along the *x* direction (coefficients of variation, CV_x) for the red dashed regions in **Fig. 3b**.

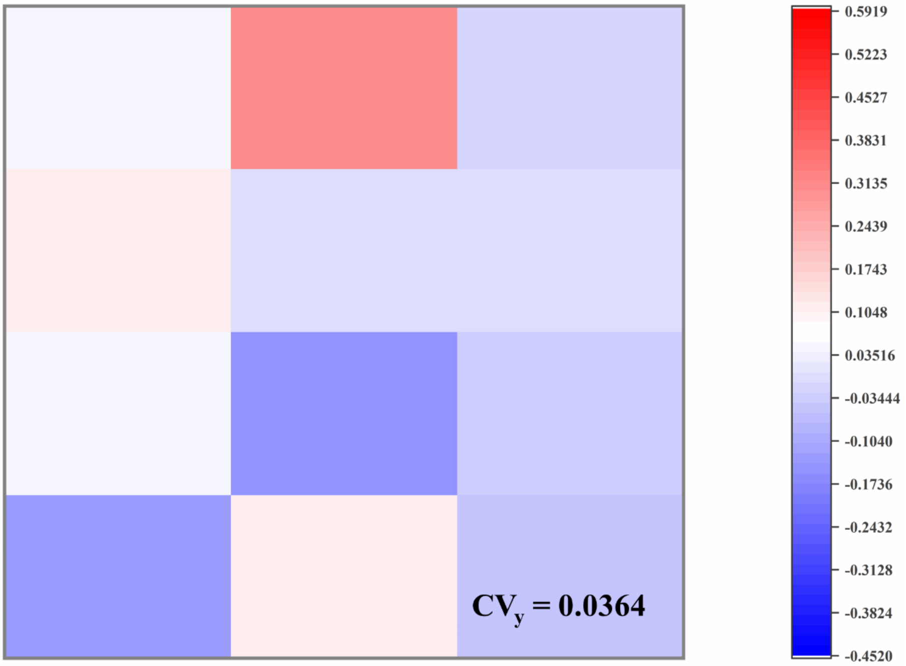

Supplementary Fig. 12 | Discrete atomic distribution maps. Corresponding discrete atomic distribution maps along the *y* direction (coefficients of variation, CV_y) for the red dashed regions in **Fig. 3b**.

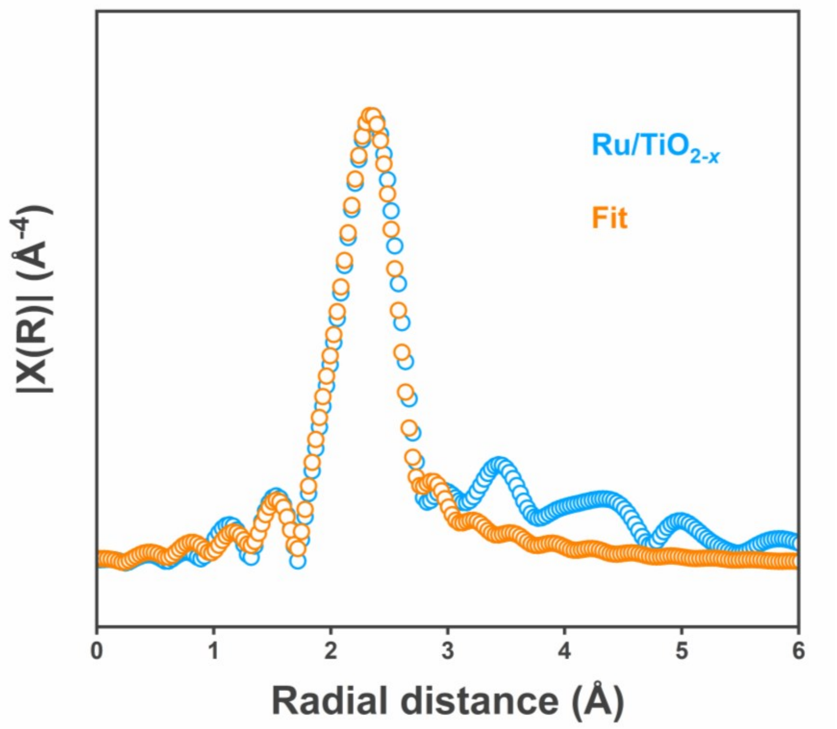

Supplementary Fig. 13 | EXAFS R-space fitting curves are obtained. The curve of Ru/TiO_{2-x} is shown.

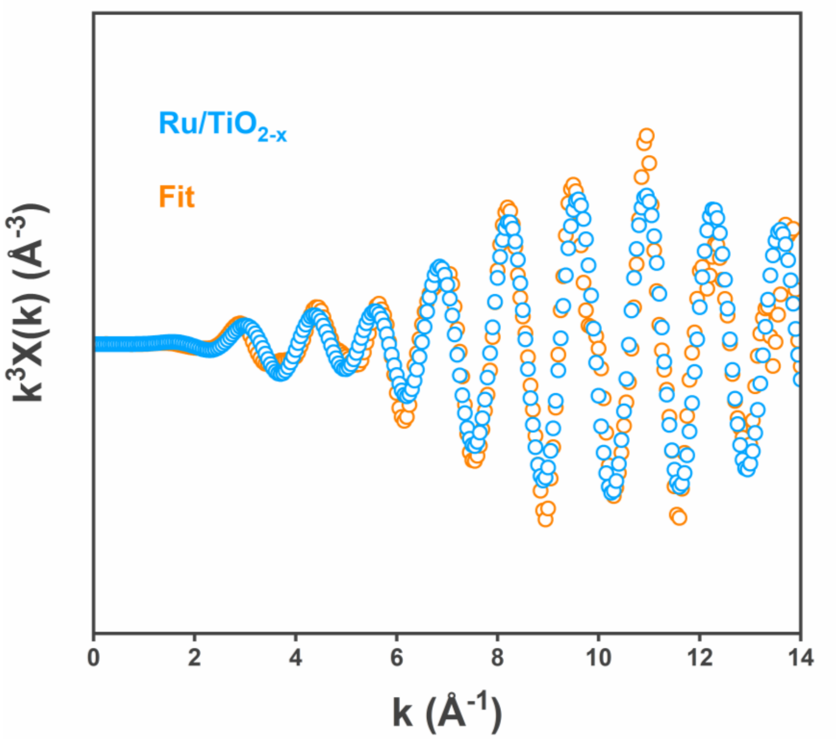

Supplementary Fig. 14 | EXAFS k-space fitting curves are obtained. The curve of Ru/TiO_{2-x} is shown.

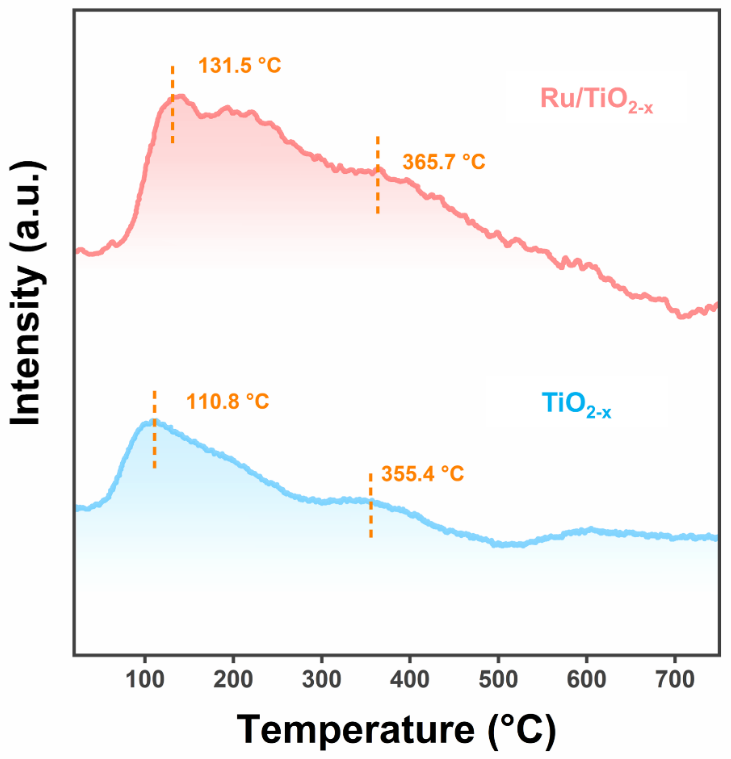

Supplementary Fig. 15 | CO_2-TPD tests are performed. The desorption profiles of Ru/TiO_{2-x} and TiO_{2-x} are compared.

During the CO_2-TPD test for Ru/TiO_{2-x}, desorption peaks of CO_2 are observed throughout the 100–500 °C range, indicating that the CO_2 adsorption capability of Ru/TiO_{2-x} is strong. In contrast, TiO_{2-x} shows a significant desorption peak only at approximately 110 °C, suggesting a weaker CO_2 adsorption capability.

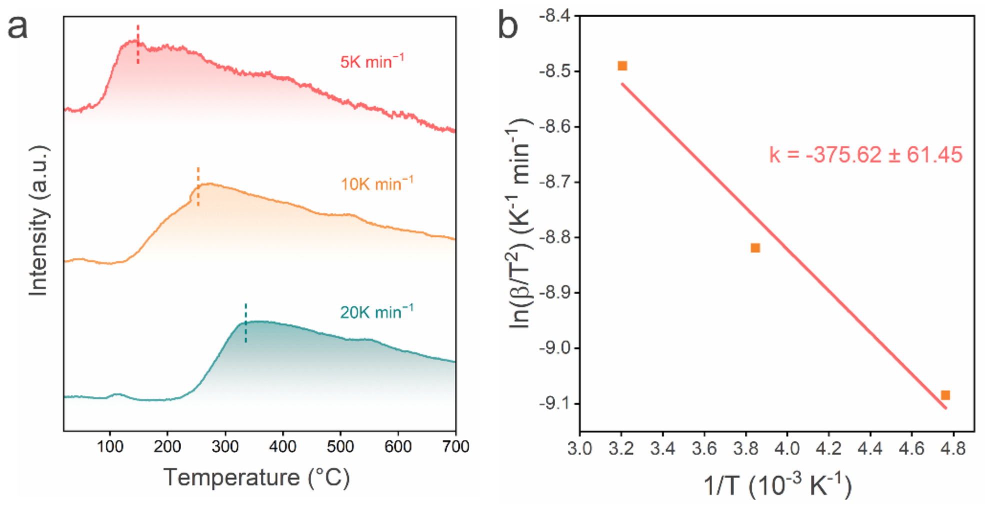

Supplementary Fig. 16 | The apparent activation energy (E_a) for CO_2 desorption was estimated via Kissinger's method. **a**, CO_2-TPD profiles for Ru/TiO_{2-x} at different heating rates. **b**, Calculation of CO_2 desorption energy based on the Kissinger's plots.

As shown in Supplementary Fig. 16, the E_a for CO_2 desorption was estimated using Kissinger's method. According to the Kissinger equation, $\ln\left(\beta/T_p^2\right) = (-E_a/R) * (1/T_p) + \ln\,(AR/E_a)$, where β represents the heating rate, T_p represents the peak temperature, E_a represents the apparent activation energy, R represents the gas constant (8.314 J mol^{-1}·K^{-1}), and A represents the pre-exponential factor. By performing CO_2-TPD experiments at different heating rates and plotting $\ln\left(\beta/T_p^2\right)$ versus $1/T_p$, a linear fit was obtained. The slope of the line $(-E_a/R)$ was used to calculate the apparent activation energy. The CO_2 desorption peak shifts significantly toward higher temperatures with increasing heating rates. On the basis of Kissinger plots, the CO_2 adsorption energy can be calculated as 3.123 kJ/mol.

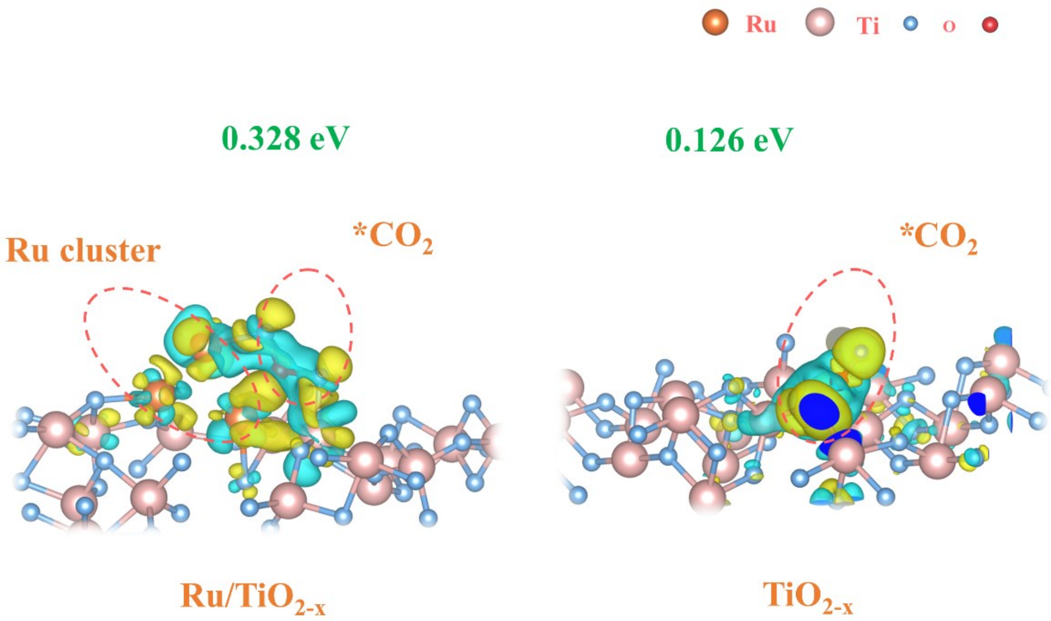

Supplementary Fig. 17 | DFT calculation. Charge density of CO_2 on the Ru/TiO_{2-x} and TiO_{2-x} samples.

When CO_2 was adsorbed onto Ru/TiO_{2-x}, 0.328 eV of charge was transferred from Ru/TiO_{2-x} to CO_2. In contrast, when CO_2 was adsorbed onto TiO_{2-x}, only 0.126 eV of charge was transferred from TiO_{2-x} to CO_2.

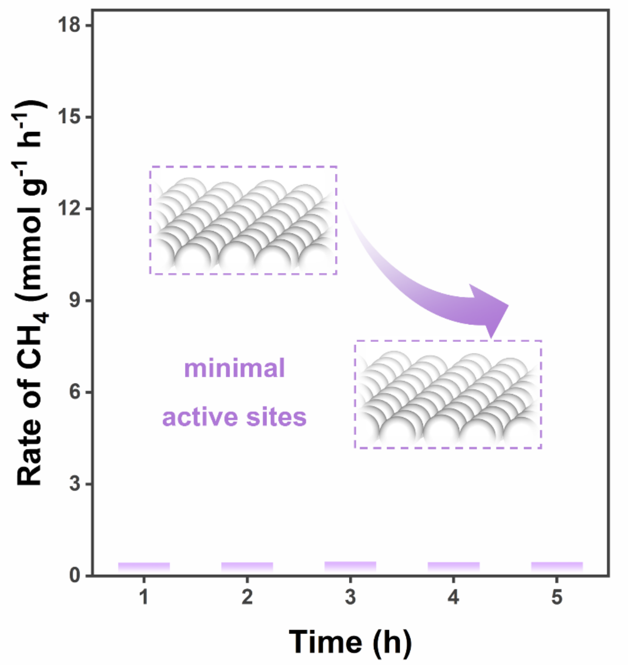

Supplementary Fig. 18 | CH_4 generation performance. Light-driven CO_2 reduction test for the TiO_2 sample.

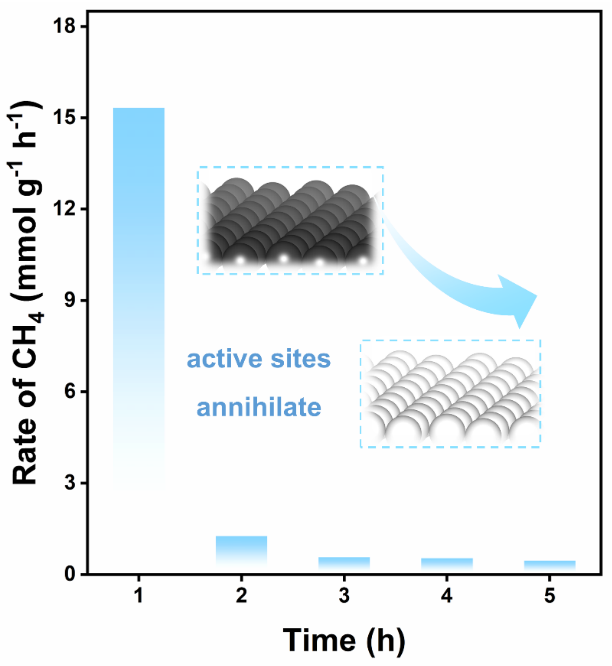

Supplementary Fig. 19 | CH_4 generation performance. Light-driven CO_2 reduction test for the TiO_{2-x} sample.

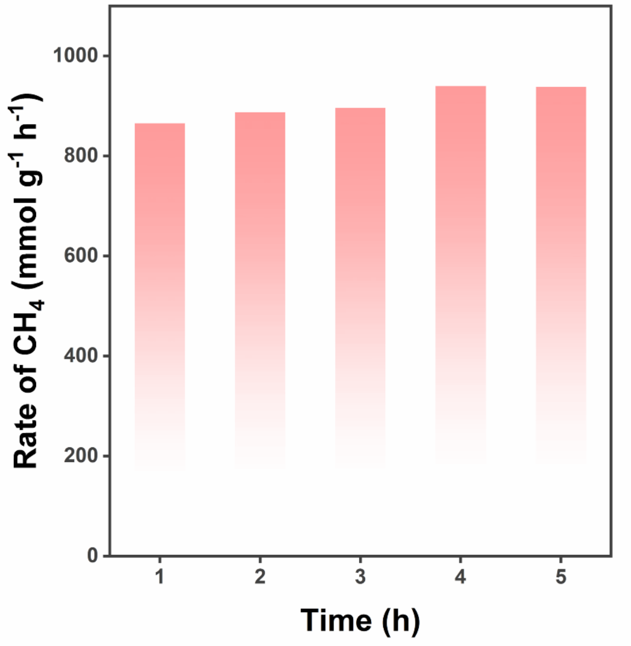

Supplementary Fig. 20 | CH_4 generation performance. Light-driven CO_2 reduction test for the Ru/TiO_{2-x} sample.

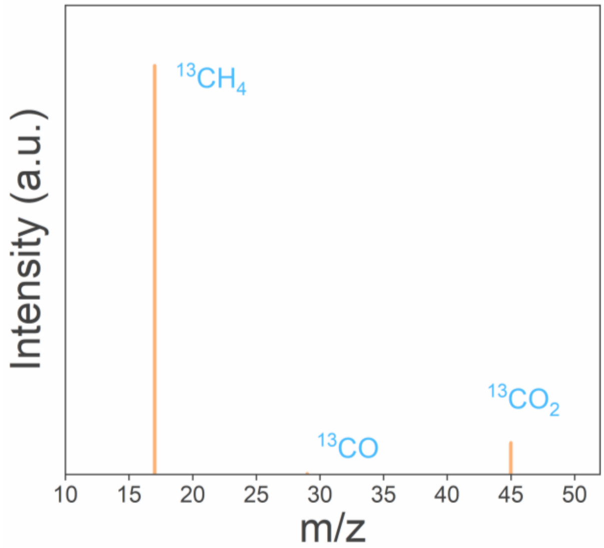

Supplementary Fig. 21 | Isotope experiment. Mass spectrum of the products after $^{13}CO_2$ hydrogenation for Ru/TiO_{2-x}.

As shown in the Supplementary Fig. 21, CH_4 was virtually the only product detected, with significantly low levels of CO, which further confirms the high selectivity toward CH_4 formation.

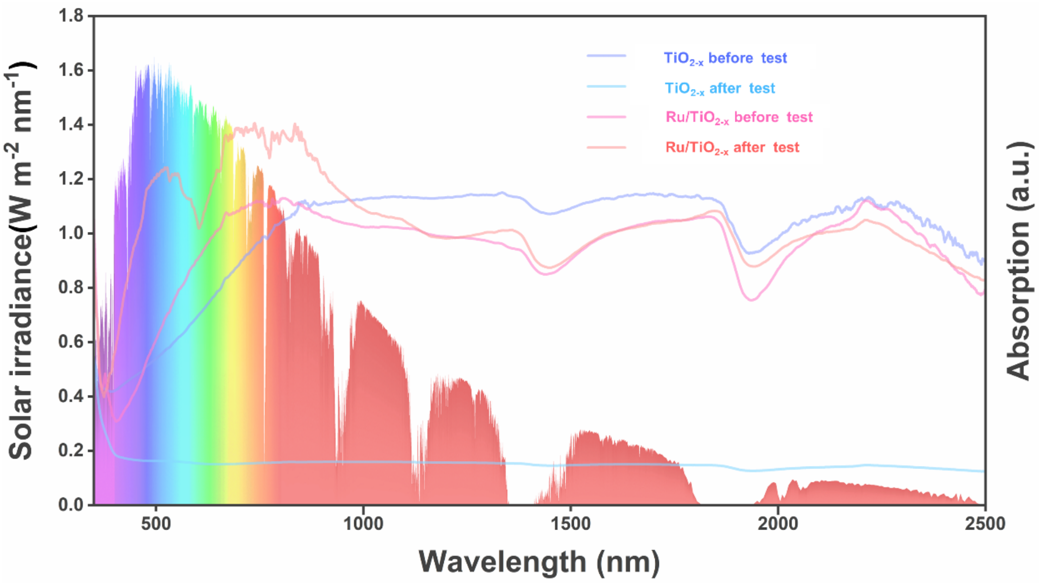

Supplementary Fig. 22 | Comparison of light absorption capability. UV–vis–NIR absorption spectra of before and after the reaction of TiO_{2-x} and Ru/TiO_{2-x}.

TiO_{2-x} exhibits excellent light absorption across the entire spectrum. After conducting light-driven CO_2 reduction tests, it experiences fading and deactivation, significantly reducing its light absorption capacity. However, with the modification of Ru clusters, strong light absorption was retained both before and after the tests.

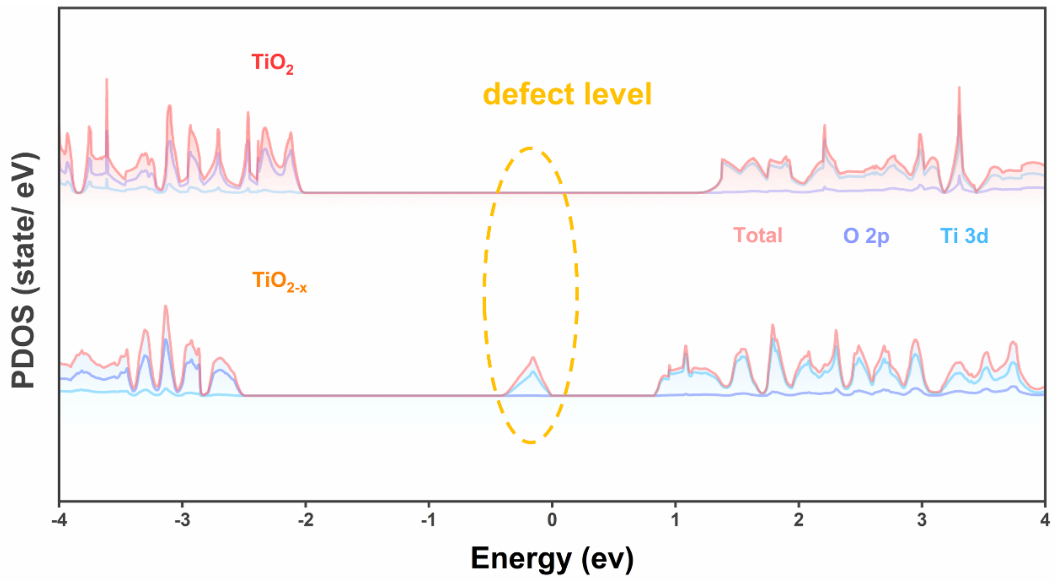

Supplementary Fig. 23 | DFT calculation. Partial density of states (PDOS) of TiO_2 and TiO_{2-x}.

The band gap of TiO_{2-x} is approximately 3.2 eV. After defect engineering, impurity levels are introduced into the band gap, which can affect both the charge transport efficiency and light absorption efficiency.

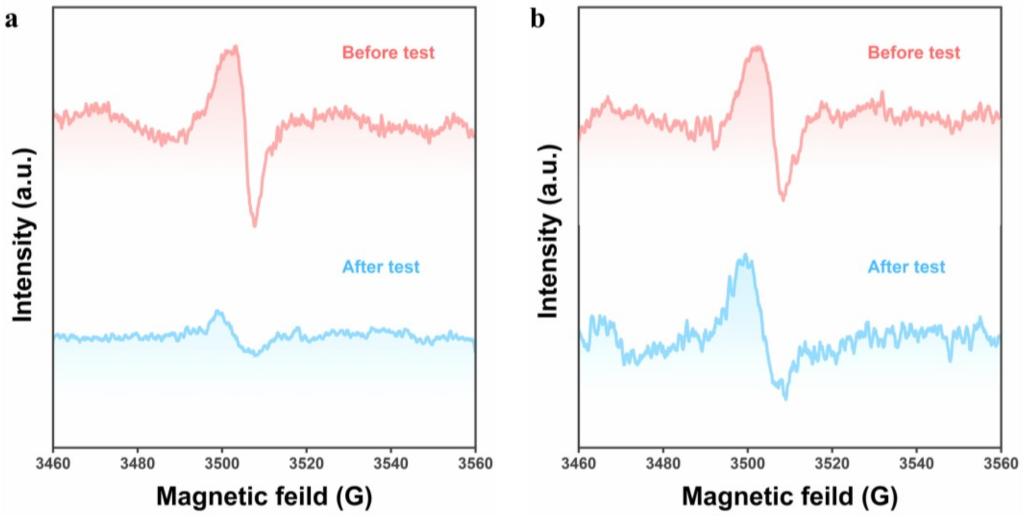

Supplementary Fig. 24 | EPR spectra of the samples before and after light-driven CO_2 reduction. **a**, TiO_{2-x}. **b**, Ru/TiO_{2-x}.

There was a large amount of oxygen defect on the TiO_{2-x} surface before the light-driven CO_2 reaction. However, the quantity of defective species drastically decreased after the reaction. For the Ru/TiO_{2-x} catalyst, the valence states of the oxygen in the catalyst hardly changed before and after the test.

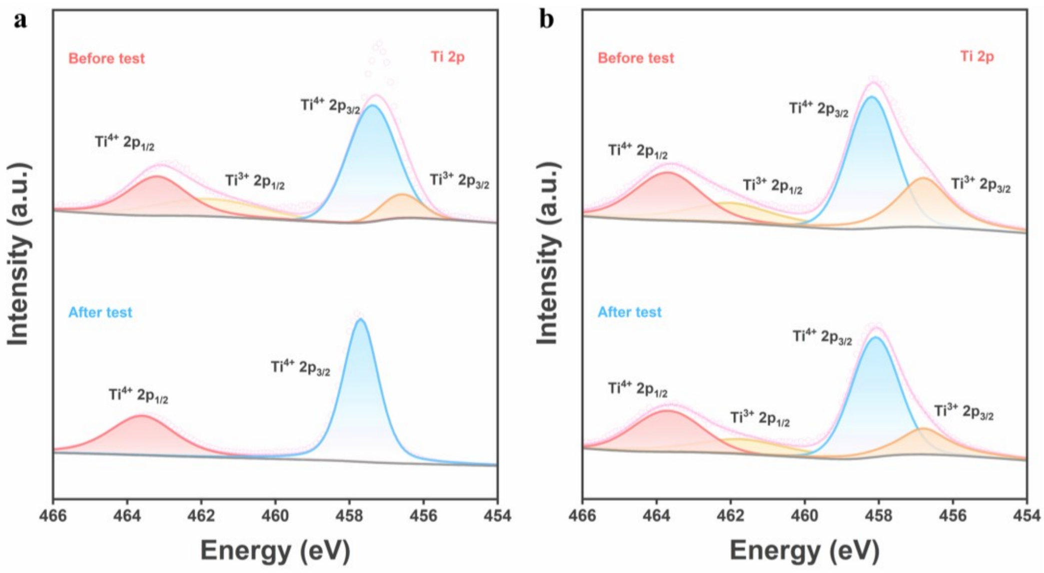

Supplementary Fig. 25 | XPS spectra of the samples before and after light-driven CO_2 reduction. **a**, Ti 2*p* XPS spectra of TiO_{2-x}. **b**, Ti 2*p* XPS spectra of Ru/TiO_{2-x}.

There was a large amount of Ti^{3+} species on the TiO_{2-x} surface before the light-driven CO_2 reduction reaction. However, the quantity of defective species drastically decreased after the reaction. For the Ru/TiO_{2-x} catalyst, the valence state of the elemental titanium in the catalyst hardly changed before and after the test, and there was still a mixture of Ti^{4+} and Ti^{3+} in the catalyst.

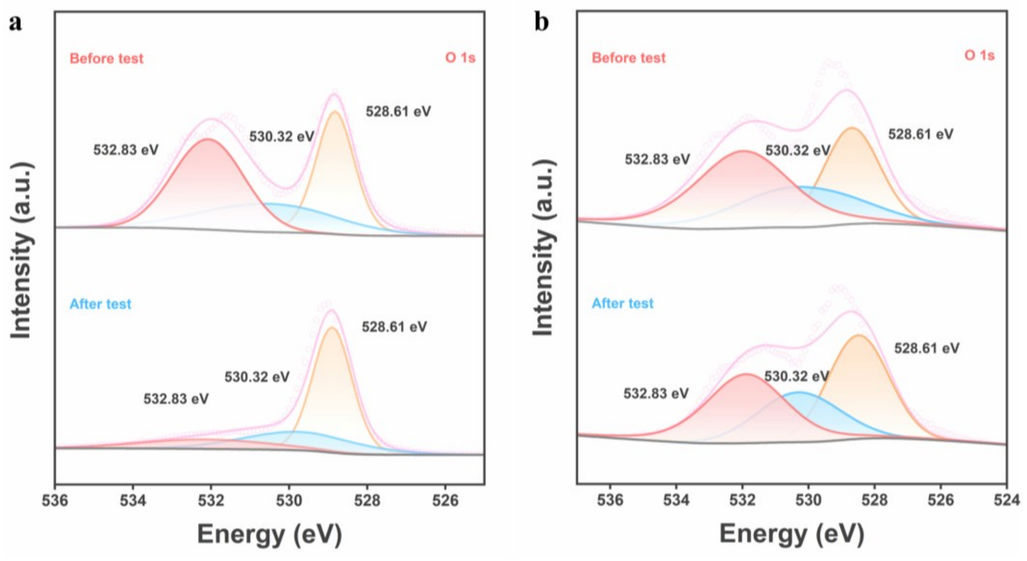

Supplementary Fig. 26 | XPS spectra of the samples before and after the light-driven CO_2 reduction test. **a**, O 1*s* XPS spectra of TiO_{2-x}. **b**, O 1*s* XPS spectra of Ru/TiO_{2-x}.

There was a large amount of oxygen on the TiO_{2-x} surface before the light-driven CO_2 reaction. However, the quantity of defective species drastically decreased after the reaction. For the Ru/TiO_{2-x} catalyst, the valence states of the oxygen in the catalyst hardly changed before and after the test.

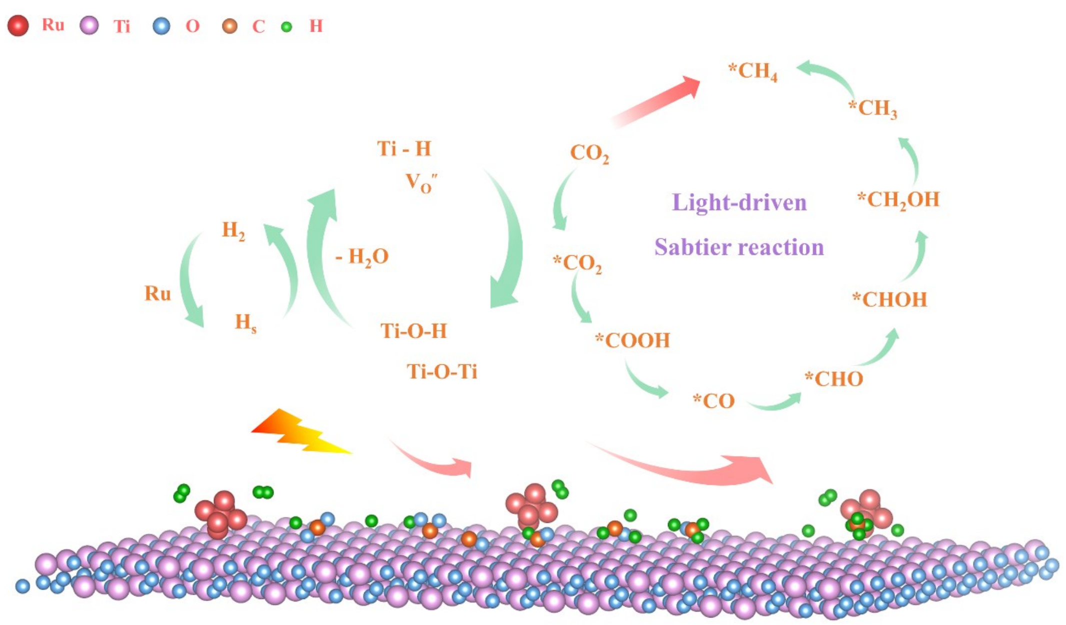

Supplementary Fig. 27 | Analysis of deactivation and stabilization mechanisms. Schematic diagram of the mechanism of deactivation and stabilization over Ru/TiO_{2-x} in light-driven CO_2 reduction reaction.

A large amount of Ti-H on the surface of TiO_{2-x} can capture CO_2, facilitating subsequent reactions. However, the limited ability of TiO_{2-x} to activate H_2 results in the continuous depletion of active sites, ultimately leading to deactivation. The introduction of Ru clusters on the surface of TiO_{2-x} significantly enhances H_2 activation, enabling the rapid conversion of H_2 into H_s, which rapidly migrates to the surface of TiO_{2-x} to protect the support and participate in subsequent reactions.

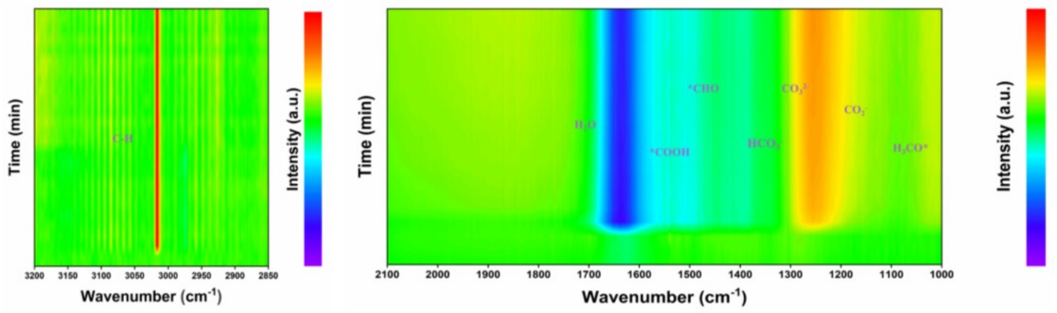

Supplementary Fig. 28 | *In situ* DRIFTs. *In situ* DRIFTs of Ru/TiO_{2-x} under illumination with a 300 W Xe lamp.

There is an obvious stretching vibration of C–H at approximately 3050 cm^{-1}, indicating that an abundance of CH_4 was created throughout the entire reaction. The intermediates CO_3^{2-}, CO_2^-, HCO_3^- and *COOH are mostly represented by signal peaks at 1287, 1223, 1390 and 1600 cm^{-1}, respectively, which are the key intermediates in the production of CH_4. In addition, there is almost no peak for the *CO intermediate at approximately 2050 cm^{-1}, demonstrating the high selectivity of CH_4 throughout the reaction.

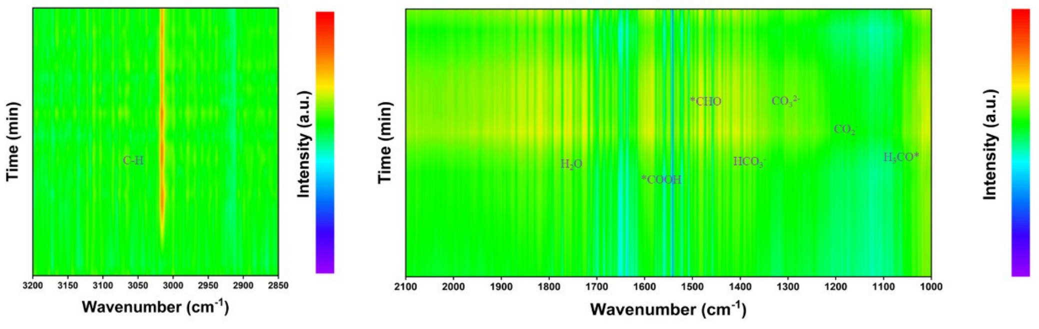

Supplementary Fig. 29 | *In situ* DRIFTs. *In situ* DRIFTs of TiO_{2-x} under illumination with a 300 W Xe lamp.

Supplementary Table 1 | Summary of the numerical values of the descriptors from **Fig. 1b** for different samples.

Sample	Γ (mmol g^{-1}_{cat})	Γ* (mmol g^{-1}_{metal})	Φ (i s^{-1})	κ (mol g^{-1}_{cat} min^{-1})
0.01%-Ru/TiO_{2-x}	0.052	5.2	0.034	2.24
0.05%-Ru/TiO_{2-x}	0.228	22.8	0.062	3.32
0.10%-Ru/TiO_{2-x}	0.288	28.8	0.065	4.89
0.20%-Ru/TiO_{2-x}	0.623	62.3	0.099	8.41
0.40%-Ru/TiO_{2-x}	0.646	64.6	0.192	7.65
1.00%-Ru/TiO_{2-x}	0.738	73.8	0.301	7.85

Supplementary Table 2 | Comparison with representative catalysts for light-driven and external heat-driven Sabatier reactions. The blue background represents hybrid catalysts containing Ni or other metals, while the light orange background represents hybrid catalysts containing Ru.

Catalyst	Reactor	Operation Parameters	CH_4 yield (mmol g^{-1}_{cat} h^{-1})	Stability (h)	References
Ni/CeO_2	flow	0.1 MPa, 225 °C (external), 50 mL min^{-1}, $H_2/CO_2 = 4$	147.1	150	1
Ni/Al_2O_3	flow	0.1 MPa, 290 °C,10 mL min^{-1}, $H_2/CO_2/Ar = 60/15/25$	279	100	2
NiO@SiXNS	flow	0.1 MPa, 300 °C, 5 mL min^{-1}, $H_2/CO_2 = 4$	100	12	3
$Cu_2Zn_1Al_{0.5}Ce_5Zr_{0.5}O_x$	flow	0.1 MPa, 500 °C, 4000 mL min^{-1}, $H_2/CO_2 = 1$	417.2	10	4
Ni/ZrO_2	flow	0.1 MPa, 225 °C, 50 mL min^{-1}, $H_2/CO_2 = 4$	583.3	10	5
Ru/γ-Al_2O_3	flow	0.1 MPa, 350 °C (external), 41.6 mL min^{-1}, 15.5% CO_2, 80.9% H_2	137.88	15	6
$Ru/MnO/Mn_3O_4$	batch	180mL, 1MPa, $H_2/CO_2 = 4$	166.7	5	7
Ru@FL-LDH	flow	0.1 MPa, 350 °C, 125.0 mL min^{-1}, $H_2/CO_2 = 4$	277	12	8
MnNiZrRuCe HEMG	flow	0.1 MPa, 330 °C, 22.5 mL min^{-1}, $H_2/CO_2 = 4$	489	450	9
$Ru@Ni_2V_2O_7$	batch	0.1 MPa, 350 °C, $H_2/CO_2 = 4$	115	20	10
Ru/SiO_2	flow	0.1 MPa, 50 mL min^{-1}, $H_2/CO_2 = 6$	55.44	8	11
**Ru/TiO_{2-x}**	**flow**	**0.1 MPa, 30 mL min^{-1}, $H_2/CO_2 = 4$**	**948**	**100**	**This work**

Supplementary References

1. Y. Xie et al., Frustrated lewis pairs boosting low-temperature CO_2 methanation performance over Ni/CeO_2 nanocatalysts. *ACS Catal.* **12**, 10587–10602 (2022).
2. Z. Li et al., Ni-based catalysts derived from layered-double-hydroxide nanosheets for efficient photothermal CO_2 reduction under flow-type system. *Nano Res.* **14**, 4828–4832 (2021).
3. X. Yan et al., Nickel@Siloxene catalytic nanosheets for high-performance CO_2 methanation. *Nat. Commun.* **10**, 2608 (2019).
4. Y. Li et al., Cu-based high-entropy two-dimensional oxide as stable and active photothermal catalyst. *Nat. Commun.* **14**, 3171 (2023).
5. H. Wang et al., Efficient solar-driven CO_2 methanation and hydrogen storage over nickel catalyst derived from metal–organic frameworks with rich oxygen vacancies. *Adv. Mater.* **10**, 2304406 (2023).
6. S. Chen et al., Raising the CO_x methanation activity of a Ru/γ-Al_2O_3 catalyst by activated modification of metal–support interactions. *Angew. Chem. Int. Ed.* **59**, 22763–22770 (2020).
7. J. Zhai et al., Photo-thermal coupling to enhance CO_2 hydrogenation toward CH_4 over Ru/MnO/Mn_3O_4. *Nat. Commun.* **15**, 1109 (2024).
8. J. Ren et al., Targeting activation of CO_2 and H_2 over Ru-loaded ultrathin layered double hydroxides to achieve efficient photothermal CO_2 methanation in flow-type system. *Adv. Energy Mater.* **7**, 1601657 (2017).
9. X. Yu et al., Layered high-entropy metallic glasses for photothermal CO_2 methanation. *Adv. Mater.* **36**, 2312942 (2024).
10. Y. Chen et al., Cooperative catalysis coupling photo-/photothermal effect to drive Sabatier reaction with unprecedented conversion and selectivity. *Joule* **5**, 3235-3251 (2021).
11. Kim C, et al. Energy-efficient CO_2 hydrogenation with fast response using photoexcitation of CO_2 adsorbed on metal catalysts. *Nat. Commun.* **9**, 3027 (2018).
