# Supplementary material for: Origins of the hydrogen spillover effect in d-block metals
Source: Nat Commun. 2026 May 11;17:6317. doi: 10.1038/s41467-026-72608-0 (PMC13376162; doi:10.1038/s41467-026-72608-0)
Supplement: Supplementary file 2 — Transparent Peer Review file [file 41467_2026_72608_MOESM2_ESM.pdf]

nature portfolio

Peer Review File

Origins of the hydrogen spillover effect in d-block metals

Corresponding Author: Professor Zhaosheng Li

This file contains all reviewer reports in order by version, followed by all author rebuttals in order by version.

Version 0:

Reviewer comments:

Reviewer #1

(Remarks to the Author)
The authors calculated descriptors governing the H-spillover on metal/TiO_2 to visualize the hydrogen spillover process, which has been intuitively observed until now. However, the catalysts used and observed phenomena are well known and not novel. The authors just reconstructed and re-described the results of existing studies of hydrogen spillover phenomena and CO_2 hydrogenation using Ru/TiO_{2-x} catalysts from a specific viewpoint, and there is nothing novel about the experimental data in the paper. Although it is interesting as a generalized paper, it is not worthy of acceptance because it lacks novelty in catalytic chemistry. The following are my specific comments

1) Hydrogen is usually dissociated on a catalyst by thermal activation, causing H-spillover. Please state why H_2 activation by photoirradiation was adopted instead of conventional heating. Under light irradiation, H_2 could be activated by carriers generated by photoexcitation of TiO_2, or it could be thermally activated by heat generated by the photothermal effect. In this study, which activation mechanism was H_2 activated? This should be stated based on experimental arguments, for example, by comparing photo-reduction and thermal reduction.

2) I believe the heat generated by photothermal conversion would be the trigger for H_2 activation. If so, to what temperature was the catalyst surface heated? For example, the actual catalyst surface temperature during the PER experiment should be measured by using a radiation thermometer, and the temperature should be stated.

3) If the catalyst surface temperature was increased by light irradiation, the catalyst surface temperature should vary depending on the catalyst (type and amount of supported metal). In that case, hydrogen spillover behavior would depend on the photo-thermal conversion characteristics of each catalyst, therefore, a fair comparison may be impossible.

4) In the SI, the details of the photoexcited reduction (PER) test are not clear. What is the pretreatment temperature? (Removal of physisorbed water and CO_2 must require heating to about 200°C) What are the wavelength and intensity of the irradiated light? This information is necessary for ensuring reproducibility. Also, which detector did you use, TCD or Mass?? Use of Mass (m/z=2) must be required to confirm that all the peaks shown in the PER spectra are H_2.

5) Figure 2c may show the charge bias of each atom in the model displayed in Figure 2b, but the relationship is not clear. The correspondence of each atom should be clearly described.

6) What is the crystal structure of the TiO_2 used, and which TiO_2 crystal face was exposed on the surface of the model used for the DFT calculation? This is because the H-spillover process is highly dependent on the exposed facet of TiO_2.

7) Pd is an effective d-block metal for numerous catalytic reactions. What differences are expected in the case of Pd/TiO_{2-x} compared to Ru/TiO_{2-x}? Some comments and comparisons are needed.

8) In Supplementary Fig. 7, the experimental result and fitting result may be opposite.

9) Regarding CO_2-TPD, I recommend the authors make Kissinger's plots to quantify the CO_2 adsorption energies (the enthalpy for CO_2 desorption), which will differentiate the adsorption strength toward CO_2 over the catalysts.

10) The results shown in Supplementary Fig. 14-17 (catalytic cycles in CO_2 methanation achieved on Ru/TiO_{2-x}) have already been extensively studied in the literature, thereby lacking novelty.

11) The results shown in Supplementary Fig. 18-22 (oxygen vacancy formation evoked by H-spillover on Ru and the concurrent change in absorption spectrum) have already been extensively studied in literature, as well, thereby lacking novelty.

12) The performance of the existing catalysts listed in Supplementary Table 1 is also rather arbitrarily selected; it is well known that the combination of Ru and TiO_2 is highly active in CO_2 methanation, and a comparison with Ni-based catalysts is not fair. Also, conditions such as reaction temperature and flow rate are not listed, therefore, this information does not allow for a fair comparison. At the very least, comparisons with other Ru/TiO_2-based catalysts should be listed, as well as listing reaction conditions.

13) The content is too abstract and generalized, and there is a lack of explanation regarding the scientific meaning of each experimental data and experimental conditions. More detailed explanation of the meaning of each experimental data should be added.

14) The text in the figures is faint, small, and difficult to read. The text in the figures should be corrected.

Reviewer #2

(Remarks to the Author)
It is well established that, despite numerous studies reporting the use of metal-support structures in hydrogenation reactions and hydrogen storage, direct quantification of the hydrogen spillover effect remains largely unexplored, especially thereby impeding the rational design and optimization of catalytic systems in harsh reactions. Li and colleagues developed a well-defined quantitative framework for revealing the interaction between d-block metals and hydrogen molecule, effectively decoupling the activation and spillover regions of the hydrogen spillover effect. A mechanism is proposed with support for the Ru/TiO_{2-x} in light-driven Sabatier reaction, and the authors demonstrate how the use of the quantitative principle allows for a more stable solid catalyst. In general, this is a very timely and original manuscript that enhances researchers' understanding of the dissociation and migration of hydrogen molecules. A comprehensive suite of spectroscopic techniques (PER, XAS, FTIR, etc.) and computational approaches (DFT, AIMD) was employed to rigorously validate their conclusions. These results are impressive and essentially the manuscript reports a major advance. Therefore, I recommend the acceptance of this manuscript after minor revisions according to the comments below.
1. The phrase "reducing the dissociation of the H–H bond and M–H bond" appears in the abstract; however, it is not discussed in the main text. It is recommended that the authors expand on this aspect to enhance clarity and coherence.
2. In the introduction, the transition from the literature review to the dual zones of the hydrogen spillover effect from lines 60–64 on page 3 is abrupt. It is recommended to refine this transition for improved logical flow. Additionally, the presentation of Fig. 1a is not sufficiently concise.
3. Phase and elemental characterizations for samples other than Ru are absent and should be incorporated to ensure a comprehensive analysis.
4. A detailed discussion of the molecular dynamics simulation results is lacking, and the corresponding parameters should be provided for completeness.
5. In the section on the light-driven Sabatier reaction, the discoloration and protection issues of TiO_{2-x} are intriguing. However, the discussion of its protection mechanism in the main text is somewhat brief. It is recommended that the authors elaborate on this aspect to provide a detailed understanding of the mechanism.
6. This manuscript contains several formatting and image-related issues that require clear revision, such as inconsistent ordinate units in Fig. 1b, 2d, and 3d, as well as unclear numerical values in Fig. 2c.

Reviewer #3

(Remarks to the Author)
Hydrogen spillover is a general phenomenon in catalysis that is still poorly understood and requires much deeper comprehension, since it could lead to the development of more efficient hydrogen adsorption materials. By establishing some metrics, the present study has been able to distinguish between the hydrogen activation at the transition metal and its spillover on the TiO_{2-x} with high oxygen vacancies, proposing quantitative and measurable descriptors, whose relevance could be checked in other studies. Publication in Nature Communications is recommended due to the broad impact of the present approach that appears to be applicable to develop advanced hydrogenation catalysts. The authors have, however, to provide a revised version addressing the following points:
• The authors should explain in more how the hydrogen uptake was measured. From the text, line 126 it seems that one part of the measurement is carried out under irradiation and the other under dark. But then, the type of light, when the light is switched off, what happens with spillover if the measurements is made under continuous irradiation and, more important, is the hydrogen activation energy controlling the total amount of spillover hydrogen?
• How do the authors know that in the irradiation period takes place only hydrogen activation without occurring already spillover to the substrate?
• Related with the previous point, I am missing data and discussion of the influence of the temperature, particularly in the range from ambient to 100 oC that is a common temperature range for catalysis of hydrogenation reactions, on the spillover behavior. It is necessary to indicate that the irradiation does not increase the temperature of the material.
• Regarding formula (1) on line 100, it is unclear what is the meaning of the intensity of the photoexcited reduction test. Is this the intensity of the light? In addition, how is the normalization factor "a" calculated? I am missing on this formula some term

corresponding to each transition metal. How can various metals be compared? In my opinion, integration of the area of the peak in the plots of Fig. 1 b between times t0 and t1 will be sufficient. Furthermore, in this plot, rather than intensity I think that the y axis should refer to the percentage of the H2 on the stream that is adsorbed .
• Regarding the activation metrics, as it is now written without appropriate supporting references, it gives the impression that these are original from this study. If it is not, proper credit should be given to the original study in where they were proposed. Then, the novelty of the present study will be much lower.
• The selection of the TiO2 sample used and the reasons behind its chemical modification with NaBH4 to generate oxygen vacancies are unclear.
• The experimental procedure of the photoexcited CO2 desorption tests should be explained in detail. Besides being unclear why light should desorb CO2 from the material in the absence of any reaction, the relationship with hydrogen spillover is unclear.
• Similarly, the way in which the present measurements correlate with the light-driven Sabatier reaction should be introduced better, indicating conditions (batch, light spectrum and intensity, room temperature?), product distribution. Data 13CO2 labeling experiment is necessary.
• The last paragraph before the conclusions section repeats previous comments.

Version 1:

Reviewer comments:

Reviewer #1

(Remarks to the Author)
The manuscript has been adequately revised according to the reviewers' comments. I am satisfied with the revisions provided, so I can now agree to accept them for publication.

Reviewer #2

(Remarks to the Author)
The authors have well addressed all the comments. I thus recommend the publication of this work as is.

Reviewer #3

(Remarks to the Author)
The authors have made some progress in the revision addressing several of my previous points. However, I still think that there are some aspects that would deserve further attention. The authors should consider the following points:

• In their response the authors made clear that light is always on during the duration of the hydrogen uptake by the metal nanoparticles supported on dark TiO2. If this is the case, then, it can be assumed that the two phases, hydrogen activation and hydrogen spill over will take place simultaneously during the whole experiment. Then, how can the authors distinguish between the two phases? Even if hydrogen activation is much faster than the spill over (something to be demonstrated), both hydrogen activation and spill over will occur simultaneously after some time and a spill over phase alone will never take place in their measurements. This point has to be further clarified.
• If I understand properly the response to point 3, the hydrogen uptake experiments are made at temperatures above 450 oC that is much higher than those typically employed in hydrogenation reactions. Therefore, my previous comment 3 is even more important, since the interplay of activation and spill over should certainly depend on the temperature, but this influence is not considered in this study.
• I still think that the author’s response to the previous point 5 to clarify if the proposed spill over metrics is original from this paper or it is based on a prior precedent is not properly addressed. I can see that the experimental setup is new, but I would have liked to see a statement in the text saying that as far as the authors know the proposed metrics in this paper has not been reported earlier.
• When commenting about the selection of Ru nanoparticles on dark TiO2 obtained by NaBH4 treatment at high temperatures I was meaning why not use TiO2 P25 or some other standard material without any modification. TiO2 is widely used as support of metal nanoparticles, but the use of dark TiO2 is considerably less common. I would like to see analogous results using Ru on P25.
• The figure on 13C labelled mass spectrum of CO2 hydrogenation is odd. The reasons why 13CH4 does not undergo fragmentation in the spectrometer showing peaks at m/z 16 and 15 are unclear.
I think that the authors should do still some further amendments before acceptance in Nature Communications

Version 2:

Reviewer comments:

Reviewer #3

(Remarks to the Author)
The authors have addressed most of the previous concerns and I am positive for publication. However, I still do not understand how the two phases, i.e., hydrogen activation and spill over can be differentiated when the two processes are taken places simultaneously. In fact, the authors say in lines 143-145: “As shown in Fig. 1b, a sharp, rapid hydrogen-fragmentation peak (activation zone, t0 to t1) and a broader, slower diffusion peak (spillover zone, t1 to t2).” As if the two processes are occurring consecutively, when it is clearly not the case according to the author’s response. So, I will ask a final effort clarifying better how the authors distinguish the two process and the detailed calculation of all the parameters. I would suggest to do a detailed calculation in the supporting information for one of the catalysts before publication. Apart from this, the manuscript merits publication in Nature Commun.

Version 3:

Reviewer comments:

Reviewer #3

(Remarks to the Author)
I am satisfied with the explanation given and the final changes. Publication is recommended.

Open Access This Peer Review File is licensed under a Creative Commons Attribution 4.0 International License, which permits use, sharing, adaptation, distribution and reproduction in any medium or format, as long as you give appropriate credit to the original author(s) and the source, provide a link to the Creative Commons license, and indicate if changes were made.
In cases where reviewers are anonymous, credit should be given to 'Anonymous Referee' and the source.
The images or other third party material in this Peer Review File are included in the article’s Creative Commons license, unless indicated otherwise in a credit line to the material. If material is not included in the article’s Creative Commons license and your intended use is not permitted by statutory regulation or exceeds the permitted use, you will need to obtain permission directly from the copyright holder.
To view a copy of this license, visit https://creativecommons.org/licenses/by/4.0/

Point-by-Point Reply to the Review Report
(Manuscript ID NCOMMS-25-14125-T)

Manuscript Type: Article

Title: Origins of the hydrogen spillover effect in *d*-block metals

Author(s): Yang Li†, Yuanming Zhang†, Zhaojian Zeng†, Yong Chen, Xiaoming Xu, Zhigang Zou, Zhaosheng Li*

General response: We sincerely thank all reviewers for their valuable and constructive comments, which have helped us significantly improve the quality of our manuscript. The manuscript has been carefully revised to address all the suggestions, as detailed in the point-by-point responses below. (Our response is given in blue, and some key sentences are highlighted; the corrections in the revised manuscript are highlighted).

Reviewer #1:

The authors calculated descriptors governing the H-spillover on metal/TiO_2 to visualize the hydrogen spillover process, which has been intuitively observed until now. However, the catalysts used and observed phenomena are well known and not novel. The authors just reconstructed and re-described the results of existing studies of hydrogen spillover phenomena and CO_2 hydrogenation using Ru/TiO_{2-x} catalysts from a specific viewpoint, and there is nothing novel about the experimental data in the paper. Although it is interesting as a generalized paper, it is not worthy of acceptance because it lacks novelty in catalytic chemistry. The following are my specific comments.

Response: We sincerely thank the reviewer for carefully evaluating our work and for finding our study interesting. We agree that the hydrogen spillover effect on classical metal/TiO_2 systems has been previously reported, primarily in thermal catalytic reactions. Actually, this also includes our group's systematic research (*ACS Sustainable Chem. Eng.* **2022**, 10, 6382–6388), which focused on photothermal catalytic reactions. Due to the active industrial potential of metal/TiO_2, materials and research systems very similar to ours have been repeatedly reported (*Nat Energy* **2024**, 9, 154–162). More importantly, black TiO_{2-x} exhibits strong photothermal conversion capability. In the control experiments, the temperature can be maintained at a consistent level by adjusting the loading of different metals, which facilitates a fair comparison of the hydrogen spillover effects induced by different metals. However, quantitative studies on hydrogen spillover remain limited, and the metal/TiO_{2-x} system serves as a classic model for such investigations.

So, it should be emphasized that the novelty of this work does not stem from merely reconstructing and redefining the Ru/TiO_{2-x} catalyst from a specific perspective. The critical novelty of our study lies in providing a quantitative, descriptor-based framework for understanding and predicting hydrogen spillover at the atomic scale, which has not been achieved in prior studies, metal/TiO_2 serves as a classical research model. And this quantitative technique is an *in-situ* photoexcitation system that was established and further developed by our group (*Natl. Sci. Rev.*, **2025**, 12, nwaf174). Through this approach, we reveal mechanistic insights that are generalizable beyond the specific Ru/TiO_{2-x} system. The descriptors we developed can guide the quantitative design of new catalysts for catalytic systems (Such as, Ni-based alloys, Ru-based alloys) and other hydrogen-involving reactions without relying on trial and error, making our contribution both novel and broadly impactful. Meanwhile, molecular dynamics simulations of hydrogen spillover, combined with density functional theory calculations, revealed that the nonlinear spillover capacity and rate of *d*-block metals arise from the cooperative overlap of their unoccupied *d*–*s* orbitals and the interaction between the σ and σ* orbitals of hydrogen atoms, forming a weak antibonding state that reduces the dissociation of both H-H and M-H bonds.

In summary, the key innovations of our study are: (i) proposing quantitative descriptors of the hydrogen spillover effect to guide the design of hydrogenation and hydrogen storage materials; (ii) developing an *in situ* photoexcitation reduction method to quantitatively distinguish the activation and spillover zones; (iii) demonstrating

through molecular dynamics and first-principles calculations that orbital coupling enhances the hydrogen spillover effect of *d*-block metals. Building on this understanding, our Ru cluster modified TiO_{2-x} catalyst exhibited both excellent durability and high activity, addressing the limitations reported in previous studies. We believe that demonstrating this previously unexplored quantitative technique and mechanism meets the novelty and significance expected for *Nature Communications* and will attract broad interest. We have addressed all of the reviewers' comments, and below is our detailed point-by-point response.

1) Hydrogen is usually dissociated on a catalyst by thermal activation, causing H-spillover. Please state why H_2 activation by photoirradiation was adopted instead of conventional heating. Under light irradiation, H_2 could be activated by carriers generated by photoexcitation of TiO_2, or it could be thermally activated by heat generated by the photothermal effect. In this study, which activation mechanism was H_2 activated? This should be stated based on experimental arguments, for example, by comparing photo-reduction and thermal reduction.

Response: We are grateful to the reviewer for the insightful questions and constructive comments, which inspired us to conduct extensive additional information and analyses to further strengthen our conclusions.

First, our research focuses on photothermal synergistic catalytic reactions, which have attracted increasing attention in recent years due to their efficient utilization of solar energy, including our group (*Joule* **2021**, 5, 3235–3251, Highly Cited Papers; *Joule* **2024**, 8, 3126–3141; *ACS Catal.* **2025**, 15, 10470–10479; *Adv. Funct. Mater.* 2025, 2500357) and other groups (*Nat. Wat.* **2023**, 1, 952–960; *Nat. Commun.* **2024**, 15, 5639). In such reactions, the hydrogen spillover process plays a critical role; however, studies on hydrogen spillover under light irradiation remain lacking. Therefore, in this work, we primarily employ photoexcitation to activate H_2.

Second, regarding the hydrogen activation mechanism in photothermal synergistic catalysis, we intend to conduct a detailed discussion of the photothermal process before proceeding to a comprehensive summary. Ground in the fundamental principles of catalysis, photothermal catalysis can be broadly categorized into three primary reaction types: photothermal heating catalysis, thermally assisted photocatalysis and photothermal synergistic catalysis (*Nat. Commun.* **2019**, 10, 2359; *Energy Environ. Sci.* **2019**, 12, 1122–1142; *Angew. Chem. Int. Ed.* **2023**, 62, e202302253; *Nat. Commun.* **2023**, 14, 3171; *Nat. Commun.* **2024**, 15, 7974). Next, we discuss the mechanistic differences among these catalytic approaches.

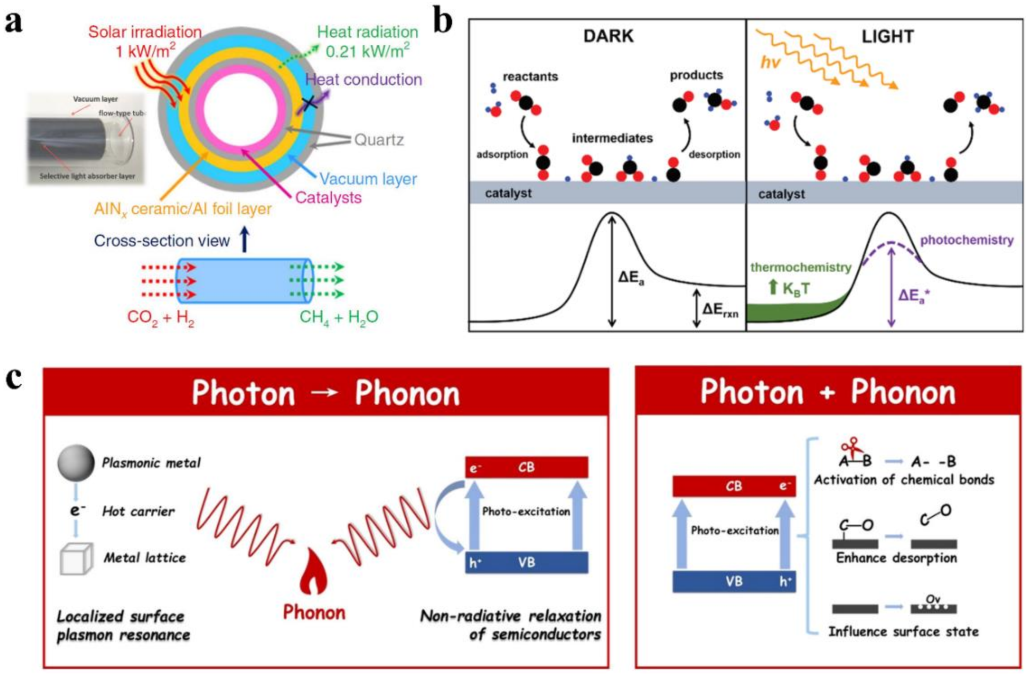

Fig. R1 | Three categories of photothermal catalysis. a, Photothermal heating catalysis (*Nat. Commun.* **2019**, 10, 2359). **b**, Thermally assisted photocatalysis (*Energy Environ. Sci.* **2019**, 12, 1122–1142). **c**, Photothermal synergistic catalysis (*Nat. Commun.* **2024**, 15, 7974).

Photothermal heating catalysis functions through a mechanism analogous to thermal catalysis, leveraging the robust light absorption capabilities of specialized reactors or catalysts to efficiently convert light energy into thermal energy. This process facilitates the progression of reactions at elevated temperatures, thereby enhancing the catalytic performance. As depicted in **Fig. R1a**, researchers have developed an innovative reactor design. The reactor's outermost layer, composed of quartz, permits the transmission of light, while an internal vacuum layer is integrated to substantially minimize heat loss via conduction. Positioned between the vacuum layer and the inner quartz wall is a light-absorbing material that effectively harnesses sunlight, converting it into thermal energy and thereby increasing the reactor's temperature. The catalysts are strategically placed within a quartz tube, enabling the subsequent reactions to proceed efficiently.

In thermally assisted photocatalysis, as illustrated in **Fig. R1b**, both photochemical and thermochemical processes synergistically contribute to the overall reaction rate. The photochemical component of photothermal catalysis enhances the reaction kinetics by reducing the activation energy barrier of one or more critical reaction steps, thereby accelerating the process. Physically, the reduction in the activation barrier stems from charge transfer to the catalyst surface, which enhances the acidity or basicity of the active sites, or from energy transfer to a vibrational mode of the transition state, thereby influencing bond elongation and facilitating reaction progression.

Synergistic photothermal catalysis utilizes a supported catalytic system, typically comprising a stable metal oxide as the support with metal dispersed on its surface. As depicted in **Fig. R1c**, this mode differs fundamentally from the previous two modes by leveraging cooperative interactions among photons, phonons, and electron–hole pairs,

thereby enabling a highly integrated catalytic mechanism (*Chem. Rev.* **2011**, 111, 3858–3887; *Nat. Nano.* **2015**, 10, 25–34; *Nat. Catal.* **2018**, 1, 656–665; *Chem. Soc. Rev.* **2021**, 50, 2173–2210; *Nat. Commun.* **2024**, 15, 7974). Light initially induces distinct reaction pathways depending on the electronic structure of the material. Under solar radiation, the metal oxide catalyst substrate absorbs light of specific wavelengths, exciting electrons to transition from the valence band to the conduction band, leaving behind holes and forming electron–hole pairs. These electrons and holes rapidly thermalize, reaching the bottom of the conduction band and the top of the valence band, respectively. They may subsequently either migrate away from their generation sites through charge separation or recombine rapidly via radiative or non-radiative processes. In addition, metal supported on oxide substrates can efficiently respond to incident light. Part of the absorbed energy is converted into hot electrons (high-energy, non-equilibrium state electrons) through non-radiative relaxation processes. These hot electrons can accelerate the activation of reactants. The relaxation of these hot electrons transfers heat through electron–phonon coupling, leading to a substantial local temperature increase, which creates favourable conditions for chemical reactions.

Next, we discuss how hot electrons influence subsequent catalytic chemical processes. As shown in **Fig. R2**, in the initial stages, a significant population of hot electrons are generated at the metal surface. If the activation of reactant molecules occurs at the metal surface (for example, in hydrogenation reactions, such as the activation of H_2), the hot electrons can be directly injected into the reactant molecule surface. This injection affects the bond length and bond angles of the molecule, thereby facilitating its activation. Second, if the activation of the reactant molecule takes place on the surface of the support, hot electrons can transfer their energy through relaxation, thereby accelerating the activation process. This heat transfer involves the movement of hot carriers, and in such catalytic systems, thermal conduction remains the most crucial mode of heat transfer within the catalytic material.

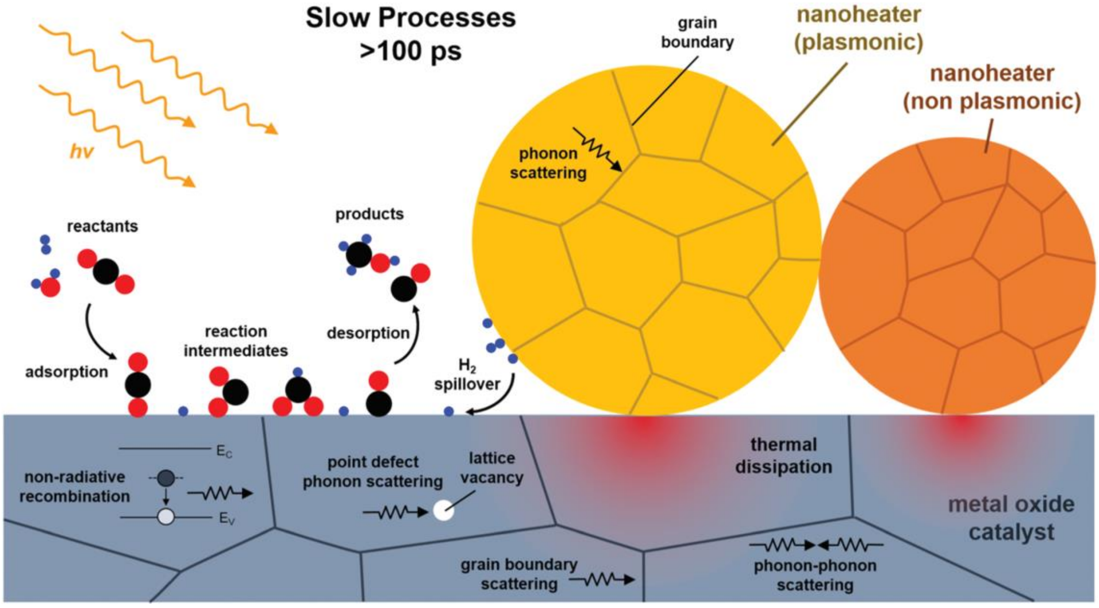

Fig. R2 | Slow photothermal processes occurring in a metal/metal-oxide heterostructured catalyst under solar irradiation. The black, red, and blue circles denote carbon, oxygen, and hydrogen, respectively (*Energy Environ. Sci.*, **2019**,12, 1122-1142).

For nanometals, electrons serve as the primary carriers of thermal energy, with phonon thermal conductivity playing a secondary role. The thermal resistance of metals is governed mainly by electron–phonon scattering and geometric scattering. In contrast, metal oxide semiconductors rely predominantly on phonons for heat conduction, with factors such as grain boundary scattering, defect scattering, and phonon–phonon interactions significantly impacting their thermal conductivity. Compared with metal, most supports have relatively low thermal conductivity. As a result, the energy released during hot electron relaxation is primarily concentrated at the adjacent interface between the metal and the support, causing a substantial increase in temperature at this interface. This localized heating greatly enhances the activation of reactant molecules, thereby facilitating the catalytic process.

Photothermal synergistic catalysis ultimately requires efficient thermal conductivity between the metal and the support, while the catalyst itself should possess low thermal conductivity to concentrate the thermal energy at the reaction sites. The effects of light absorption and thermal confinement lead to an increase in the local temperature within nanoscale structures, and this effect becomes more pronounced as the catalyst size decreases. Therefore, a critical technical challenge is to understand and control the thermal resistance between grains and across particle interfaces, as this resistance ultimately governs the thermal transport properties of the catalytic system and the temperature at the active sites. Leveraging the high efficiency of photothermal synergistic catalysis, researchers have made remarkable progress in applying it to areas such as CO_2 reduction, dry reforming of methane and ammonia synthesis (*Nat. Commun.* **2019**, 10, 2608; *Nano Res.* **2021**, 14, 4828–4832; *ACS Catal.* **2022**, 12, 10587–10602; *Nat. Commun.* **2023,** 14, 3171; *Adv. Mater.* **2023**, 10, 2304406).

Recent reviews have affirmed the photothermal synergistic mechanism and highlighted the importance of our group's contributions in developing this photothermal synergistic catalysis system (*Energy Environ. Sci*., **2024**, 17, 4461; *Adv. Mater.* **2025**, 37, 2312093). We already have a solid understanding of the system, and our research is not aimed at repeating previous studies but at further advancing its mechanistic understanding. Therefore, we believe that in photothermal synergistic catalysis, hot electrons generated by metal can contribute to the efficient activation of H_2. These hot electrons directly participate in electronic interactions with molecular orbitals, weakening the H−H bond and thereby enabling highly efficient hydrogen activation. The migration of photogenerated electrons from TiO_2 to metal promotes the CO_2 hydrogenation reaction at the metal/TiO_{2-x} catalyst interface.

Furthermore, durability tests of the two catalysts clearly show that TiO_2 without metal loading has weak activation of photoexcited electrons, producing almost no product during long-term CO_2 hydrogenation (**Fig. R3**, this figure corresponds to Supplementary Fig. 16). TiO_{2-x} initially exhibits moderate activity (still two orders of magnitude lower than the metal/TiO_{2-x}) but gradually deactivates due to lattice reconstruction, indicating insufficient replenishment of highly reduced hydrogen species (**Fig. R4**, this figure corresponds to Supplementary Fig. 17). This is also consistent with the observation that TiO_{2-x} did not exhibit a clearly defined activation and spillover zone in the photoexcited reduction test (**Fig. R5**, this figure corresponds

to Fig. 1f). Taken together, these results suggest that hydrogen activation in this system primarily occurs via the hot electron mechanism.

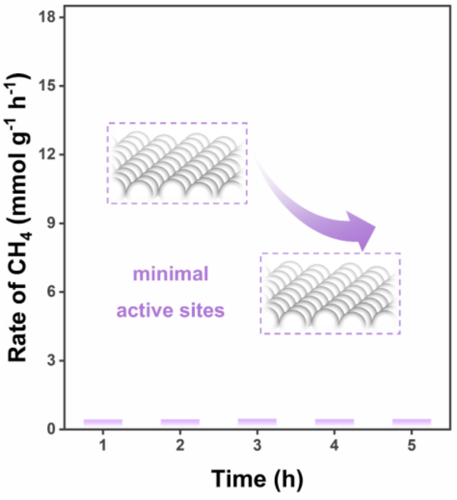

Fig. R3 | Light-driven CO_2 reduction test for the TiO_2 sample.

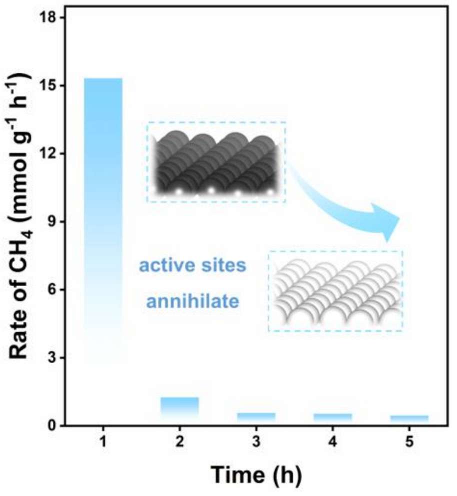

Fig. R4 | Light-driven CO_2 reduction test for the TiO_{2-x} sample.

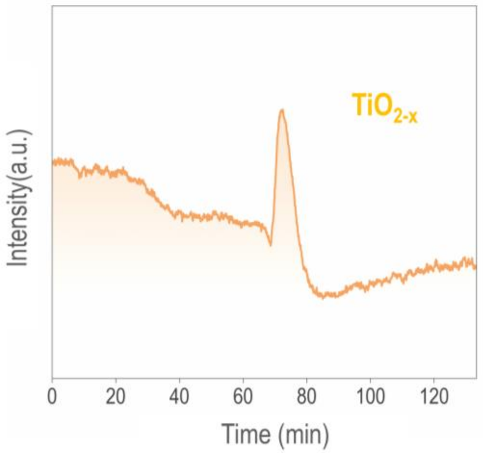

Fig. R5 | Photoexcited reduction test for the TiO_{2-x} sample.

2) I believe the heat generated by photothermal conversion would be the trigger for H_2 activation. If so, to what temperature was the catalyst surface heated? For example, the actual catalyst surface temperature during the PER experiment should be measured by using a radiation thermometer, and the temperature should be stated.

Response: We appreciate the reviewer's comment. As shown in **Fig. R6a** (this figure corresponds to Supplementary Fig. 2a), in our PER experiments using a 300 W Xenon lamp (CEL-PF300-T8, CEAuLight Co., Ltd., China) with a full spectrum irradiation intensity of approximately 1 W cm^{-2}, the solid catalyst was confined within a thin quartz tube, and the metal loading was controlled below 1%. Owing to the excellent photothermal conversion capability of the black TiO_{2-x} support, a significant surface temperature rise was observed under illumination. Although a small amount of metal loading can further influence the localized photothermal effect, the overall surface temperatures of different metal/TiO_{2-x} systems remained in a narrow range of 466–468 °C (**Fig. R6b and Fig. R7,** this figure corresponds to Supplementary Fig. 2b and Supplementary Fig. 3). This relatively small temperature variation is insufficient to account for the observed hydrogen activation, underscoring that the hot electron mechanism originated from different metals, rather than simple thermal heating, plays the dominant role in this system.

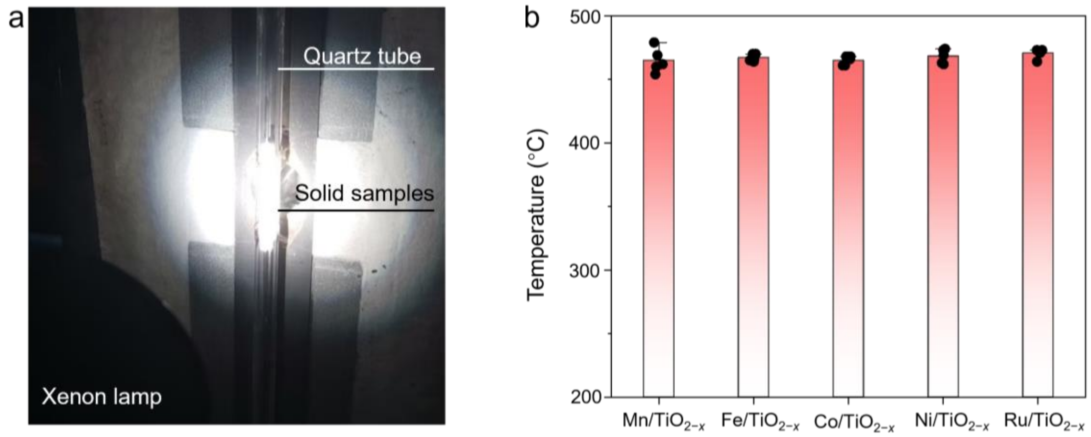

Fig. R6 | Real-time temperature monitoring for photoexcited reduction (PER) testing. a, Optical image of the local structure in the PER test. **b**, Average temperature distribution from an infrared (IR) camera under 300 W Xe lamp irradiation in the PER test. The IR images of the sample surface in the reactor were captured by an IR camera (FLUKE, TiX580) with the corresponding emissivity and were analysed by software (SmartView).

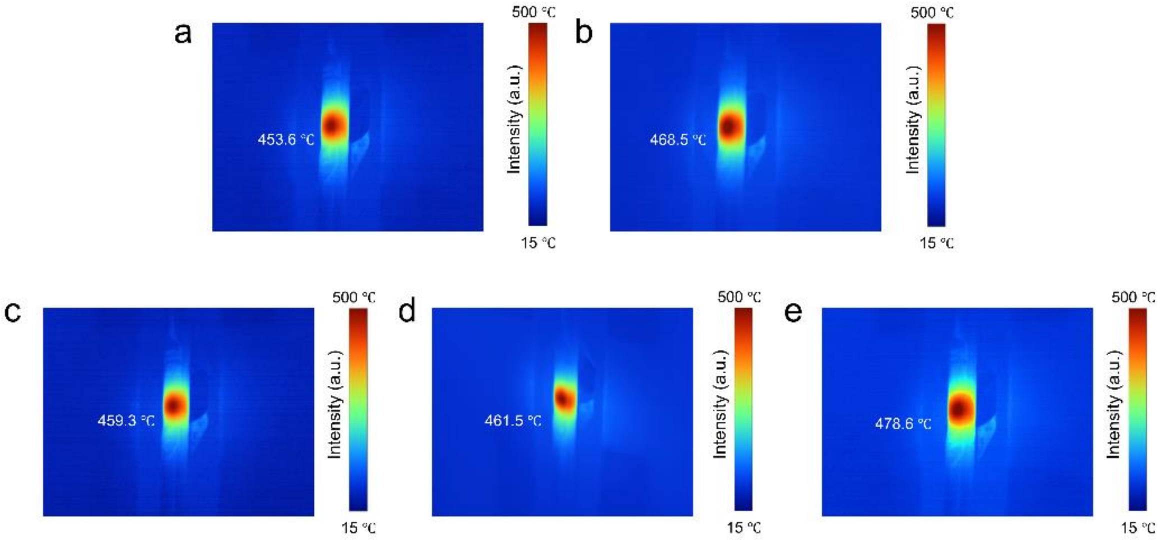

Fig. R7 | IR images of the solid catalyst recorded after the same-duration 300 W Xe-lamp irradiation. **a**, Mn/TiO_{2-x}; **b**, Fe/TiO_{2-x}; **c**, Co/TiO_{2-x}; **d**, Ni/TiO_{2-x}; **e**, Ru/TiO_{2-x}.

3) If the catalyst surface temperature was increased by light irradiation, the catalyst surface temperature should vary depending on the catalyst (type and amount of supported metal). In that case, hydrogen spillover behavior would depend on the photo-thermal conversion characteristics of each catalyst, therefore, a fair comparison may be impossible.

Response: We thank the reviewer for this insightful comment. Indeed, the incorporation of different types and amounts of metal can influence local photothermal conversion, leading to some variation in surface temperature. To minimize this effect, we monitored the catalyst temperature in real time and conducted temperature-matched control experiments, and the metal loading was controlled to remain below 1%. As shown in **Fig. R6** and **R7**, the overall surface temperature variation under our experimental conditions was relatively small (approximately 450–480 °C). Importantly, even under identical thermal conditions, significant differences in hydrogen spillover were still observed. This indicates that the dominant factor is the hot electrons generated at the metal–semiconductor interface, which directly weaken the H–H bond and facilitate the spillover process. Thus, while minor thermal effects are present, the primary contribution arises from hot electron dynamics, making the comparative analysis between different catalysts both valid and meaningful.

4) In the SI, the details of the photoexcited reduction (PER) test are not clear. What is the pretreatment temperature? (Removal of physisorbed water and CO_2 must require heating to about 200 °C) What are the wavelength and intensity of the irradiated light? This information is necessary for ensuring reproducibility. Also, which detector did you use, TCD or Mass? Use of Mass (m/z=2) must be required to confirm that all the peaks shown in the PER spectra are H_2.

Response: We appreciate the reviewer's comment. Prior to formal testing, the catalyst was pretreated at 200 °C in an Ar atmosphere for 30 min to ensure complete removal of CO_2 and H_2O. The experiments employed a 300 W Xenon lamp (CEL-PF300-T8,

CEAuLight Co., Ltd., China) as the light source, with a full spectrum irradiation intensity of approximately 1 W cm^{-2}. A TCD detector was used for quantitative detection and analysis. In the PER test, a high-purity H_2/Ar mixed gas was introduced, the observed peak changes corresponded exclusively to variations in H_2, and no peaks were detected under a pure Ar atmosphere.

Actions: This information has been added to the revised Supplementary Information.

"Photoexcited reduction (PER) test

In order to study the hydrogen spillover process under *in situ* photoexcitation, we designed a photoexcited analyser. Prior to formal testing, the catalyst was pretreated at 200 °C in an Ar atmosphere for 30 min to ensure complete removal of CO_2 and H_2O. Then, H_2/Ar was used to blow over the surface of the sample to be tested at a certain flow rate for one hour. After that, the experiments employed a 300 W Xenon lamp (CEL-PF300-T8, CEAuLight Co., Ltd., China) as the light source, with an irradiation intensity of approximately 1 W cm^{-2}. A TCD detector was used for quantitative detection and analysis. In the PER test, a high-purity H_2/Ar mixed gas was introduced, the observed peak changes corresponded exclusively to variations in H_2, and no peaks were detected under a pure Ar atmosphere."

5) Figure 2c may show the charge bias of each atom in the model displayed in Figure 2b, but the relationship is not clear. The correspondence of each atom should be clearly described.

Response: Thank the reviewer very much. As shown in **Fig. R8c** (this figure corresponds to revised Fig. 2c), it primarily illustrates the charge transfer among H, Ru, and TiO_{2-x} during the hydrogen spillover process when in contact with hydrogen atoms. H_1 and H_2 denote the hydrogen atoms in a hydrogen molecule, while Ru_1 and Ru_2 are simply labels within the Ru cluster without specific physical significance. The migration of hydrogen from the support into the interior is energetically unfavourable (**Fig. R8b**, this figure corresponds to revised Fig. 2b), indicating that the hydrogen spillover primarily occurs on the interface. Combined with density functional theory (DFT) calculations, we analysed the energy and charge transfer processes: during H_2 activation on the Ru surface, charge transfers from Ru to hydrogen. One hydrogen atom (H_2) remains on the Ru cluster, while the other (H_1) spills over to the support, transferring charge to the support surface. This behavior is consistent with the activation and spillover processes observed in our PER experiments.

Actions: We have added detailed description in Figure 2b and c in the revised main text.

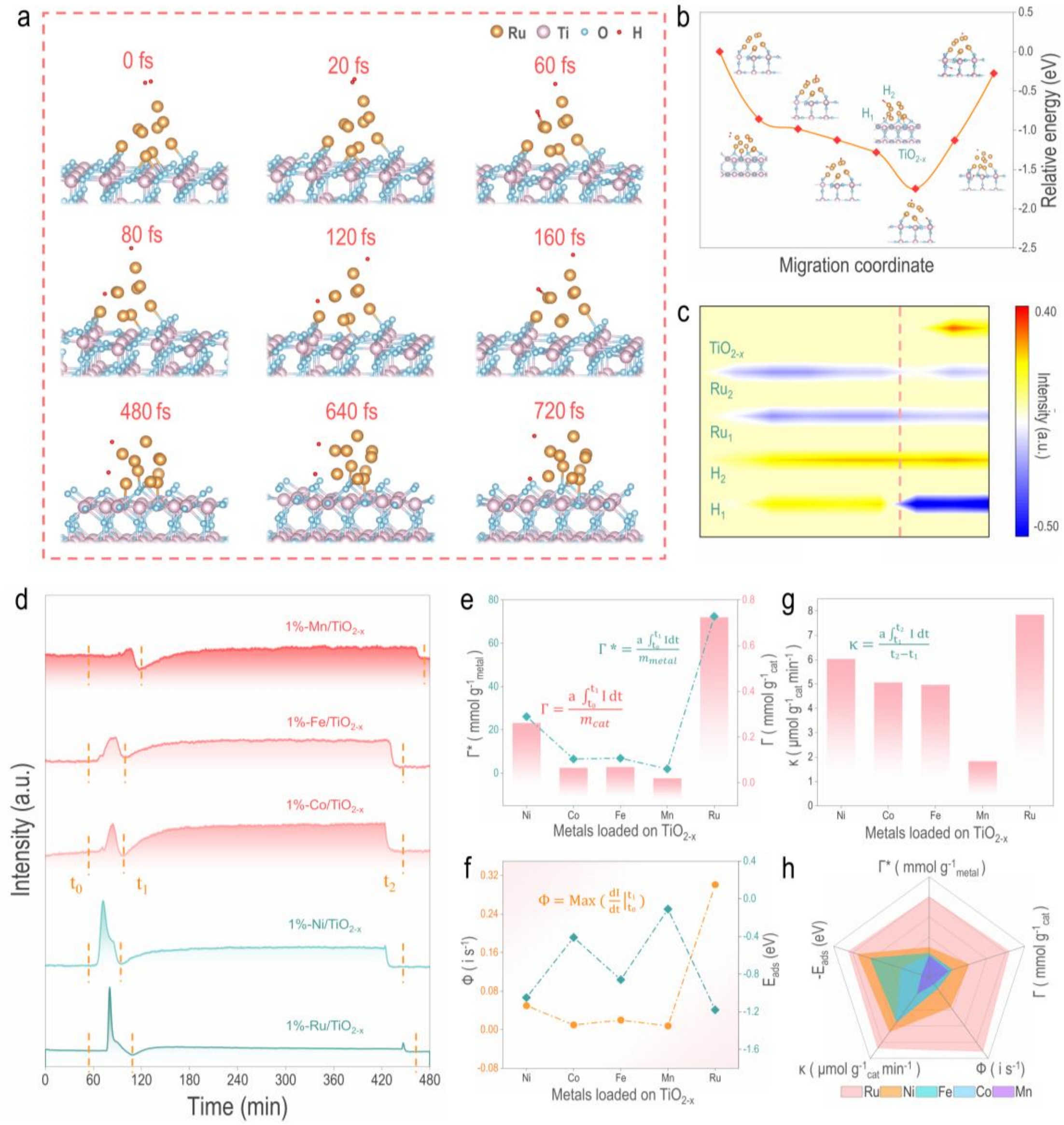

Fig. R8 | Simulation-assisted hydrogen spillover visualization and universal analysis of *d*-block metals. a, Molecular dynamics simulation visualizing the process of hydrogen spillover. **b**, Change in the Gibbs free energy of the hydrogen spillover process on the Ru/TiO_{2-x} sample. **c**, Bard charge transfer during the hydrogen spillover process on the Ru/TiO_{2-x} sample. H_1 and H_2 denote the hydrogen atoms in a hydrogen molecule, while Ru_1 and Ru_2 are simply labels within the Ru cluster without specific physical significance. **d**, Photoexcited reduction test for the Mn/TiO_{2-x}, Fe/TiO_{2-x}, Co/TiO_{2-x}, Ni/TiO_{2-x} and Ru/TiO_{2-x} samples. **e**, Statistics of Γ and Γ^* for all the samples. **f**, Statistics of Φ and hydrogen dissociation energy for all the samples. E_{ads} is the hydrogen adsorption energy calculated by DFT. **g**, Statistics of κ for all the samples. **h**, Comparison of descriptors (Γ, Γ^*, Φ, κ and E_{ads}) for all the samples.

6) What is the crystal structure of the TiO_2 used, and which TiO_2 crystal face was exposed on the surface of the model used for the DFT calculation? This is because the H-spillover process is highly dependent on the exposed facet of TiO_2.

Response: We sincerely thank the reviewer's question. To minimize potential errors introduced during sample preparation, commercial P25 was employed as the catalyst

support in this study. As shown in **Fig. R9** (this figure corresponds to Supplementary Fig. 5), the crystal structure remained unaltered after metal deposition. Based on the XRD results, where the (101) peak is the most intense, the TiO_2 (101) surface was used as the model in our DFT calculations, representing the predominant exposed facet.

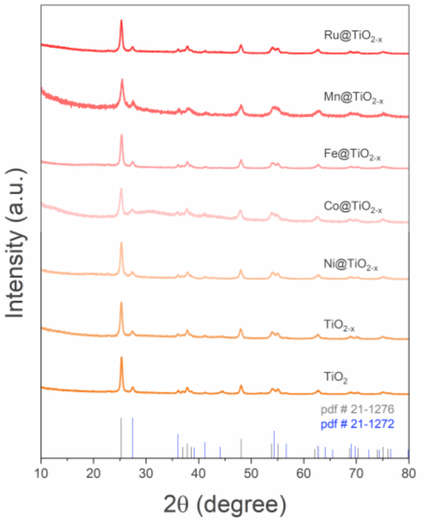

Fig. R9 | XRD patterns of the TiO_2, TiO_{2-x}, Ru/TiO_{2-x}, Mn/TiO_{2-x}, Fe/TiO_{2-x}, Co/TiO_{2-x} and Ni/TiO_{2-x} samples (pdf # 21–1276 represent rutile TiO_2, pdf # 21–1272 represent anatase TiO_2).

7) Pd is an effective *d*-block metal for numerous catalytic reactions. What differences are expected in the case of Pd/TiO_{2-x} compared to Ru/TiO_{2-x}? Some comments and comparisons are needed.

Response: We greatly appreciate the reviewer's valuable suggestion. We have supplemented the study with Pd-loaded catalysts. As shown in **Fig. R10** (this figure corresponds to Supplementary Fig. 4), Pd metal are uniformly dispersed on the TiO_{2-x} support. During the PER test, the processes of hydrogen activation and spillover for Pd metal can be clearly observed. Quantitative analysis reveals that all performance metrics for Pd are lower than those for Ru, indicating that Pd exhibits a weaker capability for hydrogen activation. As shown in **Fig. R8g** (this figure corresponds to Fig. 2g)**,** we also observed that the κ value gradually increases with the metal atomic number. This trend arises because the degree of *d*–*s* hybridization progressively increases from Mn ($3d^5 4s^2$) to Ru ($4d^7 5s^1$) among the *d*-block metals, whereas for Pd the *d*–*s* hybridization decreases owing to its full-shell $4d^{10}5s^0$ configuration and the deep energetic stabilization of the 4*d* band.

Actions: We have added the above discussion to the revised main text and Supplementary Information.

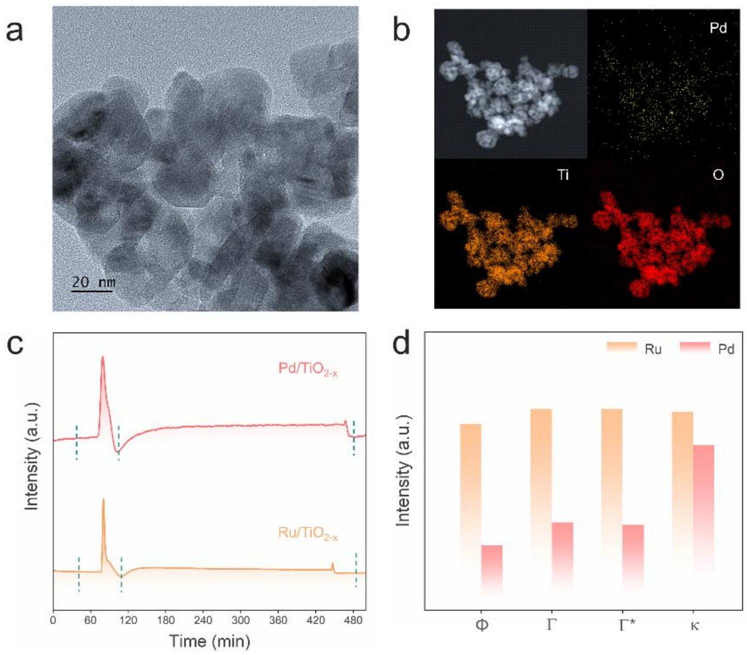

Fig. R10 | Structural characterization and photoexcited reduction test evaluation of Pd/TiO_{2-x}. **a**, **b**, Transmission electron microscopy (TEM) and Energy dispersive X-ray (EDX) mapping images of of Pd/TiO_{2-x}. **c**, Photoexcited reduction test for Pd/TiO_{2-x}. **d**, Relative numerical evaluation of descriptors (Γ, Γ*, Φ, and κ) for Pd/TiO_{2-x} and Ru/TiO_{2-x}.

8) In Supplementary Fig. 7, the experimental result and fitting result may be opposite.

Response: We thank the reviewer for pointing out this issue. We apologize for the mislabelling, as shown in **Fig. R11** (this figure corresponds to the revised Supplementary Fig. 11, which was originally Supplementary Fig. 7), which has now been corrected. In addition, we have carefully reviewed the entire manuscript to prevent similar errors.

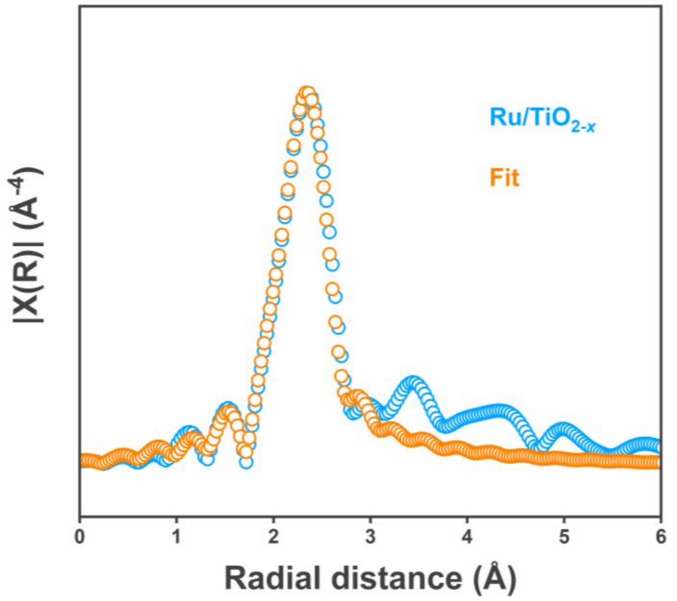

Fig. R11 | EXAFS R-space curves of Ru/TiO_{2-x}.

9) Regarding CO_2-TPD, I recommend the authors make Kissinger's plots to quantify the CO_2 adsorption energies (the enthalpy for CO_2 desorption), which will differentiate the adsorption strength toward CO_2 over the catalysts.

Response: We sincerely thank the reviewer for this valuable suggestion. We agree that Kissinger analysis of CO_2-TPD data can provide quantitative insight into CO_2 adsorption energies. As shown in **Fig. R12** (this figure corresponds to Supplementary Fig. 14), the apparent activation energy (E_a) for CO_2 desorption was estimated using Kissinger's method. According to the Kissinger equation, $\ln(\beta/T_p^2) = (-E_a/R) * (1/T_p) + \ln(AR/E_a)$, where β represents the heating rate, T_p represents the peak temperature, E_a represents the apparent activation energy, R represents the gas constant (8.314 J $mol^{-1} \cdot K^{-1}$), and A represents the pre-exponential factor. By performing CO_2-TPD experiments at different heating rates and plotting $\ln(\beta/T_p^2)$ versus $1/T_p$, a linear fit was obtained. The slope of the line ($-E_a/R$) was used to calculate the apparent activation energy. The CO_2 desorption peak shifts significantly toward higher temperatures with increasing heating rates. On the basis of Kissinger plots, the CO_2 adsorption energy can be calculated as 3.123 kJ/mol.

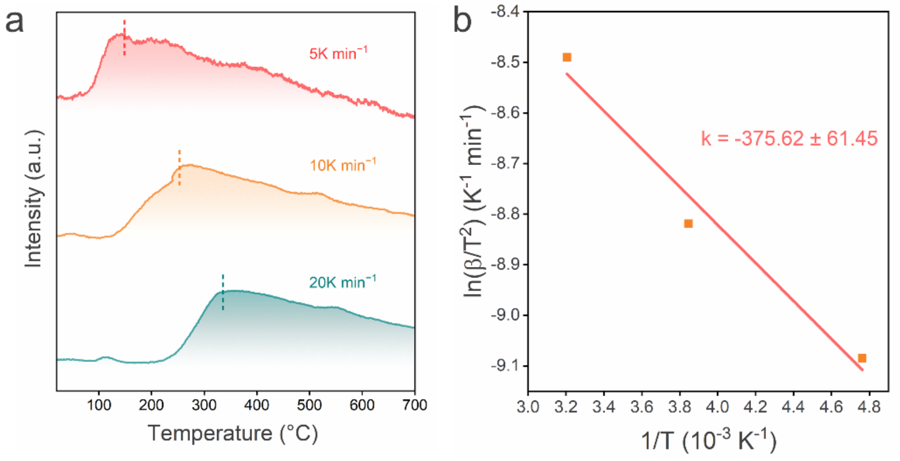

Fig. R12 | a, CO_2-TPD profiles for Ru/TiO_{2-x} at different heating rates. **b,** Calculation of the CO_2 desorption energy on the basis of Kissinger plots.

10) The results shown in Supplementary Fig. 14-17 (catalytic cycles in CO_2 methanation achieved on Ru/TiO_{2-x}) have already been extensively studied in the literature, thereby lacking novelty.

Response: We thank the reviewer for their valuable comments. While we acknowledge that CO_2 methanation is a widely studied system, the core innovation of this work lies not in re-examining the catalytic system, but in employing a photoexcitation reduction method to reveal, for the first time, the hydrogen spillover effect under light irradiation and to propose a set of descriptors for its quantitative analysis. By comparing various quantitative indicators, we demonstrate the high efficiency of Ru in promoting the hydrogen spillover process under the flow system, which may also inspire new catalyst designs. The catalytic cycle summarized in the original Supplementary Figs. 14–17 (these figures correspond to revised Supplementary Fig. 16–18 and Fig. 4e of the main text) primarily illustrates, from the perspective of the catalytic reaction, the promoting

effect of Ru on hydrogen activation, thereby validating the applicability of the proposed quantitative descriptors.

11) The results shown in Supplementary Fig. 18-22 (oxygen vacancy formation evoked by H-spillover on Ru and the concurrent change in absorption spectrum) have already been extensively studied in literature, as well, thereby lacking novelty.

Response: We thank the reviewer for this valuable comment. Although it is true that oxygen vacancy formation induced by hydrogen spillover and the associated absorption changes have been reported in the literature, those studies were primarily conducted under thermal or non-photothermal conditions, rather than in a photothermal synergistic catalytic system as in our work. This part has the following features.

Photoexcitation conditions: All observations were made under light irradiation, allowing us to directly demonstrate hydrogen spillover under photoexcited conditions, which has not been explored previously.

Quantitative descriptors: Supplementary Figs. 18–22 (these figures correspond to revised Supplementary Fig. 20–24) are used to illustrate and validate the proposed set of quantitative descriptors for hydrogen spillover, providing a systematic framework that goes beyond qualitative descriptions in prior studies.

Mechanistic understanding: These results serve to highlight the interplay between Ru, H-spillover, and oxygen vacancy formation under photoexcitation, supporting the mechanistic interpretation of our catalytic system rather than being presented as the primary novel finding.

Taken together, although aspects of hydrogen spillover and oxygen vacancies have been reported, the combination of photoexcitation, quantitative analysis, and mechanistic insight in our study represents a clear advancement over previous work.

12) The performance of the existing catalysts listed in Supplementary Table 1 is also rather arbitrarily selected; it is well known that the combination of Ru and TiO_2 is highly active in CO_2 methanation, and a comparison with Ni-based catalysts is not fair. Also, conditions such as reaction temperature and flow rate are not listed, therefore, this information does not allow for a fair comparison. At the very least, comparisons with other Ru/TiO_2-based catalysts should be listed, as well as listing reaction conditions.

Response: Thank the reviewer's question. This study focuses on the quantitative decoupling of the activation and spillover zones of the hydrogen spillover process under light-driven conditions. The established thermodynamic–kinetic descriptors, combined with theoretical calculations, are applicable across a range of *d*-block metals and reveal that the hydrogen spillover effect originates from the intrinsic properties of metal *d*–*s* orbital hybridization. Partially vacant *d*–*s* orbitals can cooperatively overlap with the σ and σ* orbitals of adsorbed hydrogen, forming weak antibonding states that partially weaken H–H and M–H bonds. This orbital-level interaction lowers the energy barrier for hydrogen dissociation and migration, providing a mechanistic explanation for the enhanced hydrogen spillover behaviour of *d*-block metals. These findings

quantitatively explain the superiority of Ru and offer a framework for developing new catalysts, reducing reliance on trial-and-error approaches.

Based on these insights, we optimized a highly efficient catalyst with a Ru loading less than 1%. Following the reviewer's suggestion, we have supplemented the detailed experimental conditions for each catalyst. The activities of representative Ni- and Ru-based catalysts are reported in **Table R1** (corresponding to Supplementary Table 1). Compared with these catalysts, our Ru/TiO_2 exhibits excellent activity and stability in the photothermal flow catalytic system, consistent with and further supported by the hydrogen spillover effect discussed in this study.

Table R1 | Comparison with representative catalysts for light-driven and external heat-driven Sabatier reactions. The blue background represents hybrid catalysts containing Ni or other metals, while the pink background represents hybrid catalysts containing Ru.

Catalyst	Reactor	Operation Parameters	CH_4 yield (mmol g_{cat}^{-1} h^{-1})	Stability (h)	References
Ni/CeO_2	flow	0.1 MPa, 225 °C (external), 50 mL min^{-1}, H_2/CO_2 = 4	147.1	150	1
Ni/Al_2O_3	flow	0.1 MPa, 290 °C,10 mL min^{-1}, $H_2/CO_2/Ar$ = 60/15/25	279	100	2
NiO@SiXNS	flow	0.1 MPa, 300 °C, 5 mL min^{-1}, H_2/CO_2 = 4	100	12	3
$Cu_2Zn_1Al_{0.5}Ce_5Zr_{0.5}O_x$	flow	0.1 MPa, 500 °C, 4000 mL min^{-1}, H_2/CO_2 = 1	417.2	10	4
Ni/ZrO_2	flow	0.1 MPa, 225 °C, 50 mL min^{-1}, H_2/CO_2 = 4	583.3	10	5
$Ru/\gamma\text{-}Al_2O_3$	flow	0.1 MPa, 350 °C (external), 41.6 mL min^{-1}, 15.5% CO_2, 80.9% H_2	137.88	15	6
$Ru/MnO/Mn_3O_4$	batch	180 mL, 1MPa, H_2/CO_2 = 4	166.7	5	7
Ru@FL-LDH	flow	0.1 MPa, 350 °C, 125.0 mL min^{-1}, H_2/CO_2 = 4	277	12	8
MnNiZrRuCe HEMG	flow	0.1 MPa, 330 °C, 22.5 mL min^{-1}, H_2/CO_2 = 4	489	450	9
$Ru@Ni_2V_2O_7$	batch	0.1 MPa, 350 °C, H_2/CO_2 = 4	115	20	10 (Our group)
Ru/SiO_2	flow	0.1 MPa, 50 mL min^{-1}, H_2/CO_2 = 6	55.44	8	11
**Ru/TiO_{2-x}**	**flow**	**0.1 MPa, 30 mL min^{-1}, H_2/CO_2 = 4**	**948**	**100**	**This work**

References

[1] Y. Xie et al., Frustrated lewis pairs boosting low-temperature CO_2 methanation performance over Ni/CeO_2 nanocatalysts. *ACS Catal.* **12**, 10587–10602 (2022).

[2] Z. Li et al., Ni-based catalysts derived from layered-double-hydroxide nanosheets for efficient photothermal CO_2 reduction under flow-type system. *Nano Res.* **14**, 4828–4832 (2021).

[3] X. Yan et al., Nickel@Siloxene catalytic nanosheets for high-performance CO_2 methanation.

Nat. Commun. **10**, 2608 (2019).
[4] Y. Li et al., Cu-based high-entropy two-dimensional oxide as stable and active photothermal catalyst. *Nat. Commun.* **14**, 3171 (2023).
[5] H. Wang et al., Efficient solar-driven CO_2 methanation and hydrogen storage over nickel catalyst derived from metal–organic frameworks with rich oxygen vacancies. *Adv. Mater.* **10**, 2304406 (2023).
[6] S. Chen et al., Raising the CO_x methanation activity of a Ru/γ-Al_2O_3 catalyst by activated modification of metal–support interactions. *Angew. Chem. Int. Ed.* **59**, 22763–22770 (2020).
[7] J. Zhai et al., Photo-thermal coupling to enhance CO_2 hydrogenation toward CH_4 over Ru/MnO/Mn_3O_4. *Nat. Commun.* **15**, 1109 (2024).
[8] J. Ren et al., Targeting activation of CO_2 and H_2 over Ru-loaded ultrathin layered double hydroxides to achieve efficient photothermal CO_2 methanation in flow-type system. *Adv. Energy Mater.* **7**, 1601657 (2017).
[9] X. Yu et al., Layered high-entropy metallic glasses for photothermal CO_2 methanation. *Adv. Mater.* **36**, 2312942 (2024).
[10] Y. Chen et al., Cooperative catalysis coupling photo-/photothermal effect to drive Sabatier reaction with unprecedented conversion and selectivity. *Joule* **5**, 3235-3251 (2021).
[11] Kim C, et al., Energy-efficient CO_2 hydrogenation with fast response using photoexcitation of CO_2 adsorbed on metal catalysts. *Nat. Commun.* **9**, 3027 (2018).

13) The content is too abstract and generalized, and there is a lack of explanation regarding the scientific meaning of each experimental data and experimental conditions. More detailed explanation of the meaning of each experimental data should be added.

Response: We thank the reviewer for the comment. In the revised main text and Supplementary Information, we have provided detailed explanations for each experimental dataset and their scientific significance. Each result is now clearly linked to hydrogen activation, spillover efficiency, and the role of metal type and support, ensuring that all data are fully interpreted in the context of the light-driven hydrogen spillover process.

14) The text in the figures is faint, small, and difficult to read. The text in the figures should be corrected.

Response: We sincerely appreciate the reviewer's valuable suggestions. We have carefully revised all the figures in the manuscript to ensure clarity and accuracy.

Reviewer #2:

It is well established that, despite numerous studies reporting the use of metal-support structures in hydrogenation reactions and hydrogen storage, direct quantification of the hydrogen spillover effect remains largely unexplored, especially thereby impeding the rational design and optimization of catalytic systems in harsh reactions. Li and colleagues developed a well-defined quantitative framework for revealing the interaction between d-block metals and hydrogen molecule, effectively decoupling the activation and spillover regions of the hydrogen spillover effect. A mechanism is proposed with support for the Ru/TiO_{2-x} in light-driven Sabatier reaction, and the authors demonstrate how the use of the quantitative principle allows for a more stable solid catalyst. In general, this is a very timely and original manuscript that enhances researchers' understanding of the dissociation and migration of hydrogen molecules. A comprehensive suite of spectroscopic techniques (PER, XAS, FTIR, etc.) and computational approaches (DFT, AIMD) was employed to rigorously validate their conclusions. These results are impressive and essentially the manuscript reports a major advance. Therefore, I recommend the acceptance of this manuscript after minor revisions according to the comments below.

Response: We appreciate the reviewer's positive comment on our work: 'These results are impressive and the manuscript essentially reports a major advance.' For the additional comments and suggestions, we have provided detailed point-by-point responses.

1. The phrase "reducing the dissociation of the H−H bond and M−H bond" appears in the abstract; however, it is not discussed in the main text. It is recommended that the authors expand on this aspect to enhance clarity and coherence.

Response: Thank you for the reviewer's insightful comments. In this work, we decoupled the activation zone and spillover zone of the hydrogen spillover effect through quantitative analysis of photoexcitation reduction test, integrated with theoretical calculations and molecular dynamics. The nonlinear hydrogen spillover capability and rate observed for *d*-block metals originate from the intrinsic properties of the metal *d*–*s* orbitals. The partially vacant *d*–*s* orbitals can synergistically overlap with the σ and σ* orbitals of adsorbed hydrogen, forming weak antibonding states that partially weaken both the H–H and M–H bonds. This orbital-level interaction lowers the energy barriers for hydrogen dissociation and migration, providing a mechanistic explanation for the enhanced spillover behaviour of *d*-block metal. We have supplemented the relevant descriptions in the revised manuscript.

2. In the introduction, the transition from the literature review to the dual zones of the hydrogen spillover effect from lines 60−64 on page 3 is abrupt. It is recommended to refine this transition for improved logical flow. Additionally, the presentation of Fig. 1a is not sufficiently concise.

Response: We appreciate this constructive suggestion. In the revised manuscript, we have carefully refined the transition from the literature review to the discussion of the

dual zones of the hydrogen spillover effect (page 3, lines 60–64). Specifically, we added a bridging paragraph to clarify the knowledge gap in previous studies and to logically introduce our concept of decoupling the activation and spillover zones under light-driven conditions. This refinement improves the continuity of the argument and ensures a smoother logical flow.

Actions: According to the reviewer's suggestion, a bridging paragraph has been added to the revised Introduction. Besides, we also redesigned Figure 1a to enhance its clarity and presentation.

"In the hydrogen spillover process, the activation zone is where H_2 molecules are dissociated into atomic hydrogen, while the spillover zone corresponds to the migration and utilization of these species on the support surface. However, these two zones are often coupled and difficult to distinguish experimentally. In this work, we decouple the activation and spillover zones under light-driven conditions, enabling quantitative insight into their respective roles."

3. Phase and elemental characterizations for samples other than Ru are absent and should be incorporated to ensure a comprehensive analysis.

Response: We appreciate the reviewer's constructive suggestion. As presented in **Fig. R13**, TEM analysis demonstrates that all four metal are homogeneously dispersed on the TiO_{2-x} support, with no significant aggregation observed. Complementary XRD measurements confirm that the crystalline phase of the TiO_{2-x} support remains unchanged across all samples and that no additional diffraction peaks corresponding to distinct metal phases are detected, indicating the formation of well-dispersed metal without altering the host lattice structure. These combined structural and elemental characterizations provide a comprehensive validation of the uniformity and phase integrity of all metal/ TiO_{2-x} samples.

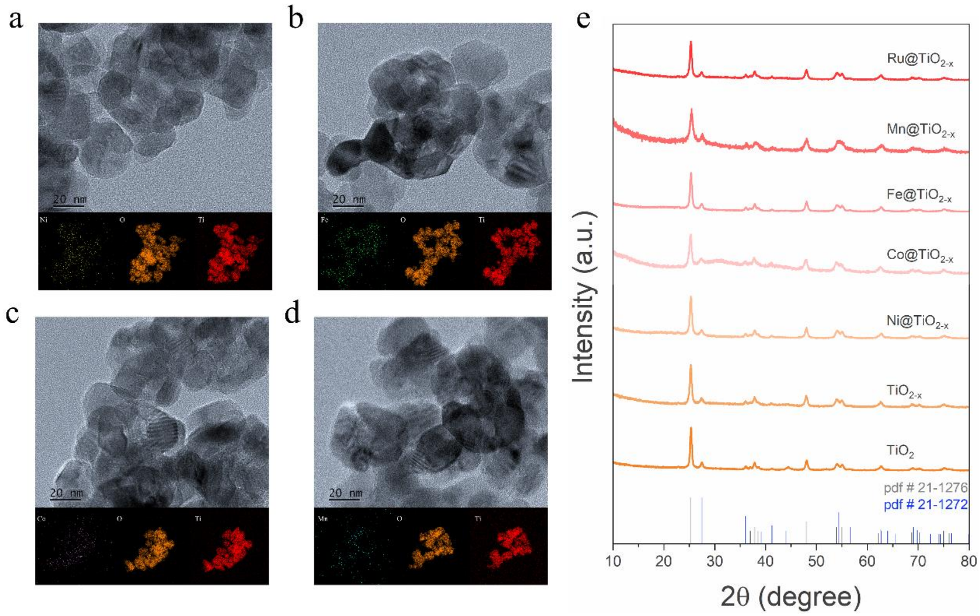

Fig. R13 | TEM images and elemental mappings of all samples. **a**, Ni/TiO_{2-x}. **b**, Fe/TiO_{2-x}. **c**, Co/TiO_{2-x}. **d**, Mn/TiO_{2-x}. **e**, XRD patterns of all the samples.

4. A detailed discussion of the molecular dynamics simulation results is lacking, and the corresponding parameters should be provided for completeness.

Response: Thank you for the reviewer's insightful comments. In the revised manuscript, we have provided a more detailed discussion of the molecular dynamics simulation results. In addition, the corresponding computational parameters have been added to the revised Supplementary Information for completeness.

Actions: According to the reviewer's suggestion, a more detailed discussion has been added to the revised main text.

"The simulation involved placing a single hydrogen molecule at the Ru site and running the system at 750 K for approximately 720 fs (Fig. 2a). Initially, H_2 molecules in the environment adsorb onto the surface of Ru clusters and rapidly dissociate, forming Ru–H bonds that correspond to the activation zone of the hydrogen spillover effect. Subsequently, the hydrogen atoms rapidly diffuse across the interface to the support, forming Ti–O–H species, which correspond to the spillover zone (Fig. 2a and Supplementary Fig. 1). Conversely, the migration of hydrogen from the support into the interior is energetically unfavourable (Fig. 2b), indicating that the hydrogen spillover primarily occurs on the interface. Combined with density functional theory (DFT) calculations, we analysed the energy and charge transfer processes: during H_2 activation on the Ru surface, charge transfers from Ru to hydrogen. One hydrogen atom (H_2) remains on the Ru cluster, while the other (H_1) spills over to the support, transferring charge to the support surface (Fig. 2c). This behavior is consistent with the activation and spillover processes observed in our PER experiments."

Actions: According to the reviewer's suggestion, a more detailed discussion about corresponding computational parameters has been added to the revised Supplementary Information.

"<u>Ab initio molecular dynamics (AIMD) simulation</u>

The work uses AIMD to simulate a system for 720 femtoseconds (fs) at 750 Kelvin (K) using a canonical ensemble, integrating equations of motion with a 1.0 fs time step. Periodic boundary conditions are applied in the *x* and *y* planes with a 15 Angstrom (Å) vacuum layer along the *z*-axis to isolate the system. The bottom layer of the simulated slab is fixed to mimic bulk constraints, allowing the rest of the atoms to move and relax."

5. In the section on the light-driven Sabatier reaction, the discoloration and protection issues of TiO_{2-x} are intriguing. However, the discussion of its protection mechanism in the main text is somewhat brief. It is recommended that the authors elaborate on this aspect to provide a detailed understanding of the mechanism.

Response: We appreciate this constructive suggestion. The manuscript now includes an expanded analysis of the mechanistic pathways underlying the discoloration and performance degradation of TiO_{2-x}. The persistent black coloration of Ru/TiO_{2-x} after hydrogenation testing is mainly due to the sustained stabilization of oxygen vacancies and Ti^{3+} species that create mid-gap defect states responsible for visible-light absorption. Ru facilitates hydrogen spillover, continuously supplying electrons to

counteract reoxidation and vacancy healing, thereby preserving the reduced electronic structure and the associated black appearance. Moreover, UV–vis–NIR absorption tests, projected density of states (PDOS) calculations, along with electron paramagnetic resonance and X-ray photoelectron spectroscopy results confirmed that the integrity of TiO_2 crystals, upon the loss of lattice oxygen, is markedly augmented through the strategic reinforcement of Ru clusters, thereby increasing their durability and mitigating deactivation processes.

6. This manuscript contains several formatting and image-related issues that require clear revision, such as inconsistent ordinate units in Fig. 1b, 2d, and 3d, as well as unclear numerical values in Fig. 2c.

Response: We appreciate this constructive suggestion. The manuscript has been thoroughly checked and the figures have been revised to correct minor inaccuracies.

Reviewer #3:

Hydrogen spillover is a general phenomenon in catalysis that is still poorly understood and requires much deeper comprehension, since it could lead to the development of more efficient hydrogen adsorption materials. By establishing some metrics, the present study has been able to distinguish between the hydrogen activation at the transition metal and its spillover on the TiO_{2-x} with high oxygen vacancies, proposing quantitative and measurable descriptors, whose relevance could be checked in other studies. Publication in Nature Communications is recommended due to the broad impact of the present approach that appears to be applicable to develop advanced hydrogenation catalysts. The authors have, however, to provide a revised version addressing the following points:

Response: We are very grateful to the reviewer for the positive comments and for noting that "By establishing some metrics, the present study has been able to distinguish between the hydrogen activation at the transition metal and its spillover on the TiO_{2-x} with high oxygen vacancies, proposing quantitative and measurable descriptors, whose relevance could be checked in other studies". For the additional comments and suggestions, we have provided detailed point-by-point responses.

1. The authors should explain in more how the hydrogen uptake was measured. From the text, line 126 it seems that one part of the measurement is carried out under irradiation and the other under dark. But then, the type of light, when the light is switched off, what happens with spillover if the measurements is made under continuous irradiation and, more important, is the hydrogen activation energy controlling the total amount of spillover hydrogen?

Response: We sincerely thank the reviewer for this insightful question. The hydrogen uptake was measured by first stabilizing the catalyst system under dark conditions to establish a baseline. Upon illumination with a 300 W Xenon lamp (CEL-PF300-T8, CEAuLight Co., Ltd., China) as the light source, with an irradiation intensity of approximately 1 W cm^{-2}, a distinct hydrogen spillover process was detected. When the light source was switched off, the spillover signal diminished immediately, demonstrating that the process is strongly light-driven and ceases without continuous energy input. Under uninterrupted illumination, the hydrogen spillover process could be consistently observed, confirming the persistent role of photoexcitation. As for the activation energy, the overall amount of spillover hydrogen is indeed influenced by the energy required for H–H bond activation. In our samples, the conditions of the system in the photoexcited state are essentially identical, so the differences in the activation and spillover performance of different samples can be quantitatively separated from the intrinsic differences among the metals.

2. How do the authors know that in the irradiation period takes place only hydrogen activation without occurring already spillover to the substrate?

Response: We sincerely appreciate the reviewer's insightful question. In the description of Fig. 1, we provided a detailed interpretation of the photoexcited reduction test. Here, we present a key summary.

Firstly, the photoexcited reduction analyser, in contrast to the temperature-programmed reduction apparatus, enables *in situ* monitoring of the temporal evolution of hydrogen activation and spillover on catalyst surfaces. Comparative analysis of metal-modified TiO_{2-x} and pristine TiO_{2-x} reveals two characteristic features: a sharp, rapid hydrogen-fragmentation peak (activation zone) and a broader, slower diffusion peak (spillover zone). The activation zone corresponds to the dissociation of H_2 molecules into atomic hydrogen (H_s) on the metal surface. Owing to the low activation energy for H_2 cleavage on metals, this step proceeds rapidly. By contrast, the spillover process entails the migration of H_s from the metal surface to the TiO_{2-x} support, a diffusion-driven phenomenon governed by the concentration gradient of hydrogen atoms. Consequently, diffusion occurs at a markedly slower rate than the initial activation.

Secondly, when focusing exclusively on the microscopic interfacial reactions, the activation process likewise entails hydrogen-atom spillover. As the flux of hydrogen molecules increases, the activation and spillover processes exhibit the characteristic dual-zone behavior described above. Accordingly, we introduce a set of descriptors including the spillover capacity (Γ), specific spillover capacity (Γ^*), activation degree (Φ) for the activation zone, and average spillover rate (κ) for the spillover zone. Quantitative comparison of these metrics clearly indicates that both the activation and spillover capacities are closely correlated with the *d*–*s* orbital hybridization strength of *d*-block metals.

3. Related with the previous point, I am missing data and discussion of the influence of the temperature, particularly in the range from ambient to 100 °C that is a common temperature range for catalysis of hydrogenation reactions, on the spillover behavior. It is necessary to indicate that the irradiation does not increase the temperature of the material.

Response: We appreciate the reviewer's comment. Our experiments aimed to investigate hydrogen spillover effect under light-driven conditions in the absence of external heating. As previously noted, when metals are deposited on semiconductor supports, they can act as nano-heaters under illumination. The hot electrons generated on the metal surface directly interact with the molecular orbitals of H_2, weakening the H–H bond and thereby enabling highly efficient hydrogen activation. In addition, these hot electrons undergo energy relaxation through both radiative and non-radiative pathways, producing localized photothermal effects on the catalyst. As shown in **Fig. R14a** (this figure corresponds to Supplementary Fig. 2a), in our photoexcited reduction experiments using a 300 W Xenon lamp (CEL-PF300-T8, CEAuLight Co., Ltd., China) with an irradiation intensity of approximately 1 W cm^{-2}, the solid catalyst was confined within a thin quartz tube, and the metal loading was controlled below 1%. Owing to the excellent photothermal conversion capability of the black TiO_{2-x} support, a significant surface temperature rise was observed under illumination. Although a small amount of metal loading can further influence the localized photothermal effect, the overall

surface temperatures of different metal/TiO_{2-x} systems remained in a narrow range of 466–468 °C (**Fig. R14b and Fig. R15,** this figure corresponds to Supplementary Fig. 2b and Supplementary Fig. 3). This relatively small temperature variation is insufficient to account for the observed hydrogen activation, underscoring that the hot electron mechanism originated from different metals, rather than simple thermal heating, plays the dominant role in this system.

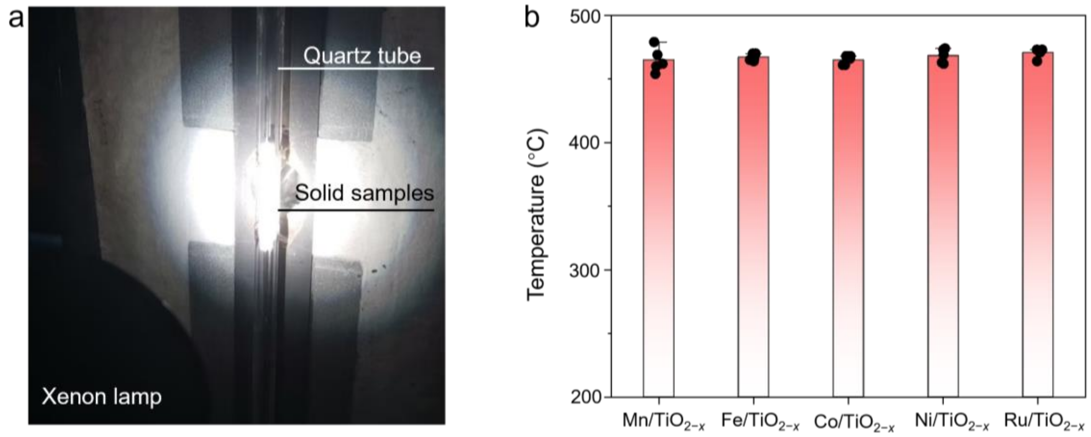

Fig. R14 | Real-time temperature monitoring for photoexcited reduction (PER) testing. a, Optical image of the local structure in the PER test. **b**, Average temperature distribution from an infrared (IR) camera under 300 W Xe lamp irradiation in the PER test. The IR images of the sample surface in the reactor were captured by an IR camera (FLUKE, TiX580) with the corresponding emissivity and were analysed by software (SmartView).

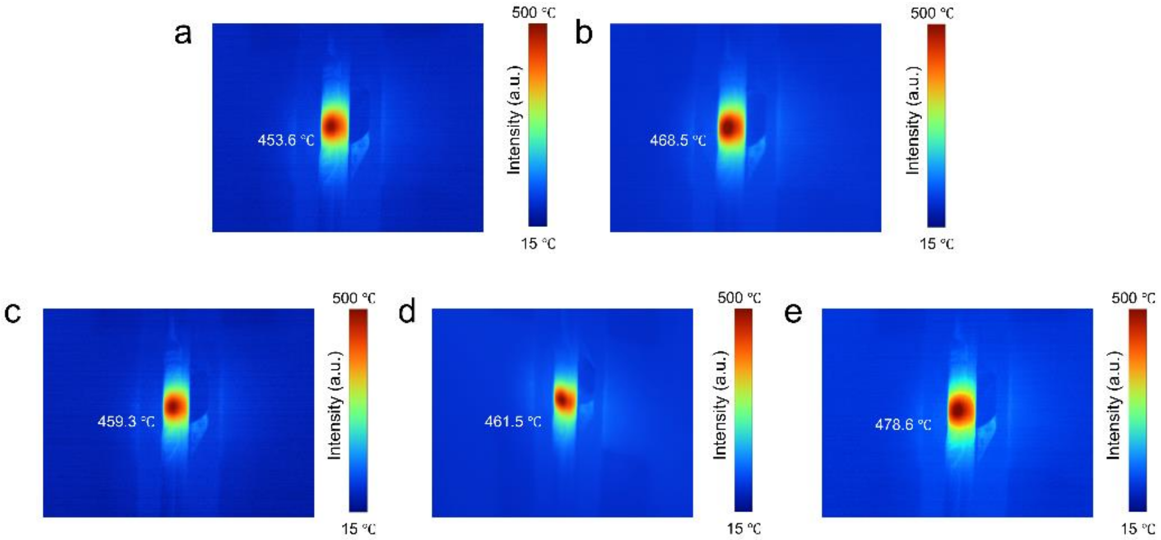

Fig. R15 | IR images of the solid catalyst recorded after the same-duration 300 W Xe-lamp irradiation. **a**, Mn/TiO_{2-x}; **b**, Fe/TiO_{2-x}; **c**, Co/TiO_{2-x}; **d**, Ni/TiO_{2-x}; **e**, Ru/TiO_{2-x}.

4. Regarding formula (1) on line 100, it is unclear what is the meaning of the intensity of the photoexcited reduction test. Is this the intensity of the light? In addition, how is the normalization factor "a" calculated? I am missing on this formula some term corresponding to each transition metal. How can various metals be compared? In my opinion, integration of the area of the peak in the plots of Fig. 1b between times t_0 and

t_1 will be sufficient. Furthermore, in this plot, rather than intensity I think that the y axis should refer to the percentage of the H_2 on the stream that is adsorbed.

Response: We thank the reviewer for this question. First, in this formula, the "I" represents the measured intensity, which reflects the signal strength recorded over time during the PER measurement. Second, "a" denotes the normalization coefficient, whose physical significance lies in converting the measured signal intensity into the corresponding quantity of gas. To establish the quantitative standard, we repeatedly introduced a fixed amount of gas and collected the associated signal responses to derive a calibration equation. The coefficient a in this equation depends solely on the properties of the detector and is independent of the metal. Third, as correctly noted by the reviewer, we use the expression $a\int_{t_0}^{t_1} I dt$ to represent the quantity of hydrogen gas.

Actions: According to the reviewer's suggestion, a detailed description has been added to the revised main text.

"a represents the normalization factor, whose physical significance lies in converting the measured signal intensity into the corresponding quantity of gas. To establish the quantitative standard, we repeatedly introduced a fixed amount of gas and collected the associated signal responses to derive a calibration equation. The coefficient a in this equation depends solely on the properties of the detector and is independent of the metal."

5. Regarding the activation metrics, as it is now written without appropriate supporting references, it gives the impression that these are original from this study. If it is not, proper credit should be given to the original study in where they were proposed. Then, the novelty of the present study will be much lower.

Response: Thanks for the reviewer's question. Our work aims to tackle the long-standing challenge of quantitatively analysing hydrogen spillover effect in light-driven reactions. Using a self-designed *in situ* photoexcited reduction device, we elucidated for the first time the origin of hydrogen spillover effect in *d*-block metals. We further decoupled the activation and spillover zones through original descriptors derived from extensive experimentation. Quantitative evaluation of hydrogen spillover effect then enabled the rational design of catalysts, which demonstrated excellent performance in flow system.

6. The selection of the TiO_2 sample used and the reasons behind its chemical modification with $NaBH_4$ to generate oxygen vacancies are unclear.

Response: We sincerely thank the reviewer for carefully evaluating our work. As a classic and well-established photocatalyst, TiO_2 has long been favoured as a model semiconductor material for fundamental studies of light-driven processes (*Nature* **1972**, 238, 37–38, first study on photocatalysis; *Nature* **2017**, 541, 68–71; *Nature* **2020**, 586, 549–554; *Science* **2021**, 371, 517–521; *Nature* **2023**, 622, 499–506; *Science* **2025**, 389, 1037–1042). Its excellent structural stability, chemical inertness, and resistance to photo corrosion ensure reliable and reproducible experiments over extended reaction

times. Its abundance, non-toxicity, and wide industrial applications in photocatalysis and environmental remediation further highlight its technological relevance and scalability. Consequently, TiO_2 is an ideal benchmark for exploring hydrogen spillover effect under light-driven conditions, and the indicators developed in such studies are highly comparable and transferable across different catalyst systems.

Action: We added a brief description in the Introduction.

"TiO_2 is a classic semiconductor with high stability, inertness, and resistance to photo corrosion, offering reliable performance and broad scalable applications"

During thermal treatment, $NaBH_4$ decomposes in situ to generate highly reactive hydrogen species (*Nat Energy* **2022**, 7, 1042–1051). The strong reducing ability of these species enables the reduction process to proceed efficiently within a relatively short time and at a lower temperature, thereby preserving the original morphology of TiO_2. Simultaneously, the active hydrogen extracts lattice oxygen and partially reduces Ti^{4+} to Ti^{3+}, creating oxygen vacancies and a defect-rich structure that modulates the electronic band structure and enhances visible-light absorption.

Action: We have added a brief description in the synthesis section of the Supplementary Information.

"Thermal treatment of TiO_2 with $NaBH_4$ generates active hydrogen species that extract lattice oxygen and partially reduce Ti^{4+} to Ti^{3+}, creating oxygen vacancies and a defect-rich structure that tailors the electronic band and enhances visible-light absorption."

7. The experimental procedure of the photoexcited CO_2 desorption tests should be explained in detail. Besides being unclear why light should desorb CO_2 from the material in the absence of any reaction, the relationship with hydrogen spillover is unclear.

Response: We sincerely appreciate the reviewer's comment. The photoexcited CO_2 desorption tests directly reveal the CO_2 activation ability of the catalyst and quantify photoactive sites while offering comprehensive insight into CO_2 hydrogenation reactions involving the hydrogen spillover effect. The principles of this technique have been detailed in our previous work (*National Science Review* **2025**, 12, nwaf174). Using molecular orbital theory, we elucidated the photoexcited molecular activation mechanism. As illustrated in **Fig. R16**, once adsorption equilibrium is established on the catalyst surface, bonding (occupied) and antibonding (unoccupied) orbitals are formed. Upon photoexcitation, the catalyst generates hot electrons that populate the antibonding orbitals of adsorbed molecules, triggering their rapid desorption. Consequently, the number of desorbed molecules serves as a quantitative indicator of hot-electron generation. In addition, hot electrons facilitate chemical transformations by promoting dissociative adsorption: they weaken the intramolecular A–A bond of the adsorbate (A_2) while simultaneously strengthening the metal–adsorbate (M–A) interaction. This synergistic effect markedly enhances molecular dissociation and activation. Moreover, we employed CO_2 hydrogenation as a model reaction to emphasize the significance of quantitative hydrogen spillover effect. Photoexcited

reduction and photoexcited desorption techniques were applied to quantify H_2 spillover and to count CO_2 photoactive sites, respectively, thereby providing deeper insight into the *in-situ* mechanism of CO_2 hydrogenation.

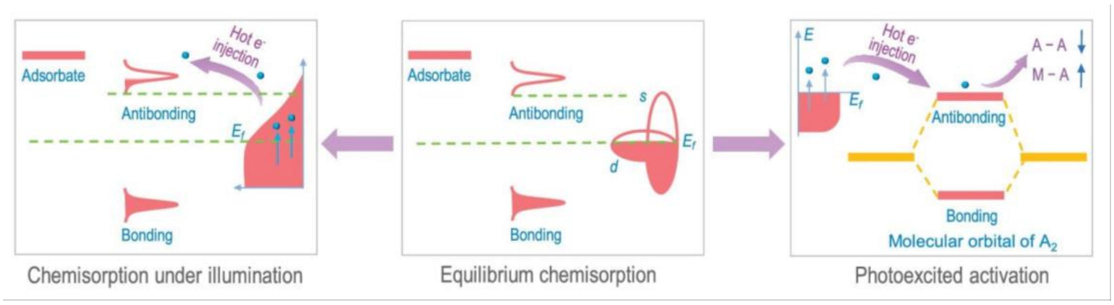

Fig. R16 | Schematic illustrations of the photoexcited molecular activation mechanism from a molecular orbital perspective.

Action: We have added a brief description in the main text and Supplementary Information, respectively.

Main text:

"In addition, the photoexcited CO_2 desorption test directly revealed the catalyst's CO_2 activation ability, with Ru/TiO_{2-x} exhibiting significantly more photoactivated sites than TiO_{2-x}, confirming its superior activity (Fig. 4b)[22]. Using molecular orbital theory, we elucidate that once adsorption equilibrium is reached on the catalyst surface, bonding (occupied) and antibonding (unoccupied) orbitals are formed. Under light excitation, hot electrons are generated and populate the antibonding orbitals of the adsorbed CO_2 molecules in the metal–semiconductor interface (Fig. 4c). This electronic injection weakens the intramolecular C=O bonds while strengthening the interaction between CO_2 and the catalyst, thereby facilitating molecular dissociation and activation. Photoexcited reduction and photoexcited desorption techniques were applied to quantify H_2 spillover and to count CO_2 photoactive sites, respectively, thereby providing deeper insight into the in-situ mechanism of CO_2 hydrogenation."

Supplementary Information:

"Photoexcited desorption test

A photoexcitation analyser previously developed by our group was employed to examine the CO_2 desorption behavior of the sample[1]. The sample was first saturated with CO_2 and then purged with a carrier gas to remove weakly adsorbed species. Subsequently, the light source was switched on to cleave the bonds of chemisorbed species, inducing desorption of adsorbates with different binding strengths. The desorbed gases were then analysed by the detector."

8. Similarly, the way in which the present measurements correlate with the light-driven Sabatier reaction should be introduced better, indicating conditions (batch, light spectrum and intensity, room temperature?), product distribution. Data $^{13}CO_2$ labeling experiment is necessary.

Response: We appreciate the reviewer's constructive suggestion. To ensure comparability of the quantitative photoexcitation data and catalytic performance results, a unified 300 W Xenon lamp (CEL-PF300-T8, CEAuLight Co., Ltd., China) with a full

spectrum irradiation intensity of approximately 1 W cm^{-2} was used and samples were stabilized in a similar temperature range. The entire experiment was conducted in a flow reactor. These detailed experimental conditions have been included in the revised Supplementary Information. As shown in **Fig. R17** (this figure corresponds to Supplementary Fig. 19), ^{13}C isotope labelling experiment has shown that a dominant peak at m/z = 17 corresponding to $^{13}CH_4$ and a weaker peak at m/z = 29 corresponding to ^{13}CO were detected, confirming that the generated CO and CH_4 result from the light-driven CO_2 reduction.

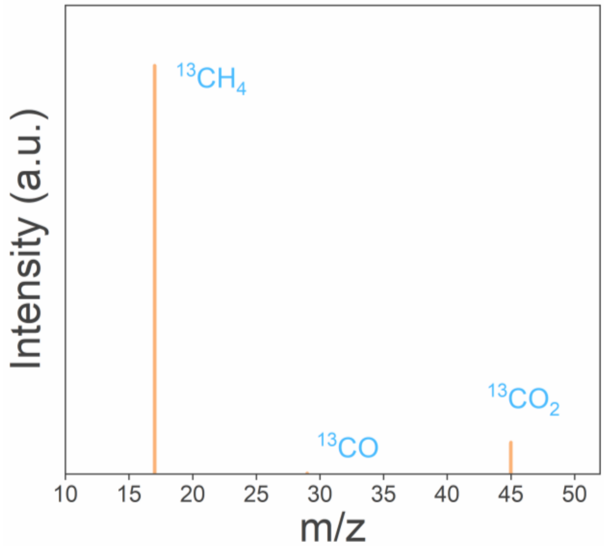

Fig. R17 | Mass spectrum of the products after $^{13}CO_2$ hydrogenation of Ru/TiO_{2-x}.

9. The last paragraph before the conclusions section repeats previous comments.

Response: We sincerely appreciate the reviewer's comment. We have revised this paragraph.

"Moreover, we would like to highlight the potential of the hydrogen spillover quantification descriptors (Fig. 4h). Beyond the discussion above, these quantitative techniques could be applied to broader research areas, such as aiding the design of light-driven catalysts for industrially important propane dehydrogenation and improving understanding of hydrogen utilization in environmental protection and human therapy"

Point-by-Point Reply to the Review Report
(Manuscript ID NCOMMS-25-14125A)

Manuscript Type: Article

Title: Origins of the hydrogen spillover effect in *d*-block metals

Author(s): Yang Li†, Yuanming Zhang†, Zhaojian Zeng†, Yong Chen, Xiaoming Xu, Zhigang Zou, Zhaosheng Li*

General response: We sincerely thank the editor, editorial staff, and all reviewers for their valuable and constructive comments, which have helped us significantly improve the quality of our manuscript. The manuscript has been carefully revised to address all the suggestions, as detailed in the point-by-point responses below. (Our response is given in blue, and some key sentences are highlighted; the corrections in the revised manuscript are highlighted).

Reviewer #1:

The manuscript has been adequately revised according to the reviewers' comments. I am satisfied with the revisions provided, so I can now agree to accept them for publication.

Response: We are very grateful to the reviewers for recommending our manuscript for acceptance.

Reviewer #2:

The authors have well addressed all the comments. I thus recommend the publication of this work as is.

Response: We sincerely appreciate the reviewer’s recommendation to accept our manuscript.

Reviewer #3:

The authors have made some progress in the revision addressing several of my previous points. However, I still think that there are some aspects that would deserve further attention. The authors should consider the following points:

Response: We sincerely thank the reviewer for recognizing the progress made in our revision and for the valuable suggestions. We have carefully addressed the remaining points by clarifying the text, adding supporting data where appropriate, and refining the discussion to improve clarity. Detailed responses are provided below.

1) In their response the authors made clear that light is always on during the duration of the hydrogen uptake by the metal nanoparticles supported on dark TiO_2. If this is the case, then, it can be assumed that the two phases, hydrogen activation and hydrogen spill over will take place simultaneously during the whole experiment. Then, how can the authors distinguish between the two phases? Even if hydrogen activation is much faster than the spill over (something to be demonstrated), both hydrogen activation and spill over will occur simultaneously after some time and a spill over phase alone will never take place in their measurements. This point has to be further clarified.

Response: We sincerely thank the reviewer for the insightful comments. Hydrogen spillover under photoexcitation involves two sequential processes: (i) hydrogen activation on the metal surface and (ii) subsequent migration of activated hydrogen atoms to the support. Under continuous illumination, both processes occur concurrently, reaching a dynamic equilibrium. Their kinetic signatures are distinct and can be reliably extracted using time-resolved photoexcited reduction (PER) experiments (**Fig. R1a**).

In the fast hydrogen activation stage, following H_2 exposure, the gas reaches a stable adsorption state on the metal surface. Upon illumination, the activation signal rises sharply, reflecting H_2 dissociation and the formation of M–H species, and rapidly reaches saturation. In the subsequent spillover stage, once the surface hydrogen is saturated, the signal increases slowly, indicating diffusion-limited migration of hydrogen to the support over a longer timescale. As shown in **Fig. R1b**, these two stages can be quantitatively distinguished by fitting the time-resolved data with a double-exponential kinetic model, yielding rate constants that clearly separate activation (k_1) and spillover (k_2). The final curve obtained after signal superposition closely matches the experimentally measured curve. Continuous illumination accelerates surface activation but does not eliminate the intrinsic kinetic distinction between the two stages. Therefore, even though both processes coexist, they can be reliably identified through their time-resolved characteristics.

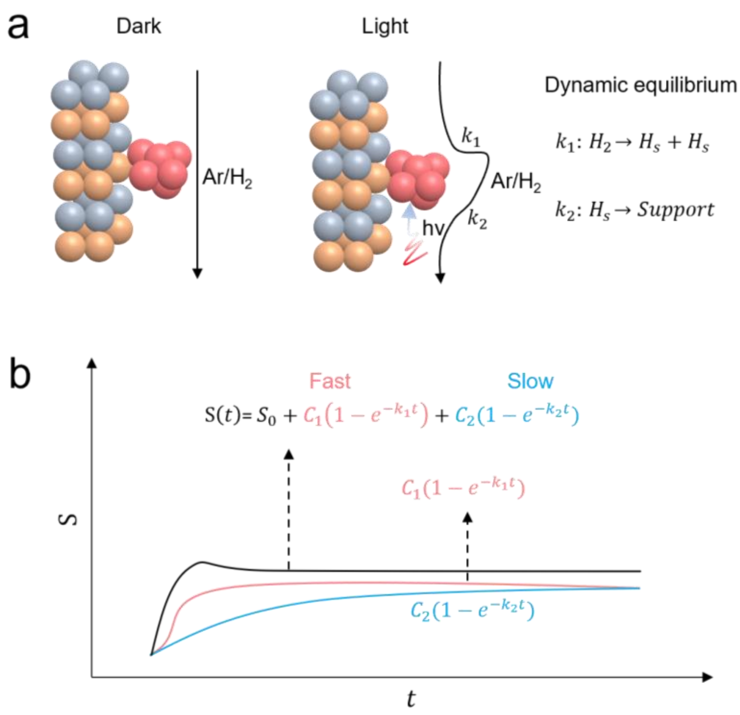

Fig. R1 | Schematic diagram of the decoupled dual-zone in photoexcited reduction tests. **a**, Dark and light processes in photoexcited reduction tests. **b**, Illustration of the fast and slow processes in the double-exponential signal dynamics.

2) If I understand properly the response to point 3, the hydrogen uptake experiments are made at temperatures above 450 °C that is much higher than those typically employed in hydrogenation reactions. Therefore, my previous comment 3 is even more important, since the interplay of activation and spill over should certainly depend on the temperature, but this influence is not considered in this study.

Response: We thank the reviewer for the insightful comment. We agree that temperature critically affects the interplay between hydrogen activation and spillover. However, this study does not focus on replicating prior reports that enhance hydrogen spillover through improved external heating. To isolate the effect of the supported metal while minimizing confounding factors such as catalyst size and dispersion, our comparative experiments were deliberately designed to vary only the metal type. The metal–semiconductor hybrids exhibit distinct temperature responses under different illumination intensities. As shown in **Fig. R2** (this figure corresponds to revised Supplementary Fig. 2), the signal resolution of both activation and spillover showed a slight decline with decreasing light intensity, yet the two regions remained clearly distinguishable. Consequently, hydrogen uptake experiments were conducted at higher light intensities (corresponding to ~450 °C) to ensure sufficient hydrogen diffusion and to probe the intrinsic spillover kinetics, rather than to mimic typical hydrogenation conditions. At lower light intensities, limited hydrogen mobility suppresses spillover, whereas higher intensities enhance both activation and migration processes, improving the temporal resolution of the measurements. These findings confirm that temperature influences only the measurement resolution, without affecting the quantitative distinction between the rapid activation and slow spillover.

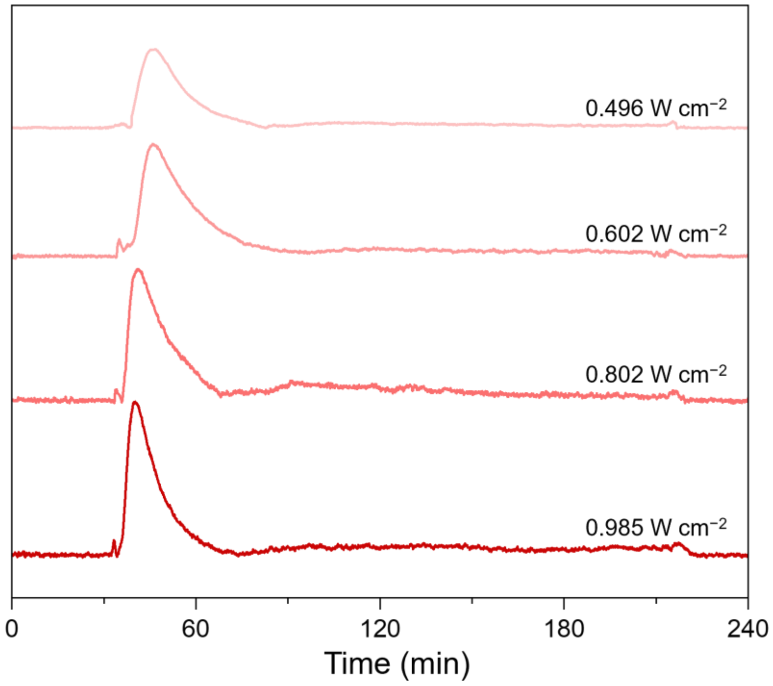

Fig. R2 | Effect of light intensity for Ru/TiO_{2-x} in photoexcited reduction tests.

Action: we have added the following statement in the revised main text to clarify this point:

"Additionally, we also carried out modulation experiments under different light intensities. With decreasing light intensity, the signal resolution of both activation and spillover showed a slight decline, yet the two regions remained clearly distinguishable (Supplementary Fig. 2). These results further validate the robustness of the photoexcited reduction test."

3) I still think that the author's response to the previous point 5 to clarify if the proposed spill over metrics is original from this paper or it is based on a prior precedent is not properly addressed. I can see that the experimental setup is new, but I would have liked to see a statement in the text saying that as far as the authors know the proposed metrics in this paper has not been reported earlier.

Response: We appreciate the reviewer's constructive comments. To the best of our knowledge, no established quantitative indicators based on a time-resolved photoexcited reduction framework have been reported. The spillover evaluation metric proposed in this work is firstly designed to quantitatively describe the kinetics of hydrogen activation and migration, extending beyond previous qualitative discussions of hydrogen spillover. We hope this clarification highlights the methodological originality of our study.

Action: we have added the following statement in the revised introduction to clarify this point:

"These two zones are assessed using previously undescribed thermodynamic-kinetic descriptors, including the spillover capacity (Γ), specific spillover capacity (Γ^*), activation degree (Φ) for the activation zone, and average spillover rate (κ) for the spillover zone."

4) When commenting about the selection of Ru nanoparticles on dark TiO_2 obtained by $NaBH_4$ treatment at high temperatures I was meaning why not use TiO_2 P25 or some other standard material without any modification. TiO_2 is widely used as support of metal nanoparticles, but the use of dark TiO_2 is considerably less common. I would like to see analogous results using Ru on P25.

Response: We sincerely appreciate the reviewer's insightful comments. Compared with pristine TiO_2 (P25), dark TiO_2 exhibits higher electronic conductivity and stronger visible-light absorption (*Science* **2011**, 331, 746–750), both of which facilitate the photoexcited hydrogen activation process (*Nat Energy* **2024**, 9, 154–162). Furthermore, the coexistence of oxygen vacancies and Ti^{3+} species is expected to enhance interfacial charge transfer and strengthen the metal–support interaction, thereby promoting hydrogen spillover. In addition, dark TiO_2 shows an increased CO_2 adsorption capacity (*Science* **2023**, 380, 644–651), which is advantageous for the subsequent hydrogenation reaction. As shown in **Fig. R3** (this figure corresponds to revised Supplementary Fig. 1), to further elucidate the influence of support types, we conducted a comparative study using Ru/TiO_2 as a reference. The results show that, although Ru/TiO_2 can also activate hydrogen, its hydrogen adsorption capacity and spillover kinetics are lower than those of Ru/TiO_{2-x}, owing to the enhanced electronic conductivity and light absorption of TiO_{2-x}.

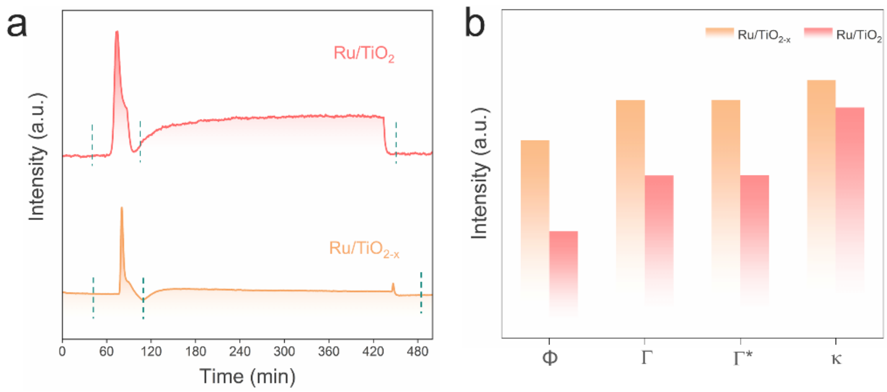

Fig. R3 | Effect of support type. **a**, Photoexcited reduction test for Ru/TiO_2 and Ru/TiO_{2-x}. **b,** Comparison of descriptors (Γ, Γ*, Φ, and κ) for Ru/TiO_2 and Ru/TiO_{2-x}.

Action: we have added the following statement in the revised main text to clarify this point:

"As shown in Supplementary Fig. 1, to further elucidate the influence of support types, we conducted a comparative study using Ru/TiO_2 as a reference. The results show that, although Ru/TiO_2 can also activate hydrogen, its hydrogen adsorption capacity and spillover kinetics are lower than those of Ru/TiO_{2-x}, owing to the enhanced electronic conductivity and light absorption of TiO_{2-x}."

5) The figure on ^{13}C labelled mass spectrum of CO_2 hydrogenation is odd. The reasons why $^{13}CH_4$ does not undergo fragmentation in the spectrometer showing peaks at m/z 16 and 15 are unclear.

<u>Response:</u> We appreciate the reviewer's comments. As shown in the **Fig. R4**, isotope mass spectrometry is a key characterization technique for confirming that the product is not affected by impurities during the CO_2 hydrogenation reaction. The signal can be slightly influenced by factors such as retention time, surface impurities, and ion fragments (*Nat. Catal.* **2020**, 3, 1034–1043). In our experiment synthesizing $^{13}CH_4$ from ^{13}C-labeled CO_2, the GC-MS spectra displayed distinct peaks corresponding exclusively to $^{13}CH_4$ (m/z = 17), ^{13}CO (m/z = 29), and $^{13}CO_2$ (m/z = 45), which are fully consistent with **Fig. R4b** and **c**. In some literature (**Fig. R4a**), weak signals at m/z = 16 or 15 may be observed near $^{13}CH_4$, likely arising from background noise, quadrupole impurities, or minor fragment ions. Nevertheless, the main peak clearly originates from $^{13}CH_4$.

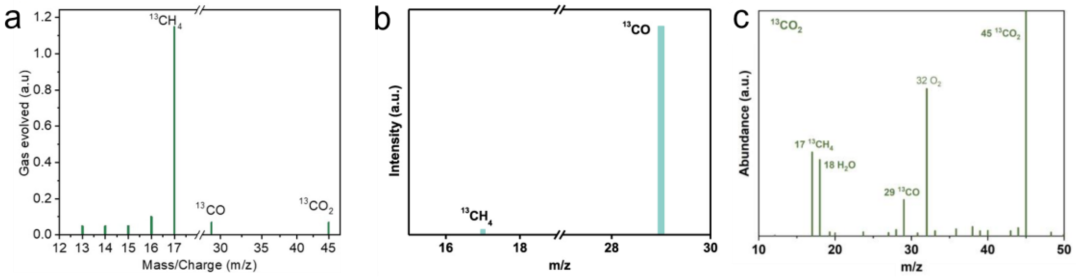

Fig. R4 | a, GC–MS spectra of isotopically labelled CO_2 hydrogenation. Data from *J. Am. Chem. Soc.* **2024**, 146, 21008–21016. **b,** GC–MS spectra of isotopically labelled CO_2 photoreduction. Data from *J. Am. Chem. Soc.* **2024**, 146, 3303–3314. **c**, GC–MS spectra of isotopically labelled CO_2 photoreduction. Data from *Angew. Chem. Int. Ed.* **2025**, 64, e202421263.

Point-by-Point Reply to the Review Report

(Manuscript ID NCOMMS-25-14125B)

Manuscript Type: Article

Title: Origins of the hydrogen spillover effect in *d*-block metals

Author(s): Yang Li†, Yuanming Zhang†, Zhaojian Zeng†, Yong Chen, Xiaoming Xu, Zhigang Zou, Zhaosheng Li*

General response:

We sincerely thank the editor, editorial staff, and all reviewers for their valuable and constructive comments, which have helped us significantly improve the quality of our manuscript. The manuscript has been carefully revised to address all suggestions, as detailed point-by-point responses below. (Our point-by-point responses are given in blue; some key sentences are highlighted in yellow, and the corrections in the revised manuscript are highlighted in cyan.)

Reviewer #3 (Remarks to the Author):

The authors have addressed most of the previous concerns and I am positive for publication. However, I still do not understand how the two phases, i.e., hydrogen activation and spillover can be differentiated when the two processes are taken places simultaneously. In fact, the authors say in lines 143-145: "As shown in Fig. 1b, a sharp, rapid hydrogen-fragmentation peak (activation zone, t0 to t1) and a broader, slower diffusion peak (spillover zone, t_1 to t_2)." As if the two processes are occurring consecutively, when it is clearly not the case according to the author's response. So, I will ask a final effort clarifying better how the authors distinguish the two process and the detailed calculation of all the parameters. I would suggest to do a detailed calculation in the supporting information for one of the catalysts before publication. Apart from this, the manuscript merits publication in Nature Commun.

Response: We are grateful to the reviewer for the insightful questions and constructive comments, which have inspired us to conduct extensive analyses to further strengthen our conclusions.

Hydrogen activation and hydrogen spillover are concurrent processes on catalyst surfaces (*Nature* **2017**, 541, 68–71). Ultrafast spectroscopic studies have shown that both processes can occur on micro- to millisecond timescales at the microscopic level; however, under practical catalytic conditions involving a large flux of hydrogen molecules, their apparent kinetics are significantly slowed, and no direct experimental approach is currently available to selectively capture these events under photoexcitation. To realistically probe hydrogen spillover involving a large population of surface hydrogen species, we developed time-resolved photoexcited reduction (PER) experiments.

Under continuous H_2/Ar flow under photoexcitation, the apparent "double-peak" feature arises from the fundamentally different kinetic characteristics of hydrogen activation and spillover. Hydrogen activation occurs on exposed metal sites and gives rise to a sharp, narrow peak ($t_0 - t_1$), whereas hydrogen/proton spillover proceeds via surface diffusion on the support, producing a broader and slower kinetic response ($t_1 - t_2$). Distinct peak widths, rise–decay slopes, and characteristic time constants enable effective kinetic deconvolution of these two contributions. We further clarify that the "activation" and "spillover" zones represent kinetically distinguishable and decouplable components, consistent with the framework of transient kinetic and pulse-response analyses.

As a representative example, consider a system containing 1,000 H_2 molecules in the environment, while the metal loaded on the catalyst surface can only interact with a maximum of 50 H_2 at a time. In the first step ($t_0 - t_1$), these 50 H_2 undergo dissociative activation on the loaded metal surface, converting into 100 free hydrogen atoms (H_s). In the second step ($t_1 - t_2$), these 100 free hydrogen atoms spillover and diffuse from the metal surface to the catalyst support surface. Simultaneously, another 50 H_2 will undergo activation on the loaded metal surface. These two processes reach a dynamic equilibrium and proceed concurrently. After that, the second step repeats continuously until all hydrogen molecules are fully activated.

Overall, the number of H_2 molecules available in the gas phase exceeds the instantaneous activation capacity of the metal sites on the catalyst surface. At any moment, only a fraction of H_2 molecules can be adsorbed and dissociatively activated on the metal, generating H_s. These H_s subsequently migrate from the metal surface to the catalyst support via hydrogen spillover. Meanwhile, newly exposed metal sites continuously activate additional H_2 molecules. So, H_2 dissociation on the metal and hydrogen spillover to the support proceed concurrently and establish a dynamic steady state, enabling the continuous activation of the hydrogen.

Action: The above analysis has been added to the main text:

"The number of H_2 molecules available in the gas phase exceeds the instantaneous activation capacity of the metal sites on the catalyst surface. At any moment, only a fraction of H_2 molecules can be adsorbed and dissociatively activated on the metal, generating H_s. These Hs subsequently migrate from the metal surface to the catalyst support via hydrogen spillover. Meanwhile, newly exposed metal sites continuously activate additional H_2 molecules. So, H_2 dissociation on the metal and hydrogen spillover to the support proceed concurrently and establish a dynamic steady state, enabling the continuous activation of the hydrogen. So, the "activation" and "spillover" zones represent kinetically distinguishable and decouplable components, consistent with the framework of transient kinetic and pulse-response analyses."

The computational details are explained by taking 1.00%-Ru/TiO_{2-x} as a representative example.

The spillover degree (Γ) was calculated according to Equation (1). Here, a represents the normalization factor, and I denotes the intensity obtained from the photoexcited reduction test. By integrating the curve over the time interval from t_0 to t_1, the value of $a\int_{t_0}^{t_1} Idt$ was determined to be 0.0369 mmol. The mass of the catalyst (m_{cat}) was calculated to be 50 mg. Accordingly, the spillover degree Γ was calculated to be 0.738 mmol g^{-1}_{cat}.

$$\Gamma = \frac{a\int_{t_0}^{t_1} Idt}{m_{cat}} \qquad (1)$$

The characteristic spillover degree (Γ^*) was calculated using Equation (2). Analogous to the calculation of Γ, m_{cat} was replaced by the mass of metal (m_{metal})). The value of m_{metal} was calculated to be 0.5 mg, yielding a Γ^* value of 73.8 mmol g^{-1}_{metal}.

$$\Gamma^* = \frac{a\int_{t_0}^{t_1} Idt}{m_{metal}} \qquad (2)$$

The activity parameter (Φ) was evaluated based on Equation (3). This value was obtained by calculating the absolute time derivative of the intensity over t_0–t_1 and taking its maximum. As a result, Φ was determined to be 0.301 i s^{-1}.

$$\Phi = max\left\{\frac{dI(t)}{dt}\right\}, t \in [t_0, t_1] \qquad (3)$$

The average spillover rate (κ) was calculated according to Equation (4). By integrating the curve over the time interval from t_1 to t_2, the value of $a\int_{t_1}^{t_2} Idt$ was determined

to be 2721.20 mol g^{-1}_{cat}. The corresponding duration $(t_2 - t_1)$ was calculated to be 346.65 min, leading to an average spillover rate κ of 7.85 mol g^{-1}_{cat} min^{-1}.

$$\kappa = \frac{a\int_{t_1}^{t_2} I dt}{t_2 - t_1} \qquad (4)$$

As shown in **Table R1** (corresponding to Supplementary Table 1), the numerical values of the descriptors for different samples, derived using this calculation method and illustrated in **Fig. 1**, are summarized below.

Table R1| Summary of the numerical values of the descriptors from **Fig. 1b** for different samples.

**Sample**	**Γ (mmol g^{-1}_{cat})**	**$Γ^*$ (mmol g^{-1}_{metal})**	**Φ (i s^{-1})**	**κ (mol g^{-1}_{cat} min^{-1})**
0.01%-Ru/TiO_{2-x}	0.052	5.2	0.034	2.24
0.05%-Ru/TiO_{2-x}	0.228	22.8	0.062	3.32
0.10%-Ru/TiO_{2-x}	0.288	28.8	0.065	4.89
0.20%-Ru/TiO_{2-x}	0.623	62.3	0.099	8.41
0.40%-Ru/TiO_{2-x}	0.646	64.6	0.192	7.65
1.00%-Ru/TiO_{2-x}	0.738	73.8	0.301	7.85

Action: The above analysis has been added to the main text, Supplementary Note and Supplementary Table 1.

We sincerely thank the reviewers once again for their valuable suggestions.
